# Triggered “Capture-and-Release” Enables a High-Affinity Rebinding Strategy for Sensitivity Enhancement in Lateral Flow Assays

**DOI:** 10.1021/acssensors.5c02665

**Published:** 2025-11-26

**Authors:** Chapman Ho, Clíona McMahon, John-Paul Ayrton, Vijay Chudasama, Michael R. Thomas

**Affiliations:** † London Centre for Nanotechnology, 4919 University College London, 17-19 Gordon Street, Bloomsbury, London WC1H 0AH, United Kingdom; ‡ Department of Biochemical Engineering, University College London, Gower Street, London WC1E 6BT, United Kingdom; § Department of Chemistry, University College London, 20 Gordon Street, London WC1H 0AJ, United Kingdom

**Keywords:** lateral flow immunoassay, cleavable linkers, site-specific protein/antibody modification, capture-and-release, signal amplification

## Abstract

Lateral flow assays (LFAs) are point-of-care devices that are known for their affordability, speed, and simplicity. However, LFA sensitivity is often limited by the need for fast associative rates between the assay components. This work presents a strategy toward reducing the demand for fast test line associative kinetics via a “capture-and-release” approach. Using HER2 protein as a model biomarker system, this methodology—termed the “AmpliFold” approach—involves the initial sequestration of analyte-bound complexes, which undergo triggered release and are rebound, using high-affinity hapten interactions, resulting in enhanced signal-to-noise detection. Using anti-HER2 Fab fragments modified with cleavable biotin linkers to achieve triggered release, the importance of linker length and bioconjugation strategy on the efficiency of analyte-bound complex release is described. Cleavable Fab fragment conjugates were combined with ‘dual-affinity’ gold nanoparticles (AuNPs) highly decorated with fluorescein-tagged anti-HER2 antibodies to facilitate signal amplification. The utility of the AmpliFold approach is demonstrated by titrating capture receptor density to modulate the signal distribution across test lines. Larger capture areas in the AmpliFold approach were shown to overcome poor capture kinetics associated with low receptor densities, achieving up to a 16-fold improvement in LFA sensitivity. The AmpliFold approach was further shown to address the poor diffusivity and surface binding kinetics of large nanoparticles in LFA systems. Using high capture receptor densities and a 150 nm AuNP example, a 12-fold sensitivity enhancement was achieved when using AmpliFold to detect the target analyte spiked into both buffer and human serum samples. Incorporated into a folding “two-strip” LFA design and performed via a multistep (capture, wash, and linker cleavage) workflow, the AmpliFold approach represents a proof-of-concept strategy that utilizes established protein modification chemistries to provide a rapid (<30 min), equipment-free, and tractable route toward enhancing LFA kinetics and sensitivity.

## Graphical abstract

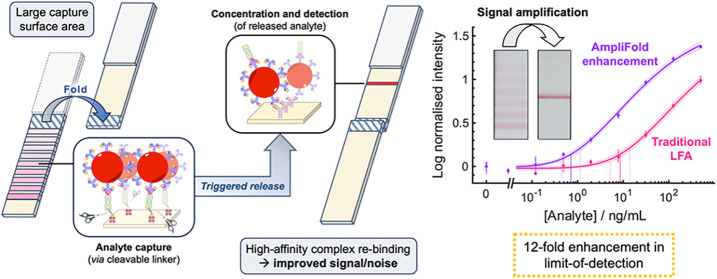

Point-of-care tests (POCT) are a class of diagnostics capable of conducting medical tests within the immediate patient vicinity.^1^ Among such tests, lateral flow assays (LFAs) have long represented an archetypal, low-cost, and rapid POCT, meeting many of the REASSURED criteria for diagnostics.^2^ Known as user-friendly and equipment-free tests, LFAs can be conducted in a variety of decentralized settings, such as clinics, pharmacies, and homes.^1^ The impact of LFAs is evident in resource-limited countries as a means of offering accessible and delocalized tests, where access to quality healthcare can be limited.^3^ The utility of LFAs was highlighted during the COVID-19 pandemic, where tests were adopted on an unprecedented scale to monitor and control the spread of SARS-CoV-2 worldwide.^4^

The performance of an LFA is often criticized as lacking in sensitivity, and thus, strategies to improve this are desirable.^5^ Realizing high sensitivity in LFAs is frequently underpinned by a need for excellent recognition components for biomarker detection.^6^ In a typical test, the time frame in which analyte flows past the test line is relatively short, presenting a small opportunity for antibodies to bind target epitopes.^7^ In sandwich LFAs, antibodies decorating the test line and nanoparticle labels must bind the analyte with fast association kinetics and high affinity to ensure maximal analyte detection and LFA sensitivity. Achieving this often necessitates screening monoclonal antibodies, which are generally expensive and time-consuming to produce, despite no guarantee of identifying candidates with high affinity and specificity toward biomarker targets.^8–10^ To this end, the ability to enhance the sensitivity of LFAs–especially where low-affinity or poorly performing antibodies fall short of clinical or early detection criteria—in a cost-effective way while maintaining maximal user-friendliness—remains crucial.

Capture-and-release strategies are ubiquitous in a variety of analytical or separation techniques, such as chromatography or immunoprecipitation, often with the aim of sequestering and enriching a target from its matrix, and subsequently triggering its release in a controlled manner.^11,12^ In LFAs, enriching biomarkers in a sample volume represents a powerful opportunity toward bypassing poor assay kinetics.^13^ In the case of nucleic acid biomarkers, recombinase polymerase amplification (RPA) has been used as a sample preprocessing step to enrich RNA targets for LFA detection.^14^ The application of similar strategies for protein targeting LFAs, however, remains limited and comparatively difficult. Key examples include an immobilized metal-affinity chromatography (IMAC) inspired ‘capture-and-release’ approach where magnetic beads were utilized to manipulate sample concentrations prior to LFA detection.^15^ Here, Bauer et al. relied on the affinity between Zn­(II) and a characteristic histidine-rich motif in the structure of a malarial HRP2 antigen to achieve noncovalent capture prior to triggered elution using competitive ligands (such as imidazole). This IMAC approach has been expanded by utilizing noncovalent aptamer binding and hexahistidine-tagged antibodies to mediate “capture-and-release” of nonhistidine- rich antigen targets in a similar fashion.^16,17^ Finally, studies by Moore et al. have shown that Zn­(II) functionalized cellulose can be used as a solid phase to perform “capture-and-release” of histidine-rich HRP2 antigens instead of magnetic beads, demonstrating improved potential for field-based testing.^18^ In these IMAC-based examples, workflows require users to elute and collect the processed analyte sample as a flowthrough volume, which is then applied to the LFA test. While efforts have sought to realize this approach with improved user-friendliness, for instance, by moving toward a cellulose membrane platform, these “capture-and-release” examples are limited to being performed externally to the LFA device itself, ultimately necessitating users to handle and operate additional equipment to produce a LFA readout. Furthermore, the ability to selectively bind protein-based biomarkers in orthogonal ways without “using up” target epitopes has been highlighted as being nontrivial, presenting additional challenges when considering “capture-and-release” across different assay targets.

In this work, we describe a novel LFA “capture-and-release” methodology that seeks to address limitations regarding poor assay binding kinetics and generate signal amplification for enhanced sensitivity. To accomplish this, we introduce a folding “two-strip” LFA architecture where target-containing sandwich immunocomplexes are initially sequestered in a capture strip via the binding of immunoproteins modified with cleavable linkers. Following triggered linker cleavage, immunocomplexes are released and subsequently rebound with higher affinity in a detection strip, where a higher density of particles and a subsequent signal are obtained. In this proof-of-concept work, a multistep workflow comprising a capture, wash, and linker cleavage step was devised. The capabilities of this “capture-and-release” strategy, which we term as the “AmpliFold” approach, are highlighted by augmenting the analyte binding region within capture strips. In the AmpliFold approach, a large surface area of capture receptors can be implemented to expand the opportunity for target capture, enabling a greater proportion of the sample analyte to be sequestered and labeled. We demonstrate that the benefits of an enhanced degree of analyte capture can be realized within our AmpliFold platform, where, through triggered linker cleavage, an increased target immunocomplex population across a large capture area is redistributed and concentrated at a narrow test line within the detection strip. For a model system, where a low capture receptor density was used to emulate poor capture affinity, the AmpliFold platform was shown to yield a 16-fold sensitivity enhancement when compared to a traditional LFA format. The application of the AmpliFold approach was further demonstrated in an LFA system utilizing large (150 nm) nanoparticle labels; specifically, large capture areas were shown to address limited binding kinetics tied to poor particle diffusivity within an AmpliFold approach, resulting in a 12-fold improvement in LFA sensitivity.

Aside from the utilization of established protein bioconjugation techniques, the proposed “capture-and-release” strategy reprises many common LFA reagents (nitrocellulose membranes, gold nanoparticles, recognition proteins).^19^ Additionally, the AmpliFold approach remains simple-to-operate, equipment-free, and rapid (<30 min), meeting much of the REASSURED criteria for POCT diagnostics.

To develop this proof-of-concept ‘capture-and-release workflow, HER2 was used as a low-cost model protein biomarker.^20–22^ It is anticipated that this work could be applied widely to different biomarker systems where the chemical modification of assay immunoprotein motifs, such as bridging disulfides and side-chain amine groups, is possible.

## Results and Discussion

In conventional LFA design, antigen binding can typically be described as a two-antibody system that relies on orthogonal epitopes to simultaneously capture and label the target analyte. In our AmpliFold approach, illustrated in Figure 1, “capture-and-release” is performed entirely across a lateral flow architecture—comprising a capture and detection strip—and utilizes additional affinity interactions to accomplish orthogonal binding mechanisms beyond that of a traditional immunoassay. In the first instance, the selection of polystreptavidin (PSA) as a capture receptor presented opportunities to develop a “capture-and-release” mechanism via a cleavable linker. The utilization of a cleavable linker provides a discrete alternative to initiating analyte release directly from the antibody–antigen complex itself, which often requires harsh immunodisruption conditions.^23^ Aside from its exceptional affinity for capturing biotin-labeled targets, the choice of PSA also represents a commonly employed LFA receptor commercially regarded for its stability and robustness against thermal and chemical influences.^24^ In a preassay step, the model target analyte, HER2 protein, was premixed with, and bound by, a modified anti-HER2 Fab fragment (Fab_HER_) and a fluorescein-tagged anti-HER2 antibody-AuNP conjugate, forming a sandwich immunocomplex structure. When run up AmpliFold capture strips, HER2-containing sandwich immunocomplexes are initially sequestered by PSA test line receptors due to chemical modification of anti-HER2 Fab fragments (Fab_HER_) with a cleavable linker bearing a biotin tag. The cleavable linker features a disulfide bond, which can undergo triggered cleavage using reducing reagents, thus enabling the release and elution of captured sandwich immunocomplexes from the initial PSA capture. Disulfide-based cleavage was selected as a promising candidate as it is well-validated within cleavable antibody-drug conjugate applications and benefits from a prevalence of commercial disulfide-containing linkers capable of enabling facile protein biotinylation.^25^ Disulfide-based cleavage chemistry was anticipated to provide suitably fast cleavage kinetics for developing a capture-and-release strategy, which could be performed under a paper microfluidic flow regime and in a comparable time frame to typical LFAs (*ca*. 10–20 min).^26^ The AmpliFold development reported in this work has built upon conventional LFA materials (such as fast-flowing nitrocellulose membranes and AuNPs), though it is envisaged that the core chemistries and concepts underlying our “capture-and-release” method are broadly applicable to various LFA configurations and optimizations.

**Figure 1:**
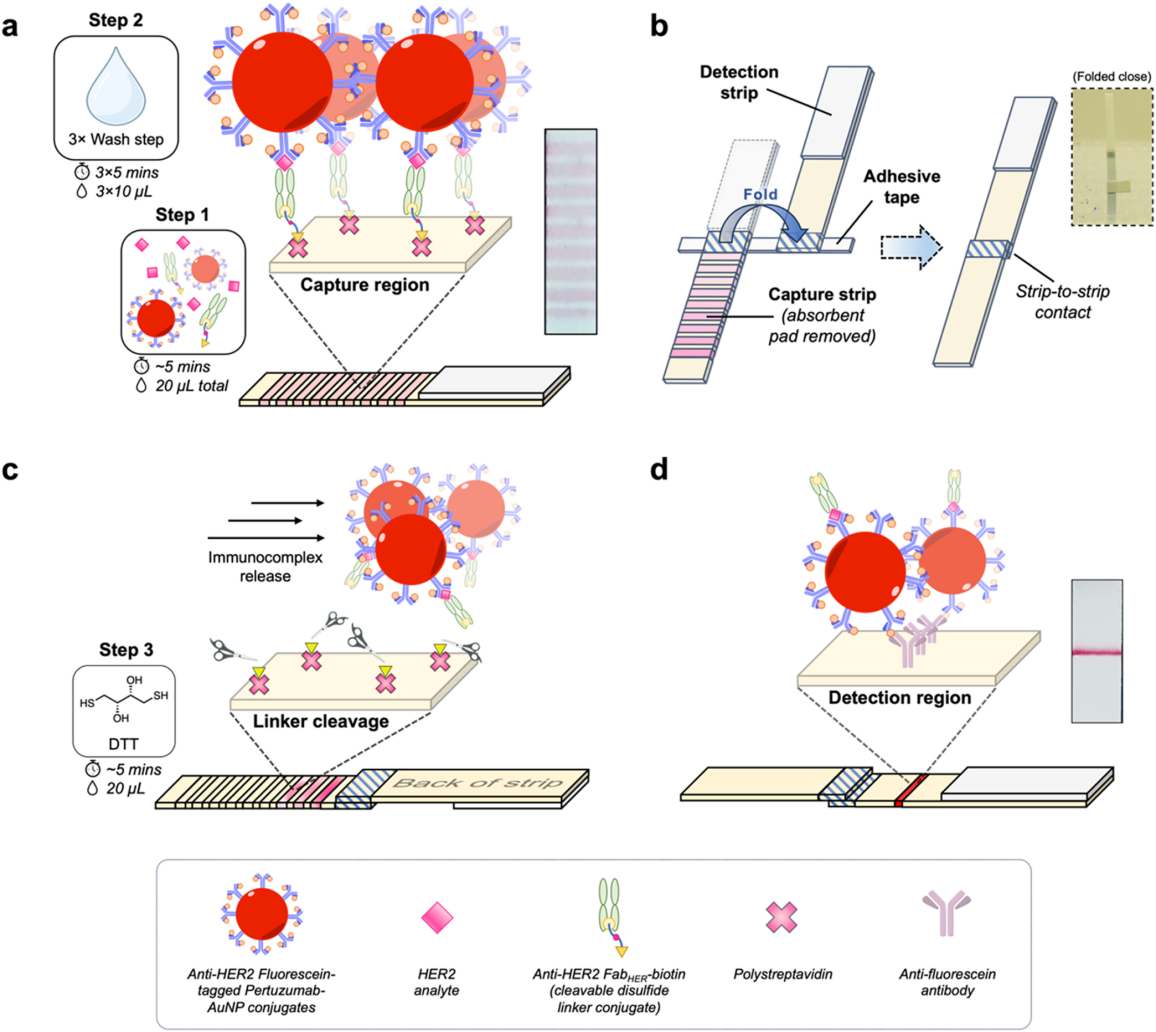
Schematic depiction of the AmpliFold capture-and-release workflow performed across a “two-strip” LFA architecture composed of a capture and detection strip. (a) In the first step, HER2 analyte is premixed and bound by anti-HER2 Fab_HER_-biotin and anti-HER2 fluorescein-tagged Pertuzumab-AuNP (FluoroPerAuNPs) conjugates. The formed sandwich immunocomplexes are captured across a large surface area of polystreptavidin (PSA) receptors in the capture strip, via the biotin-presenting cleavable disulfide linker of Fab_HER_-biotin conjugates, while unbound nanoparticles flow into the absorbent pad. As a second step, the test strip is washed thrice to remove any nonspecifically bound material from the test strip membrane into the wicking pad. (b) Assembly of the folding “two-strip” AmpliFold test, which depicts the capture and detection strips aligned on a piece of adhesive tape and the “strip-to-strip” contact initiated by folding of strips together. The absorbent pad of the capture strip is removed by manual cutting prior to AmpliFold assembly. (c) Triggered release of HER2 sandwich immunocomplexes from the capture strip via disulfide-linker cleavage by the use of a thiol-based cleavage reagent. (d) Elution from the capture strip into the detection strip and subsequent detection of released immunocomplexes over a narrow test line, which rebinds tagged nanoparticles with high affinity.

### Selection of a Model System and Development of “Capture-and-Release” Components

As a binding system, Pertuzumab and the Fab fragment of Herceptin (Fab_HER_) were selected as matched pairs for binding the HER2 protein. Pertuzumab and Herceptin are cost-effective, FDA-approved antibodies that are known to bind HER2 via different epitopes, with low nanomolar binding affinities, providing an adequate matched pair for R&D purposes.^27^ The capture protein Fab_HER_ was modified with biotin via multiple strategies, allowing us to study the influence of linker length, flexibility, and modification approach when evaluating the efficiency of “capture-and-release.” A range of thiol-cleavable commercially available lysine-reactive linkers, including sulfo-NHS-SS-biotin, NHS-SS-PEG_4_-biotin, were employed in reaction with Fab_HER_ in a nonsite-selective modification strategy, with NHS-PEG_12_-biotin as a noncleavable control (Figure 2a, parts i–iii). This generated Fab_HER_-linker-biotin conjugates **1–3**. A further strategy utilized pyridazinedione (PD) chemistry to site-specifically modify the Fab_HER_’s single solvent accessible disulfide using Br_2_PD-BCN to site-selectively install a single strained alkyne (bicyclo[6.1.0]­non-4-yne, BCN), which was then reacted with an N_3_–PEG_3_-SS-PEG_4_-biotin linker (see Figure 2b for synthesis) via a quantitative “click” chemistry reaction to generate Fab_HER_–PD-PEG_3_-SS-PEG_4_-biotin **(4)** (Figure 2c).^28^ Pyridazinedione chemistry is known in the literature for enabling reliable and controlled bioconjugation of antibody reagents, and was used here to achieve a Fab_HER_-biotin conjugate with a precise linker length, number, and modification location.^29^

**Figure 2:**
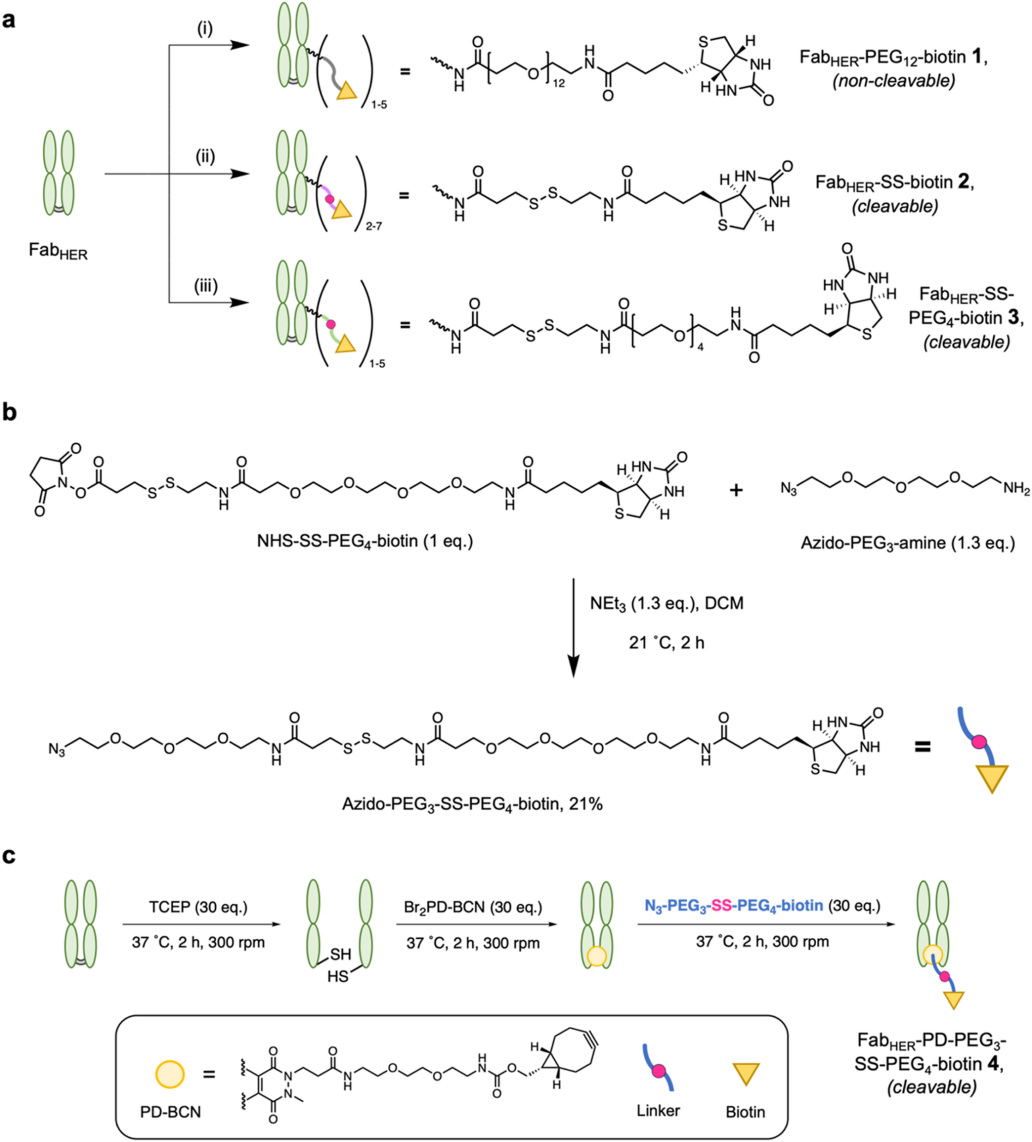
(a) Bioconjugation schematic for Fab_HER_ fragments where lysine modification was used to react protein with activated esters of (i) NHS-PEG_12_-biotin, (ii) sulfo-NHS-SS-biotin, and (iii) NHS-SS-PEG_4_-biotin. (b) Synthesis of a “clickable” and PEGylated biotin linker bearing a cleavable disulfide unit. (c) Site-specific modification of Fab_HER_ fragments via a pyridazinedione platform and subsequent linker attachment.

Pertuzumab was employed as a detection antibody for the functionalization of gold nanoparticle (AuNP) labels. Aside from binding the HER2 protein, our design criteria comprised a particle label that could be orthogonally rebound at the test line of the detection strip. The method of particle rebinding itself was anticipated to necessitate a high-affinity interaction to ensure maximal recovery of particle complexes, following release by linker cleavage, and detection with superior signal-to-noise, when compared to initial analyte capture. To develop a “dual affinity” nanoparticle label, Pertuzumab was first premodified with fluorescein isothiocyanate (FITC) to install specific and orthogonal affinity tags. The resulting fluorescein–Pertuzumab (FluoroPer) conjugates were then used to functionalize 40 nm AuNPs via passive physisorption (Figure 3a). The physisorption of FluoroPer to citrate capped 40 nm AuNPs was confirmed by UV–vis (Figure 3b) and dynamic light scattering (DLS) (Figure 3c) where upon binding of the protein, a peak plasmon shift and increase in hydrodynamic diameter of 12.1 nm, respectively, were observed. The increase in hydrodynamic diameter reflected the thickness of a protein layer consistent with expected antibody dimensions.^30^ 40 nm FluoroPerAuNPs blocked with BSA were shown to have a further increased hydrodynamic diameter, suggestive of corona formation. ζ-potential measurements (Figure 3d) indicated the blocked FluoroPerAuNPs exhibited a negative surface charge of −22.8 mV, comparable with “bare” citrate-capped AuNPs; however, during the process of FluoroPer physisorption, an intermediate surface charge closer to neutral was measured. To evaluate the affinity of these particles as a rebinding target, LFA studies were performed to benchmark the performance of FluoroPerAuNPs against a biotin–streptavidin model AuNP system. Here, nitrocellulose membranes printed with either antifluorescein or PSA test lines were used to bind and detect 40 nm FluoroPerAuNPs and biotinylated-AuNPs, respectively. As a general procedure, test line signal measurements were achieved by imaging test strips under controlled lighting conditions and using ImageJ software to extract pixel intensity values (full procedure described in the Materials and Methods section, as well as in Supporting Information Figure S31). In these experiments, 40 nm FluoroPerAuNPs were shown to be detected down to concentrations of 274 fM (95% CI of 150 to 533 fM), and 40 nm biotinylated-AuNPs down to concentrations of 94.8 fM (95% CI of 52.6 to 169 fM). While lesser than its model comparison, the detection of our FluoroPerAuNPs down to femtomolar sensitivities likely reflects a significant degree of decoration on the particle surface by FluoroPer antibodies. Given that the biotin–streptavidin interaction is commonly regarded as the highest affinity interaction, the comparable performance of FluoroPerAuNPs was rationalized based on the availability of fluorescein tags on the particle surface. Using mass spectrometry analysis, the average fluorescein-to-antibody ratio was determined to be 4:1, enabling functionalized particles to present numerous tags across their surface for rebinding by test line receptors (Supporting Information Figure S9).

**Figure 3:**
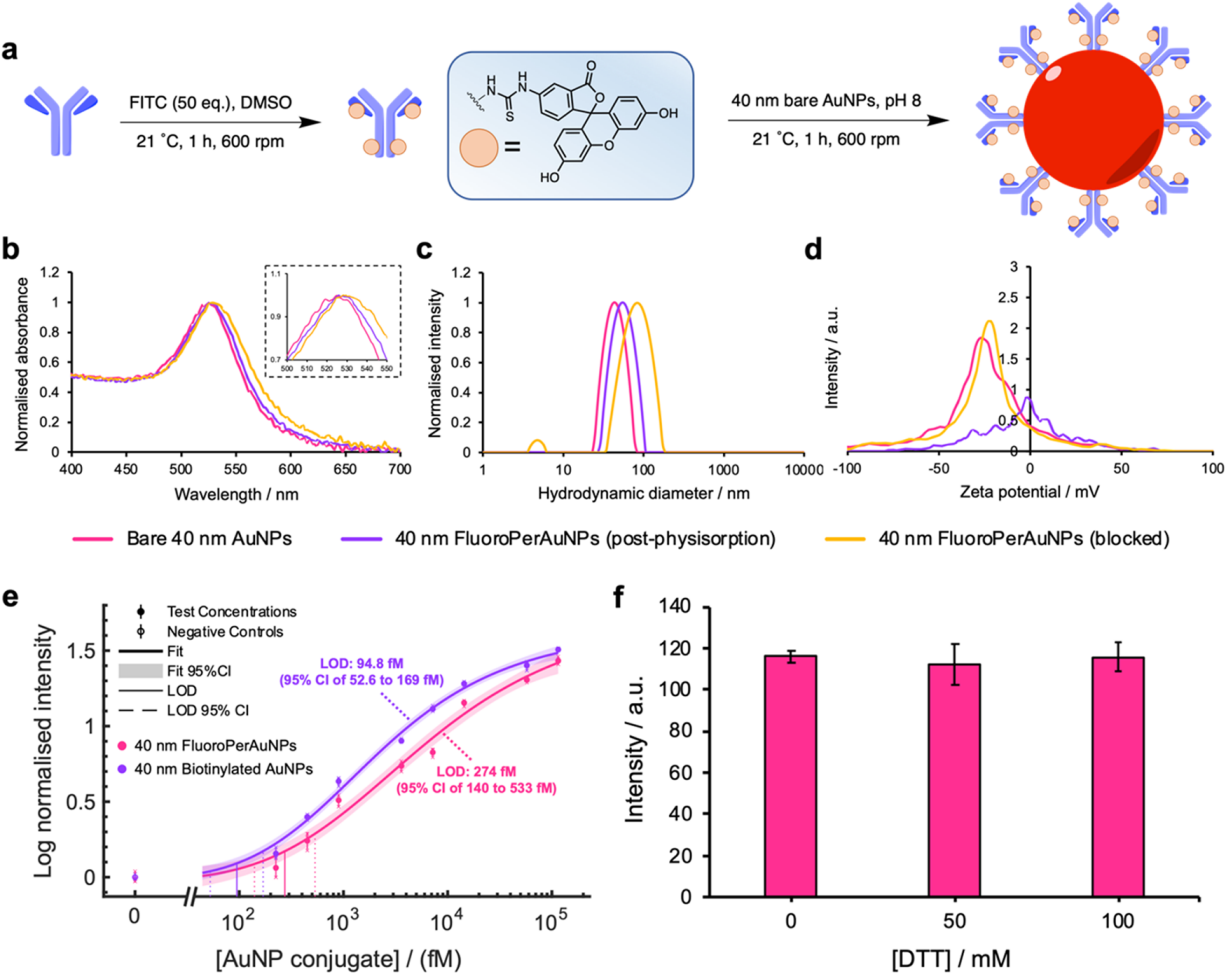
(a) Modification of the anti-HER2 detection antibody, Pertuzumab, with fluorescein isothiocyanate (FITC) and subsequent physisorption of 40 nm AuNPs with Fluoro–Pertuzumab conjugates (FluoroPer) to produce 40 nm FluoroPerAuNPs. (b) Normalized UV–vis absorbance spectra for bare 40 nm AuNPs, 40 nm FluoroPerAuNPs following physisorption, and 40 nm FluoroPerAuNPs following BSA blocking; region of interest indicated by dashed box, which compares peak maxima between bare (525 nm), physisorbed 40 nm FluoroPerAuNPs (526–530 nm), and BSA blocked 40 nm FluoroPerAuNPs (530 nm). (c) Particle sizing, by hydrodynamic diameter peak maxima, of bare 40 nm AuNPs (43.9 nm), physisorbed 40 nm FluoroPerAuNPs (56.0 nm), and BSA blocked 40 nm FluoroPerAuNPs (84.0 nm) by DLS. (d) ζ-Potential of bare 40 nm AuNPs (−27.1 mV), physisorbed 40 nm FluoroPerAuNPs (−1.91 mV), and BSA blocked 40 nm FluoroPerAuNPs (−22.8 mV) as measured by ELS. (e) Comparison of the limit-of-detection for 40 nm FluoroPerAuNPs and 40 nm biotinylated-AuNPs using test strips printed with antifluorescein pAbs and PSA, respectively. Data was collected in triplicate and was fitted using a four-parameter logistic (4PL) regression fit. (f) Mean signal intensity (*N* = 3) of antifluorescein test lines where 40 nm FluoroPerAuNPs were directly mixed with DTT in cleavage conditions prior to running.

The viability of the “dual-affinity” 40 nm FluoroPerAuNPs was further studied by observing the stability and performance of these particles in the presence of disulfide linker cleavage reagents. The incorporation of thiol-based reducing reagents, such as dithiothreitol (DTT), presented potential off-target consequences within assay reagents, such as protein denaturation and undesirable thiol-gold bond formation on the particle surface.^31^ To mimic the proposed assay workflow, FluoroPerAuNPs were premixed with DTT at pH 10 prior to detection using antifluorescein pAb printed test strips. This format enabled us to primarily assess particle stability but also the retention of functionality of the test line itself when challenged to exposure to the cleavage mixture over a prolonged 10 min period. No losses in signal intensity—compared to a negative DTT control—was observed when measuring the test line signal (Figure 3f). While the reduction of structural elements—such as antibody disulfide bridges—cannot be ruled out, these results echo findings by Shlyapnikov et al., who utilized reducing conditions to cleave disulfide-linked hydrophobic blocking agents from the surface of microarray-based immunoassays.^32^ The authors noted that no significant effect on antibody binding after a 5 min incubation period in cleavage reagent, offering similar insights into the robustness of recognition components under reducing conditions within brief assay timeframes.

### Appraisal of Linker Cleavage Efficiency and Kinetics

An ideal linker cleavage was envisaged to have high efficiency (i.e., nearly quantitative release of captured material) and to occur in a rapid time frame (10 min). To appraise both cleavage efficiency and kinetics, a simple, multistep procedure was performed where, in short, a premixture of HER2, Fab_HER_-linker-biotin, and 40 nm FluoroPerAuNPs was carried up PSA-printed membranes to generate a HER2 analyte-dependent test line signal. Following a wash step, biotin-anchored immunocomplexes were then cleaved using various concentrations of DTT in a pH 10 cleavage buffer (see Supporting Information Figure S20 for pH optimization). After 10 min, a thiol-reactive, *N*-methylmaleimide solution was then wicked through the membrane to quench the reaction. A control in the absence of DTT was used to assess the release of bound complexes via triggered linker cleavage (Figure 4a). Interestingly, linker cleavage was not as facile as anticipated. In examples featuring complexes captured via Fab_HER_-SS-biotin **(2)** (a short linker conjugate), a maximal signal loss of only 40.6% (±0.9%) was observed, despite a DTT concentration of 100 mM being used. When compared to supplementary studies using mass spectrometry (Supporting Information Figures S14–19), quantitative linker cleavage of unbound Fab_HER_-SS-biotin **(2)** proceeded at a 30-fold lower concentration of DTT within the same time frame was observed. Based on the differences between bound and unbound linker cleavage efficiencies, steric hindrance at the linker cleavage site may have been induced between the interface of large proteins—in this case, the Fab fragment and PSA.^33^

**Figure 4:**
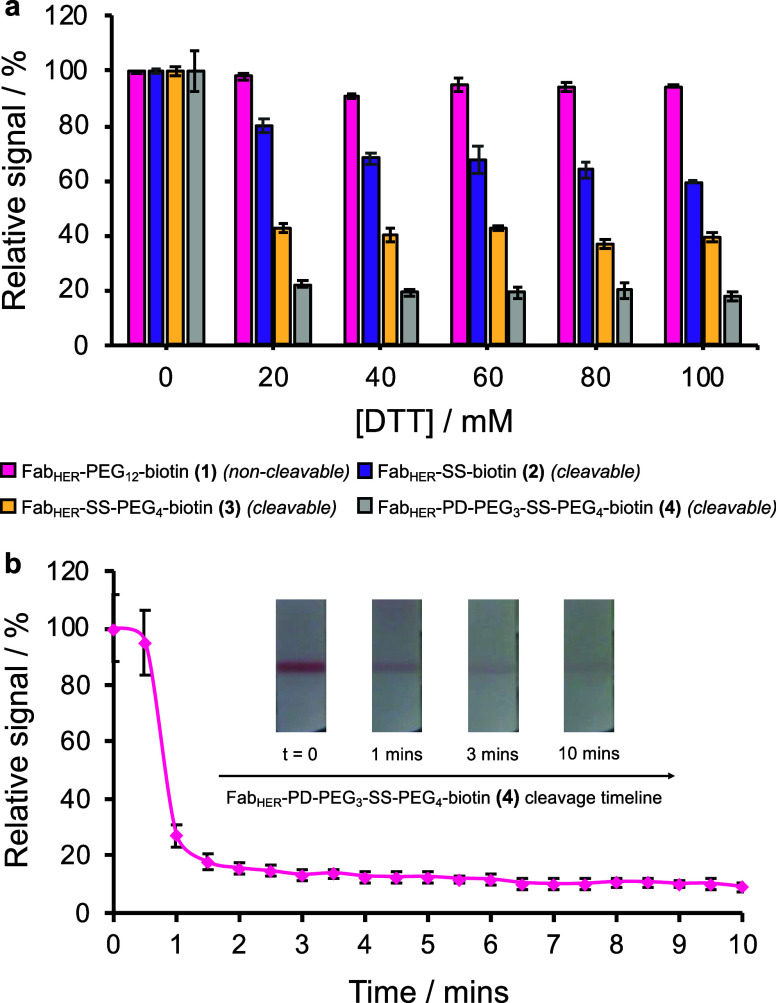
LFA-based studies investigating the release of captured complexes, via measuring the change in test line signal, upon triggered linker cleavage. (a) Cleavage efficiency, within a 10 min time frame, of Fab_HER_ conjugates featuring modifications with different biotin linker structures over increasing DTT concentrations within LFA experiments. Cleavage efficiency reflects a change in mean relative signal (*N* = 2); relative signal was calculated based on normalization of data points against a DTT negative (0 mM) control. (b) Timeline of test line cleavage, of complexes captured via Fab_HER_–PD-PEG_3_-SS-PEG_4_-biotin conjugates, from LFA test strips over a 10 min cleavage step using DTT (50 mM). Longitudinal change in mean relative intensity (*N* = 3) was plotted, where relative signal represents the normalization of data points at 0 min.

Further cleavage experiments involving an alternative modified fragment with a longer linker to the biotin entity, i.e., Fab_HER_-SS-PEG_4_-biotin **(3)**, showed an improved maximal cleavage of 63.1% (±2.5%). Here, the elongation of the PEG spacer in the linker was thought to provide some flexibility, thus alleviating some steric hindrance and improving cleavage efficiency. In an effort to achieve near-quantitative linker cleavage, Fab_HER_–PD-PEG_3_-SS-PEG_4_-biotin conjugate **(4)** was also employed. Cleavage experiments featuring this conjugate demonstrated the highest cleavage efficiency within 10 min with a reduction of signal by 82.1% (±7.4%) upon elution with 100 mM DTT. As with previous iterations, reduction of steric hindrance by linker elongation was thought to improve linker cleavage, yet this drastic enhancement in cleavage efficiency was also theorized to be due to site-specific linker installation. Previous Fab_HER_-linker-biotin iterations conjugated through lysine modifications were observed by mass spectrometry to have a large distribution of Fab_HER_-to-linker loading ratios, potentially enabling a single Fab_HER_-linker-biotin to bind numerous PSA receptors multivalently, such that multiple cleavages are required per immunocomplex for release. Using PD modification, linker installation occurs at the site of a disulfide bridge, which, for a Fab fragment, results in a 1:1 loading ratio and a single cleavage target per complex. In this way, the release of captured HER2 sandwich immunocomplexes is expected to be relatively facile, compared to constructs with the potential for multivalent anchoring to the immobilized PSA test line, and may further explain the marked difference in performance between the studied examples.

As a “noncleavable” control, Fab_HER_-PEG_12_-biotin **(1)** was similarly studied in cleavage experiments to determine that immunocomplex release and signal loss were due to specific disulfide linker cleavage. At DTT concentrations above 20 mM, a mean average signal loss of just 5.6% (±2.5%) was observed for immunocomplexes captured via the noncleavable linker (Figure 4a). Ultimately, immunocomplex release through unspecific mechanisms may, to a small extent, be beneficial in an AmpliFold context, as a greater number of immunocomplex targets become available for the detection step rebinding. Nevertheless, the findings of this experiment indicate that immunocomplex release is predominantly driven by disulfide linker cleavage and triggered by reductive conditions.

Longitudinal measurements of test line intensity over 10 min were performed and indicated that maximal cleavage of complexes formed using Fab_HER_–PD-PEG_3_-SS-PEG_4_-biotin occurred in under 5 min (Figure 4b). More specifically, 82.3% (±11.8%) test line cleavage was observed within the 1.5 min of test strips being interfaced with cleavage reagent, showcasing the rapidity of the cleavage chemistry for triggered release within LFA-relevant timeframes.

### Demonstration of “Capture-and-Release” in an AmpliFold Format

Beyond assay reagents, realizing our AmpliFold approach, comprising sequential capture, cleavage, and rebinding operations, within a single, comprehensive paper microfluidic framework necessitated the ability to perform multistep LFA processes with controlled liquid flow. For the purposes of demonstrating our “capture-and-release” approach as a proof-of-concept, a rudimentary “two-strip” assembly procedure was devised. First, the absorbent pad of the capture (PSA-printed) strip was manually cut off and discarded. The trimmed capture strip was then affixed alongside the detection (with an antifluorescein test line) strip using a thin piece of adhesive tape. Finally, the capture and detection strips were folded together, initiating membrane-to-membrane contact, which was secured by the joining of adhesive tape ends. This assembly process was performed following the capture of material and wash steps prior to the introduction of a thiol-based cleavage buffer. A full step-by-step schematic outlining this AmpliFold assembly method is shown in the Supporting Information (Figure S21). This procedure enabled us to explore the capabilities of our AmpliFold approach within a proof-of-concept design, which, at this stage, necessitated additional user steps, such as the manual removal of the capture strip absorbent pad and the transfer of strips “well-to-well.” Ultimately, it is envisioned that future iterations of an AmpliFold device could simplify the manual assembly of capture and detection strips through elaborating on the “folding cassette” designs by Yager et al., or by drawing from the designs of commercial “two-strip” devices, such as the Dual Path Platform by ChemBio Diagnostics.^34,35^

Initial development of this “two-strip” AmpliFold platform chiefly sought to address issues regarding membrane fouling of the capture strip. Detection strips of HER2-absent AmpliFold tests were noted as generating a false positive signal, following strip interfacing and the running of cleavage buffer, despite no initial capture strip signal. AmpliFold experiments utilizing ‘blank’ capture strips (no PSA printing) identified this false positive signal to be the result of residual 40 nm FluoroPerAuNPs adsorption within the capture strip membrane (Supporting Information Figure S22). To reduce the degree of membrane fouling 40 nm FluoroPerAuNPs subsequently eluted and rebound in the detection strip, we found that optimization of the number of wash steps in AmpliFold workflows to be paramount. Employing three repetitions of a wash step—each comprising 10 μL of 0.5% v/v IGEPAL solution and requiring 5 min—was shown to drastically reduce the generation of false positive signal to negligible levels, when performed prior to AmpliFold assembly and the cleavage step (Supporting Information Figure S25). Optimization of other parameters, such as assay running buffer composition and pretreatment of capture strips with membrane blocking, was shown to contribute toward reducing membrane fouling within capture strips, and improved signal-to-noise in AmpliFold tests (Supporting Information Figures S23 and S24). While these studies were shown to control membrane fouling, further work would aim to reduce the overall the repetition and duration of assay wash steps to improve the rapidity and ease of AmpliFold tests.

Preliminary studies to demonstrate the effectiveness of the AmpliFold approach, as well as identify the proportion of signal at each stage of capture, release, and detection, were conducted using blank, low (100 ng/mL), and high (1000 ng/mL) HER2 test concentrations. Using 40 nm FluoroPerAuNPs, positive analyte tests showed a high degree of recapitulation of signal when compared to the signal intensities of capture and detection (complex rebinding) strips (Figure 5a). Detection strips for high-positive HER2 tests yielded sharp test line profiles (narrow distribution of signal) when compared to relatively diffuse test line profiles of their initial capture strips. The effect of this on image analysis is exemplified in the Supporting Information (Figure S32), where test lines with narrow and sharp signal distributions were shown to demonstrate greater signal-to-noise. As shown in Figure 5a, this signal concentration effect is the result of the different binding affinities associated with the ‘target’ during either the capture or detection stage of the assay. During the capture stage, signal formation occurs due to the binding of sandwich immunocomplexes via the presentation of a single biotinylated linker by the Fab fragment conjugate. While the biotin–streptavidin binding affinity is high, this capture process is dependent on immunocomplexes on the nanoparticle colliding with the membrane in a particular orientation, which enables the biotin linker to meet a PSA receptor and initiate binding. At analyte concentrations near the LOD, we anticipate that the number of biotin linkers per nanoparticle decreases, limiting the overall binding kinetics of immunocomplex capture. During the detection step, the rebinding of released immunocomplexes now occurs through the binding of fluorescein tags on the nanoparticle surface by antifluorescein antibodies. This binding process is different from that of the capture step, as our FluoroPerAuNP conjugates demonstrate high degrees of fluorescein presentation across the entire particle surface, vastly improving the rate of successful collisions at the antifluorescein test line (Figure 3e). As a result, the rebinding of immunocomplexes during the detection step occurs with faster association kinetics and higher affinity, thus yielding sharper test line profiles and amplified signal-to-noise compared to initial capture stages.

**Figure 5:**
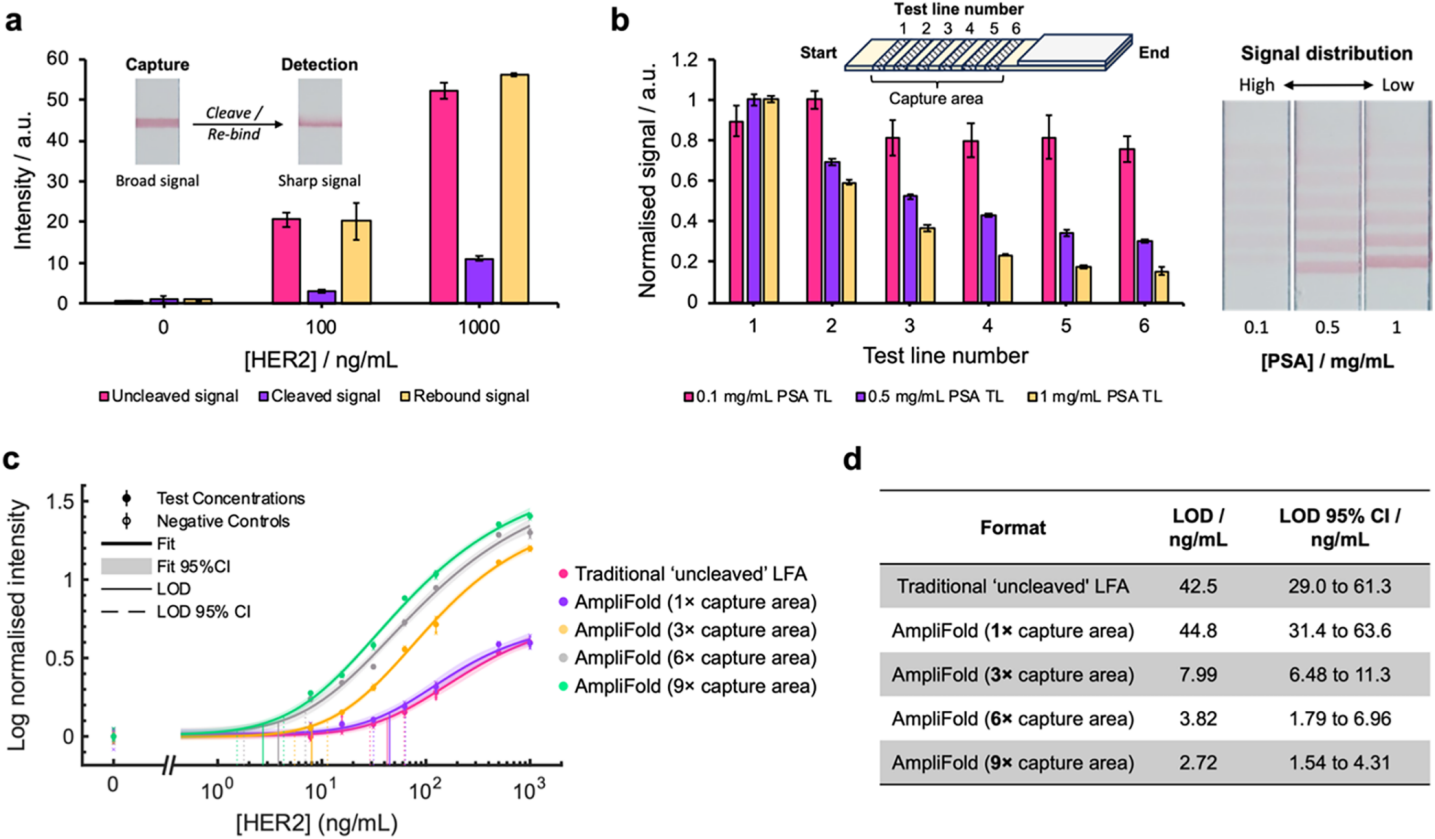
(a) Mean signal intensities (*N* = 3) of test lines following each stage of capture, cleavage, and rebinding during an AmpliFold assay at different concentrations of HER2. Annotated photographs of capture and detection strips (from 1000 ng/mL tests) are depicted to exemplify the test line signal profile observed in either step. (b) Mean normalized signal intensity (*N* = 3) across multiple PSA test lines in high-positive HER2 tests (500 ng/mL) at different printed receptor densities (low, medium, or high PSA concentrations). Schematic and photographic depictions of strips are also shown to illustrate the design of the experiment and the resultant signal distributions, respectively. (c) Dose–response data of a comparison of the performance of a traditional ‘uncleaved’ LFA format against the “capture-and-release” AmpliFold approach under model capture affinity conditions (0.1 mg/mL PSA, low capture receptor test lines). Capture strips were printed by using a varying number of test lines to probe the effects of larger capture areas on signal amplification. Data was collected in triplicate (except blanks, where *N* = 6) and plotted using a four-parameter logistic fit. (d) Table collating the limit of detection for AmpliFold assays featuring different-sized capture areas and a ‘traditional’ LFA format.

Having demonstrated the concept of “capture-and-release” within a full AmpliFold format, our attention turned toward establishing a model example where the limits of signal amplification could be explored. Here, the receptor density of printed PSA was modulated to achieve a regime in which captured sandwich immunocomplexes were uniformly distributed across a large capture area. While poor capture affinity typically detriments the sensitivity of traditional LFAs, we hypothesize that such conditions represent an opportunity where enhanced analyte recovery, over a large capture area, can be leveraged to afford maximal signal amplification when using a ‘capture-and-release’ approach.

To probe the effect of capture affinity on complex distribution, capture strips, printed with 6 test lines of varying PSA concentrations (0.1, 0.5, 1 mg/mL), were used to detect a high concentration of HER2 (500 ng/mL). When the signal intensities for each test line were examined, it was observed that strips printed with low receptor densities (0.1 mg/mL PSA test lines) resulted in similar levels of signal across all test lines. This highly uniform distribution of signal represents the ideal conditions for our “capture-and-release” approach where a large receptor area circumvents poor test line affinity. In this scenario, a significant proportion of analyte target, which would otherwise go undetected in traditional LFA formats, can be realized through the AmpliFold “capture-and-release” approach, whereby triggered release enables the distributed immunocomplexes to be rebound during detection strip in a concentrated manner. This is in contrast to assays under “high sensitivity” regimes (1 mg/mL PSA test lines), where the majority of signals tended sharply toward the front of the capture area. Under these conditions, the benefits of a large capture area were diminished, with signal distribution more closely resembling a typical LFA test line. In this scenario, the amount of additional target analyte recovered by employing a large capture area is less impactful, reducing the scope for signal amplification by “capture-and-release,” when compared to a traditional LFA format.

Using a low receptor density regime to emulate an ideal distribution of analyte capture across a large capture area, dose–response studies were conducted to evaluate the optimal surface area of printed PSA when eliciting signal amplification in “capture-and-release” AmpliFold assays (Figure 5c). In dose–response studies, serially diluted samples of HER2 were run up assays, and test line signals were measured in order to determine the minimum concentration of HER2, or the limit-of-detection (LOD), that is detectable in an LFA system. Additionally, ‘uncleaved’ LFA controls (e.g., the signal resulting from only the capture and washing steps) were employed in this experiment to directly probe the benefits of AmpliFold linker cleavage and rebinding steps in these comparisons.

In general, larger capture areas, represented by increasing numbers of test lines, resulted in notable sensitivity enhancement within an AmpliFold approach. When compared to a “traditional” LFA example (i.e., a single, uncleaved test line), no significant difference in LOD was determined when detecting HER2 using AmpliFold assays featuring single (1×) PSA test line capture strips. In this scenario, the amount of initial analyte captured is equivalent in both LFA formats, though some additional sensitivity loss is incurred by the “capture-and-release” approach due to a small percentage of uncleavable material not being detected. Beyond this, larger capture areas, represented by 3×, 6× or 9× test lines, resulted in drastic sensitivity enhancement when performing this assay methodology. When utilizing capture strips printed with 9× test lines, our AmpliFold methodology was shown to detect the protein target with a LOD of 2.72 ng/mL (95% CI of 1.54 to 4.31 ng/mL), when using 40 nm FluoroPerAuNPs. Interestingly, AmpliFold experiments were noted to yield diminishing increases in sensitivity enhancements upon increasing the area of the capture region by 3, 6, and 9× test lines. When comparing between AmpliFold experiments featuring 6× and 9× capture area AmpliFold experiments, LOD values were shown to not be significantly different (*p*-value >0.05), indicating a limited benefit when employing test line numbers above 6× in this experiment. The ideal number of capture regions will depend on the rates of specific and nonspecific interactions in the immunoassay of interest. Overall, this study demonstrated that, when operating under an ideally distributed capture regime, our novel LFA strategy can be used to generate up to a roughly 16-fold sensitivity enhancement, when compared to a “traditional” LFA format (Figure 5d).

### AmpliFold Enhancement of High-Sensitivity LFA Systems

So far, the utility of the capture-and-release strategy has been demonstrated to highlight the improvements in sensitivity that can be obtained when low-affinity surface capture limits assay performance. This same issue also affects the surface anchoring of larger nanoparticles in LFAs where reduced diffusivity and sterically hindered collisions between immunoassay components at the solid phase reduce the sensitivity. Here, we assess the performance of large nanoparticles and particularly their deployment as detection labels in the AmpliFold architecture.

The choice of nanoparticle size plays a key role in influencing the LFA sensitivity. In the case of optical labels, larger gold particles exhibit stronger light absorption and scattering properties, increasing their vibrancy for optical detection. Work by Khlebtsov et al. demonstrated that, when measuring the intensity of different AuNP sizes, the LOD for visual detection of 115 nm AuNPs was roughly 467-fold lower than that of smaller 16 nm particles, when spotted directly onto the membrane.^36^ Nanoparticle size also significantly influences a particle’s binding affinity toward its target by virtue of the available surface area for receptor functionalization.^37^ For antibody–nanoparticle conjugates, this enables greater numbers of antibodies per particle and increased particle valency when binding the target.

While larger nanoparticles represent greater assay sensitivity in theory, their benefit in practice is offset by poor kinetics. As modeled by Zhan et al., large particle complexes (>100 nm) were shown to have slower diffusion rates, which limited LFA sensitivity due to poor test line capture. The authors also noted that larger particles were associated with increased nonspecific binding, limiting their effective concentrations in assays.^38^ To demonstrate this in the context of our work, a high concentration of HER2 (500 ng/mL) was detected across test strips, printed with a capture area of 9 PSA test lines, using 20, 40, and 150 nm FluoroPerAuNPs (Figure 6a). Particles were prepared using identical physisorption conditions and similar antibody per surface area loading ratios (12.6 nm^2^/Ab), while test lines were printed using a high receptor density of PSA (1 mg/mL). When normalizing the signal intensity at each test line, we observed that assays deploying 150 nm FluoroPerAuNPs yielded a more even distribution of signal across the capture area. By comparison, the use of smaller FluoroPerAuNPs (20 and 40 nm) resulted in signal distributions that instead tended sharply toward the front of the capture area, further indicating the trade-off between particle size and capture affinity. Test strips utilizing 150 nm FluoroPerAuNPs were also shown to exhibit slight increases in normalized signal intensity above test line 7. This was due to the presence and inclusion of an elevated signal occurring at the upper edges of the test strip during image analysis. These signal artifacts were absent in HER2-negative controls, indicating perturbation of mass transport at the edge regions of test strips. We theorize that this originates from a combination of pore compression, which results from membrane cutting during test strip preparation, and the slowing of capillary action during the running of capture strips. Slower flow rates enable increased residence time and capture of larger 150 nm FluoroPerAuNP-containing sandwich immunocomplexes at PSA receptors, and these effects were particularly pronounced in the edge regions around test line 6 to 9.^39^ Using 150 nm FluoroPerAuNPs to highlight the potential of our “capture-and-release” AmpliFold approach, we hypothesized that, through using a large capture area to circumvent poor capture affinity, we could subsequently capitalize on our cleave and rebind strategy to yield significantly improved signal-to-noise. When evaluated against the “traditional” LFA approach, our optimized AmpliFold assay was shown to detect HER2 with a roughly 12-fold sensitivity enhancement at a LOD of 0.716 ng/mL (95% CI of 0.45 to 1.14 ng/mL) (Figure 6b).

**Figure 6:**
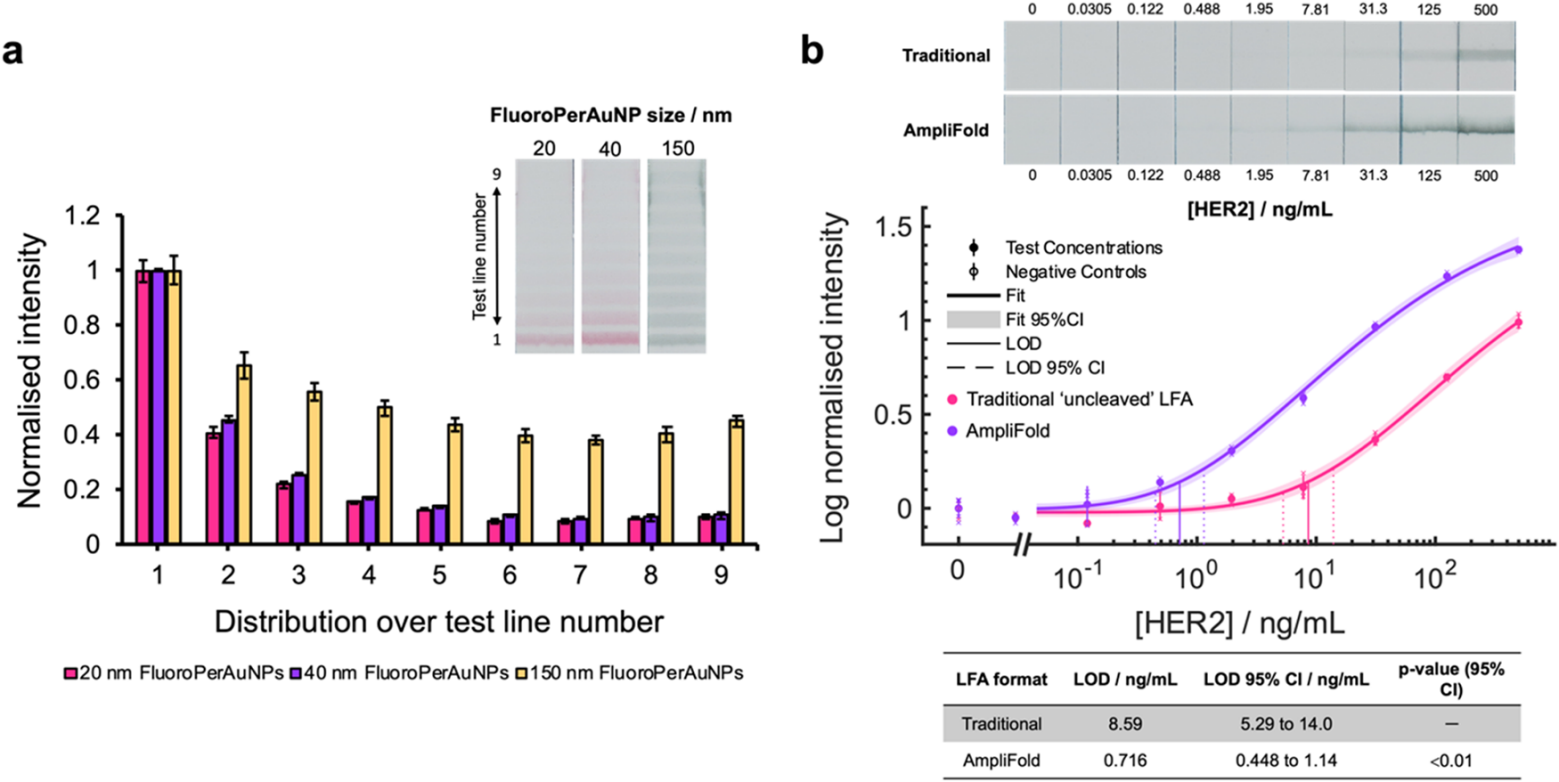
(a) Mean normalized signal intensity (*N* = 3) across multiple PSA test lines, as well as inset photographic examples, of high-positive HER2 tests (500 ng/mL) when detected with 20, 40, or 150 nm FluoroPerAuNPs at high receptor density concentrations (1 mg/mL PSA). (b) Dose–response data comparing the use of 150 nm FluoroPerAuNPs in a traditional ‘uncleaved’ LFA format and an optimized “capture-and-release” AmpliFold assay when detecting HER2 spiked in controlled buffer conditions. Inset examples of test line signals in traditional and AmpliFold LFAs for each concentration, as well as a tabulation of assay sensitivities, are shown. Data was collected in triplicate (except blanks, where *N* = 6) and plotted using a four-parameter logistic fit.

The application of our AmpliFold format was further evaluated in experiments where HER2 was spiked into human serum. Although this work aims to report AmpliFold primarily as a proof-of-concept using a model protein biomarker system, these experiments sought to provide some insight into the robustness of AmpliFold chemistries and performance when detecting analyte targets within a complex sample matrix, where the additional step of incorporating control lines was performed.

For traditional LFAs and AmpliFold capture strips, antifluorescein control lines were printed after PSA test line regions and used to bind excess 150 nm FluoroPerAuNP conjugates (examples shown in Figure 7a,b). To introduce a control line in AmpliFold detection strips, a chicken IgY (CIgY) and anti-chicken IgY (anti-CIgY) antibody binding pair was utilized, yielding a red signal at the control line (examples shown in Figure 7b). Studies of the 40 nm CIgY and anti-CIgY control line binding system were performed to characterize particle functionalization (Supporting Information Figure S13) as well as validate the performance of the binding system when integrated into AmpliFold workflows (Supporting Information Figures S27–29).

**Figure 7:**
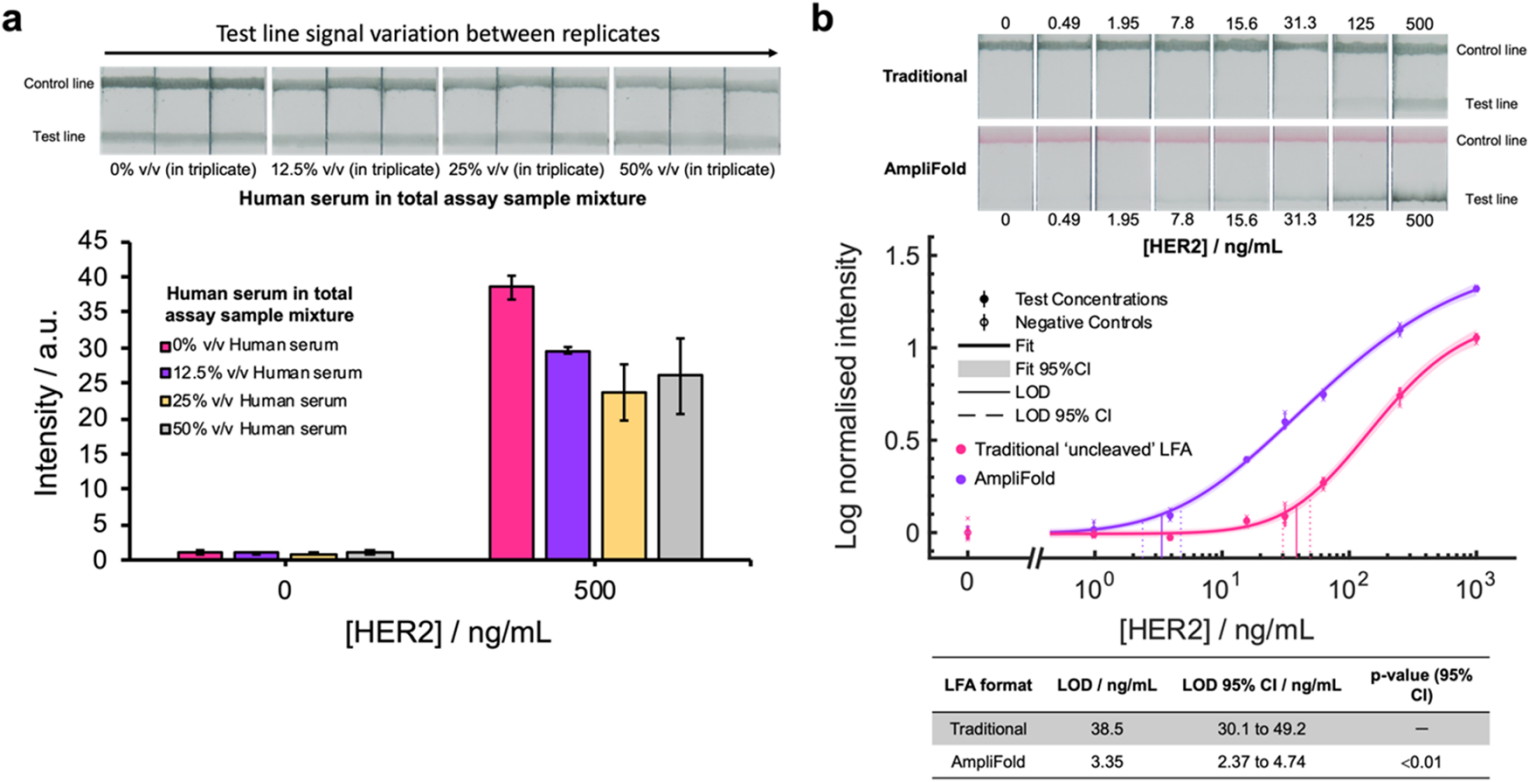
(a) Mean intensity (*N* = 3) of detection strip test lines in traditional (single test line) LFA tests where the percentage of human serum within the total sample volume, run up test strips, was varied. (b) Dose–response data comparing the use of 150 nm FluoroPerAuNPs in a traditional “uncleaved” LFA format and an optimized “capture-and-release” AmpliFold assay when detecting HER2 spiked in human serum. HER2 protein spiked in human serum was diluted by a factor of 2, using running buffer, prior to addition to microplate wells, resulting in a total of 12.5% human serum in final assay sample mixture. Data was collected in triplicate (except blanks, where *N* = 6) and plotted using a four-parameter logistic fit.

Following the incorporation of LFA control lines, we initially conducted a matrix effect study in diluted human sera to identify an appropriate sample dilution that could be used to effectively compare the traditional and AmpliFold formats of the assay. Immunoassays typically observe matrix effects in complex physiological fluids due to competing interactions between various components of the assay and the sample. In this study, the assays were run as previously, but with the volume fraction of human serum to running buffer adjusted to cover a range of human serum fractions from 0 (pure running buffer) to 50% v/v. These samples were prepared such that the concentration of HER2 added was constant (either at 0 or 500 ng/mL), allowing an assessment of how the matrix composition influences the signal-to-noise ratio of assays for matched analyte concentrations.

In traditional LFA experiments, sample mixtures containing a total of 12.5% v/v human serum were shown to produce the greatest signal-to-noise, as well as the least variation between measured test line signals (Figure 7a). AmpliFold sample mixtures containing a total of 12.5% v/v were similarly shown to result in the greatest signal-to-noise ratio, when compared to AmpliFold experiments conducted solely in running buffer conditions (Supporting Information Figure S30a), and negligible false positive test line signals (Supporting Information Figure 30b). In both LFA formats, increasing the percentage of human serum in sample mixtures was noted to have a detrimental impact of test line signal intensities for HER2-positive (500 ng/mL) tests as well as increased variation in the signals generated. We theorized that the detrimental impact on test line signal for HER2-positive tests could reflect the nonspecific interference of FluoroPerAuNP and Fab_HER_-biotin conjugate anti-HER2 binding sites by human serum components, such as albumins, hormones, enzymes, and IgG antibodies.^40^ Although endogenous free thiols could also play a role in the observed matrix effects—particularly regarding the undesirable cleavable of Fab_HER_-biotin conjugates—these would be expected to be present at drastically lower concentrations than DTT in these experiments.^41,42^ Ultimately, for a fair comparison of the traditional and AmpliFold LFA formats, the percentage of human serum in assay sample mixtures for both formats was kept the same, following this study, in order to match matrix effects in the two formats as best as possible. In subsequent dose–response experiments, a series of dilutions of HER2 spiked into human serum was prepared. A total human serum concentration of 12.5% v/v in assay sample mixtures was achieved by prediluting the typical HER2 sample volume by half, prior to mixing in with Fab_HER_-biotin **(4)** and 150 nm FluoroPerAuNP conjugate volumes. When testing serial dilutions of HER2 in human serum, our AmpliFold assay was shown to detect the target analyte with a LOD of 3.35 ng/mL (95% CI of 2.37 to 4.74 ng/mL) (Figure 7b). This was shown to be a 12-fold enhancement in sensitivity when compared to traditional LFA formats where the LOD for HER2 detection was determined to be 38.5 ng/mL (95% CI of 30.1 to 49.2 ng/mL).

Overall, in both running buffer and real matrix conditions, our AmpliFold approach was shown to greatly enhance LFA performance when using large 150 nm FluoroPerAuNP labels. Despite this, the magnitude of sensitivity enhancement observed in these studies (*ca*. 12-fold) was lesser than that of our 40 nm FluoroPerAuNP model example, where a 16-fold improvement was exhibited at minimal capture efficiency (Figure 5d). To rationalize this, we noted that the signal distribution of capture strips featuring 150 nm FluoroPerAuNPs (Figure 6a) remained comparatively uneven, when compared to the ideal uniform distribution obtained under low receptor density capture regimes (Figure 5b). We anticipate that larger particle systems, beyond what we have shown, would tend toward more evenly distributed capture regimes based on diffusivity arguments. This further highlights the potential of our “capture-and-release” AmpliFold approach in combination with existing LFA enhancements, such as using fluorescent nanodiamonds or latex microspheres, where larger particles play key roles in obtaining higher sensitivity.^43,44^ We envisage that there is also scope for future work to further optimize the workflows presented in this work, particularly with a focus on reducing the number of wash steps and the overall need for manual operation steps. Addressing these limitations is key toward affording faster assay runtimes and greater ease-of-use for future generations of AmpliFold design, ultimately bringing the technology closer toward viability at the point-of-care.

## Conclusion

In this article, we present a first-in-class “capture-and-release” strategy to tackle the limitations imposed by poor test line capture kinetics in lateral flow assay rapid tests. Analyte-bound nanoparticle complexes are initially sequestered within a capture strip and then rapidly released and rebound in a detection strip, yielding a dramatically amplified signal-to-noise ratio. Employing HER2 as a model protein biomarker system, our method involves binding the analyte with a “dual-affinity” label—employing fluorescein-tagged anti-HER2 antibodies on gold nanoparticles—and anti-HER2 Fab fragments generated using biotin-bearing cleavable linkers of varied length, flexibility, and protein modification strategies. Linkers were evaluated in lateral flow assay studies to determine the rapidity and efficiency of thiol-induced release from streptavidin bearing test lines on nitrocellulose membranes. We show that an elongated azido-PEG3-SS-PEG4-biotin linker exhibited particularly rapid release kinetics within minutes, which, when coupled with the exceptional binding capabilities of our ’dual-affinity’ labels, enabled high-affinity rebinding of analyte-bound complexes at antifluorescein test lines of detection strips. Demonstrating the efficacy of our approach, we illustrate substantial sensitivity enhancements of 16-fold in a model system probing streptavidin capture receptor density and binding kinetics. Finally, we applied our approach to address a pervasive challenge in lateral flow assay development in the form of the limited diffusivity and capture kinetics of larger nanoparticles, where, in a high-sensitivity LFA system using 150 nm “dual-affinity” labels, a 12-fold enhancement in limit of detection was realized when detecting HER2 spiked in both buffer and human serum samples. Our capture-and-release methodology emerges as a robust signal amplification technique with broad applicability across diverse LFA platforms, facilitated by adaptable protein modification strategies. Moreover, its rapid turnaround time (within 30 min) positions it as a promising diagnostic tool within the point-of-care testing landscape.

## Material and Methods

### General

All buffers and solutions were prepared using Milli-Q water unless otherwise stated. Protein LoBind microcentrifuge tubes were used to handle and contain samples containing proteins and reagents. Lateral flow assays were conducted in 96-well nonbinding flat-bottom microplates (Grenier). Unless otherwise stated, all chemical reagents and proteins were purchased from Sigma-Aldrich and did not undergo further purification before use. Concentrated buffer solutions, such as phosphate-buffered saline (PBS, Gibco) and borate buffer (Thermo Fisher), were diluted to the desired concentrations prior to use. Fab_HER_ fragments (Fab_HER_) were produced via the antibody digestion of Herceptin antibody (University College London Hospital) following a protocol by Szijj et al.^45^ Nylon syringe filters (0.45 μm; Nalgene) were used to filter solutions containing semiskimmed milk (Marvel) as a blocking agent prior to assay use.

### Particle Characterization

The UV–vis absorption spectra of nanoparticles were measured using a NanoDrop One instrument (Thermo Fisher). Dynamic light scattering (DLS) and electrophoretic light scattering (ELS) were performed using a Litesizer DLS 500 (Anton Parr). Particle size was measured by using DLS to determine hydrodynamic diameter, while ELS was used to determine ζ-potential.

### Gel Electrophoresis

Nonreducing sodium dodecyl sulfate-polyacrylamide gel electrophoresis (SDS-PAGE) was performed using precast 4–15% polyacrylamide gels (BIO-RAD) and pH 8.3 1× Tris-glycine running buffer (0.3% Tris base, 1.4% glycine, and 0.1% SDS). Samples (1 mg/mL) were premixed with Pierce 4× nonreducing sample buffer (Thermo Fisher) and incubated under agitation (80 °C, 10 min, 650 rpm). Samples (10 μL), as well as Pierce prestained protein marker (5 μL; Thermo Fisher), were loaded onto the gel, and electrophoresis was performed at a constant voltage until complete. InstantBlue Coomassie Protein Stain (Abcam) was used to stain the gel overnight; destaining was performed overnight using Milli-Q water prior to imaging.

### Fluorescein Isothiocyanate (FITC) Modification of Pertuzumab

In a typical procedure, FITC “Isomer I” (11.3 mM, 2 μL, 50 equiv, dimethyl sulfoxide [DMSO]) was added to Pertuzumab (4.5 μM, 100 μL, pH 9.2 100 mM carbonate buffer; Cambridge Bioscience) and incubated under agitation (21 °C, 1 h, 650 rpm). Following this, the reaction mixture was purified using a ZebaSpin desalting column (7 kDa molecular weight cutoff; Thermo Fisher). Fluorescein–Pertuzumab conjugates (FluoroPer) were characterized using liquid chromatography mass spectrometry (LC-MS; Agilent) to determine the average fluorescein per antibody ratio.

### Physisorption of FluoroPer Conjugates to Gold Nanoparticles (AuNPs)

The following represents a typical physisorption methodology used for the functionalization of 20, 40, and 150 nm AuNPs; exact quantities relating to each particle size can be found in the Supporting Information. Particle-to-antibody stoichiometries were maintained such that a particle surface area provided an antibody molar ratio of 12.6 nm^2^/antibody during conjugation.

To an aqueous solution of fluorescein-tagged Pertuzumab conjugates (FluoroPer), borate buffer and AuNP solution (NanoComposix) were added sequentially prior to incubation under agitation. Following this, the physisorption mixture was blocked with blocking buffer and further incubated under agitation. Blocked mixtures were then centrifuged, forming a free-flowing pellet of colloid from which the supernatant was extracted. The colloidal pellet was then washed by resuspension in blocking buffer and then centrifuged prior to supernatant extraction. This washing procedure was repeated twice more prior to the characterization of FluoroPerAuNPs.

### Linker Bioconjugation of anti-HER2 Fab Fragments via Lysine Modification

Modification of Fab_HER_ fragments, via linkers with NHS-activated ester functionality, was performed by using NHS-PEG_12_-biotin (Thermo Fisher), sulfo-NHS-SS-biotin (Thermo Fisher), and NHS-SS-PEG_4_-biotin (Nanocs). In each case, linkers were prepared in DMSO immediately before bioconjugation reactions.

To Fab_HER_ (10.5 μM, 130 μL, 1 equiv, pH 7.4 1× PBS), NHS-PEG_12_-biotin (6.12 mM, 2 μL, 9 equiv), sulfo-NHS-SS-biotin (7 mM, 2 μL, 10 equiv), or NHS-SS-PEG_4_-biotin (7 mM, 2 μL, 10 equiv) was added and incubated under agitation (21 °C, 1 h, 600 rpm). Following this, bioconjugation mixtures were purified using a ZebaSpin desalting column (7 kDa molecular weight cutoff). Fab_HER_ conjugates were characterized using LC-MS to determine the average linker per antibody ratio.

### Site-Specific Modification of Anti-HER2 Fab Fragments via Pyridazinedione (PD) Chemistry

To Fab_HER_ (10.5 μM, 128 μL, 1 equiv), prepared in 5× BBS (125 mM borate, 125 mM NaCl, 10 mM EDTA), TCEP-HCl (tris­(2-carboxyethyl)­phosphine hydrochloride, 20 mM, 2 μL, 30 equiv; ApexBio) was added and incubated under agitation (37 °C, 2 h, 300 rpm). Following this, the reduced mixture was purified and exchanged into 1× BBS using a ZebaSpin desalting column (7 kDa molecular weight cutoff). To reduced Fab_HER_, Br_2_PD-BCN (20 mM, 2 μL, 30 equiv, DMSO) was added and incubated under agitation (37 °C, 2 h, 300 rpm). The bioconjugation mixture was purified and exchanged into 1× BBS using a ZebaSpin desalting column (7 kDa molecular weight cutoff). To Fab_HER_-PD-BCN, azide-PEG_3_-SS-PEG_4_-biotin (10 mM, 2 μL, 15 equiv, DMSO) was added and incubated under agitation (37 °C, 2 h, 300 rpm). The product was purified using a ZebaSpin desalting column (7 kDa molecular weight cutoff) prior to SDS-PAGE and LC-MS characterization.

### General Half-Stick LFA Procedure

Details relating to assay components, test strip specifications, and operating procedures for particular experiments can be found in the Supporting Information. The following is a typical protocol for half-stick LFA experiments: To a microwell plate, assay components (HER2, Fab_HER_ conjugates, nanoparticles) were added and premixed (5 min). To each well, printed test strips were added and allowed to wick (5 min). Strips were added into wells containing wash solution and allowed to wick (5 min).

### Assembly of Capture and Detection Strips into An AmpliFold Format

Capture strips were cut 17 mm from the bottom of the test strip by removing the absorbent pad. As depicted in the Supporting Information (Figure S21), a strip of adhesive tape (10 mm × 30 mm) was then used to fix and assemble the AmpliFold test, such that 2 mm from the top of the capture strip interfaced the detection strip, 2 mm from the bottom, with aligned, membrane-to-membrane contact.

### AmpliFold “Capture-and-Release” Assay Procedure

Details relating to assay components, test strip specifications, and operating procedures for particular experiments can be found in the Supporting Information. The following is a typical protocol for “capture-and-release” AmpliFold experiments: To a microwell plate, assay components (HER2, Fab_HER_ conjugates, nanoparticles) were added and premixed (5 min). To each well were added printed capture strips and allowed to wick (5 min). Strips were added into wells containing wash solution and allowed to wick (5 min). Capture strips were then assembled into an AmpliFold format using antifluorescein pAb printed detection strips and added into wells containing dithiothreitol (DTT).

### LFA Imaging and Analysis

Strips were allowed to dry (21 °C, 1 h) and imaged using a Canon PowerShot G15 camera. Test line intensities were analyzed using ImageJ software using a procedure depicted in the Supporting Information (Figure S31). In summary, test strip images were converted into a greyscale image. A rectangular selection box of 500 pixels in height and 200 pixels in width was drawn to include the test line region of interest. Values relating to intensity per pixel, such that pixels were averaged (mean) across the width of the strips, were then generated and plotted. In mitigating against potential bias when determining the test line signal, test line intensity was assigned based on the maximum value within the region of interest. To avoid bias when determining the noise of the nitrocellulose membrane, the value of noise for strips was represented through the mean average of all pixels in the region of interest. The mean noise value was then subtracted from the test line intensity value to give a signal minus noise representation of the test line signal per strip. LOD calculations were determined through four-parameter logistic fitting models which utilized software by Miller et al. and were based on methods by Holstein et al.^46,47^

## Supplementary Material



## Notes

Mass spectra of native Fab_HER_ and Fab_HER_-linker conjugates **(1–3)**; mass spectra and SDS-PAGE gel of Fab_HER_, Fab_HER_-PD-BCN­(endo) and Fab_HER_–PD-PEG_3_-SS-PEG_4_-biotin **(4)**; mass spectra of native Pertuzumab and FluoroPer conjugates; exemplar quantities, UV–vis spectra, particle size measurements, and ζ-potential measurements for 20, 40, and 150 nm FluoroPerAuNPs; exemplar quantities, UV–vis spectra, particle size measurements, and ζ-potential measurements for 40 nm biotinylated-AuNPs; exemplar quantities, UV–vis spectra, particle size measurements, and ζ-potential measurements for 40 nm CIgY-AuNPs; synthesis of azido-PEG_3_-SS-PEG_4_-biotin and characterization by ^1^H NMR and ^13^C NMR spectroscopy in CDCl_3_, IR spectroscopy, low-resolution and high-resolution mass spectrometry; mass spectrometry observed linker cleavage for Fab_HER_-SS-biotin **(2)** by DTT; cleavage buffer pH optimization for linker cleavage within LFA; depicted assembly of capture and detection strips into an AmpliFold format; AmpliFold optimization for the reduction of membrane fouling; AmpliFold optimization of detection strip antifluorescein pAb test line concentration; LFA optimization for the binding of 40 nm CIgY-AuNPs; LFA image analysis procedure using ImageJ; influence of test line signal distribution toward signal-to-noise ratio during image analysis; detailed methods for printing of test lines, capture strip blocking, mass spectrometry observed linker cleavage, and LFA and AmpliFold studies (PDF)

## References

[1] Price C. P. (2001). Regular
Review: Point of Care Testing. BMJ.

[2] Land K. J., Boeras D. I., Chen X., Ramsay A. R., Peeling R. W. (2019). REASSURED
Diagnostics to Inform Disease Control Strategies, Strengthen Health
Systems and Improve Patient Outcomes. Nat. Microbiol..

[3] Turbé V., Herbst C., Mngomezulu T., Meshkinfamfard S., Dlamini N., Mhlongo T., Smit T., Cherepanova V., Shimada K., Budd J., Arsenov N., Gray S., Pillay D., Herbst K., Shahmanesh M., McKendry R. A. (2021). Deep Learning of HIV Field-Based Rapid Tests. Nat. Med..

[4] The ACT-Accelerator: Two Years of Impact, World Health Organization 2022 1-44.

[5] Deeks, J. J. ; Singanayagam, A. ; Houston, H. ; Sitch, A. J. ; Hakki, S. ; Dunning, J. ; Lalvani, A. SARS-CoV-2 Antigen Lateral Flow Tests for Detecting Infectious People: Linked Data Analysis. BMJ 2022, 376. 10.1136/bmj-2021-066871. PMC8864475 35197270

[6] Van Amerongen, A. ; Veen, J. ; Arends, H. A. ; Koets, M. Lateral Flow Immunoassays. In Handbook of Immunoassay Technologies; Academic Press, 2018; pp 157–182 10.1016/B978-0-12-811762-0.00007-4.

[7] Loynachan C. N., Thomas M. R., Gray E. R., Richards D. A., Kim J., Miller B. S., Brookes J. C., Agarwal S., Chudasama V., McKendry R. A., Stevens M. M. (2018). Platinum
Nanocatalyst Amplification:
Redefining the Gold Standard for Lateral Flow Immunoassays with Ultrabroad
Dynamic Range. ACS Nano.

[8] Cate, D. M. ; Hsieh, H. V. ; Glukhova, V. A. ; Bishop, J. D. ; Gleda Hermansky, H. ; Barrios-Lopez, B. ; Grant, B. D. ; Anderson, C. E. ; Spencer, E. ; Kuhn, S. ; Gallagher, R. ; Rivera, R. ; Bennett, C. ; Byrnes, S. A. ; Connelly, J. T. ; Dewan, P. K. ; Boyle, D. S. ; Weigl, B. H. ; Nichols, K. P. Antibody Screening Results for Anti-Nucleocapsid Antibodies towards the Development of a SARS-CoV-2 Nucleocapsid Protein Antigen Detecting Lateral Flow Assay ChemRxiv 2020, No. 2 10.26434/chemrxiv.12709538.v1. PMC8482319 34608447

[9] Klutz S., Holtmann L., Lobedann M., Schembecker G. (2016). Cost Evaluation
of Antibody Production Processes in Different Operation Modes. Chem. Eng. Sci..

[10] Boehringer H. R., O’farrell B. J. (2021). Lateral Flow Assays in Infectious
Disease Diagnosis. Clin. Chem..

[11] Shen, C.-H. Quantification and Analysis of Proteins. In Diagnostic Molecular Biology; Academic Press, 2019; pp 187–214 10.1016/B978-0-12-802823-0.00008-0.

[12] Stewart, A. ; Fisher, R. A. Co-Immunoprecipitation. In Methods in Cell Biology; Elsevier, 2012; Vol. 112, pp 33–54 10.1016/B978-0-12-405914-6.00002-0.

[13] Deng Y., Jiang H., Li X., Lv X. (2021). Recent Advances in
Sensitivity Enhancement for Lateral Flow Assay. Microchim. Acta.

[14] Cherkaoui D., Huang D., Miller B. S., Turbé V., McKendry R. A. (2021). Harnessing Recombinase Polymerase Amplification for
Rapid Multi-Gene Detection of SARS-CoV-2 in Resource-Limited Settings. Biosens. Bioelectron..

[15] Bauer W. S., Richardson K. A., Adams N. M., Ricks K. M., Gasperino D. J., Ghionea S. J., Rosen M., Nichols K. P., Weigl B. H., Haselton F. R., Wright D. W. (2017). Rapid Concentration
and Elution of
Malarial Antigen Histidine-Rich Protein II Using Solid Phase Zn­(II)
Resin in a Simple Flow-through Pipette Tip Format. Biomicrofluidics.

[16] Kantor A. G., Markwalter C. F., Nourani A., Wright D. W. (2021). An Antibody-Free
Dual-Biomarker Rapid Enrichment Workflow (AnDREW) Improves the Sensitivity
of Malaria Rapid Diagnostic Tests. Anal. Biochem..

[17] Bauer W. S., Gulka C. P., Silva-Baucage L., Adams N. M., Haselton F. R., Wright D. W. (2017). Metal Affinity-Enabled
Capture and Release Antibody
Reagents Generate a Multiplex Biomarker Enrichment System That Improves
Detection Limits of Rapid Diagnostic Tests. Anal. Chem..

[18] Moore C. P., Pieterson K., DeSousa J. M., Toote L. E., Wright D. W. (2021). Characterization
and Utility of Immobilized Metal Affinity-Functionalized Cellulose
Membranes for Point-of-Care Malaria Diagnostics. J. Chromatogr. B.

[19] Parolo C., Sena-Torralba A., Bergua J. F., Calucho E., Fuentes-Chust C., Hu L., Rivas L., Álvarez-Diduk R., Nguyen E. P., Cinti S., Quesada-González D., Merkoçi A. (2020). Tutorial:
Design and Fabrication of Nanoparticle-Based Lateral-Flow Immunoassays. Nat. Protoc..

[20] Creamer A., Fiego A. Lo., Agliano A., Prados-Martin L., Høgset H., Najer A., Richards D. A., Wojciechowski J. P., Foote J. E. J., Kim N., Monahan A., Tang J., Shamsabadi A., Rochet L. N. C., Thanasi I. A., de la
Ballina L. R., Rapley C. L., Turnock S., Love E. A., Bugeon L., Dallman M. J., Heeney M., Kramer-Marek G., Chudasama V., Fenaroli F., Stevens M. M. (2023). Modular Synthesis
of Semiconducting Graft Copolymers to Achieve “Clickable”
Fluorescent Nanoparticles with Long Circulation and Specific Cancer
Targeting. Adv. Mater..

[21] Ye L., Xu X., Qu A., Liu L., Xu C., Kuang H. (2024). Quantitative
Assessment of the Breast Cancer Marker HER2 Using a Gold Nanoparticle-Based
Lateral Flow Immunoassay. Nano Res..

[22] Ranganathan V., Srinivasan S., Singh A., DeRosa M. C. (2020). An Aptamer-Based
Colorimetric Lateral Flow Assay for the Detection of Human Epidermal
Growth Factor Receptor 2 (HER2). Anal. Biochem..

[23] Kimple M. E., Brill A. L., Pasker R. L. (2013). Overview of Affinity
Tags for Protein
Purification. Curr. Protoc. Protein Sci..

[24] Chilkoti A., Stayton P. S. (1995). Molecular Origins
of the Slow Streptavidin—Biotin
Dissociation Kinetics. J. Am. Chem. Soc..

[25] Bargh J. D., Isidro-Llobet A., Parker J. S., Spring D. R. (2019). Cleavable Linkers
in Antibody-Drug Conjugates. Chem. Soc. Rev..

[26] Nagy P. (2013). Kinetics and
Mechanisms of Thiol-Disulfide Exchange Covering Direct Substitution
and Thiol Oxidation-Mediated Pathways. Antioxidants
Redox Signal..

[27] Lua W. H., Gan S. K. E., Lane D. P., Verma C. S. (2015). A Search for Synergy
in the Binding Kinetics of Trastuzumab and Pertuzumab Whole and F­(Ab)
to Her2. npj Breast Cancer.

[28] Chudasama V., Smith M. E. B., Schumacher F. F., Papaioannou D., Waksman G., Baker J. R., Caddick S. (2011). Bromopyridazinedione-Mediated
Protein and Peptide Bioconjugation. Chem. Commun..

[29] Bahou C., Chudasama V. (2022). The Use of
Bromopyridazinedione Derivatives in Chemical
Biology. Org. Biomol. Chem..

[30] Tan Y. H., Liu M., Nolting B., Go J. G., Gervay-Hague J., Liu G. (2008). A Nanoengineering Approach
for Investigation and Regulation of Protein
Immobilization. ACS Nano.

[31] Awotunde O., Okyem S., Chikoti R., Driskell J. D. (2020). Role of Free Thiol
on Protein Adsorption to Gold Nanoparticles. Langmuir.

[32] Shlyapnikov Y. M., Malakhova E. A., Shlyapnikova E. A. (2021). Improving
Immunoassay Performance
with Cleavable Blocking of Microarrays. Anal.
Chem..

[33] Su D., Zhang D. (2021). Linker Design Impacts Antibody-Drug Conjugate Pharmacokinetics
and
Efficacy via Modulating the Stability and Payload Release Efficiency. Front. Pharmacol..

[34] Fu E., Liang T., Houghtaling J., Ramachandran S., Ramsey Sa., Lutz B., Yager P. (2011). Enhanced Sensitivity
of Lateral Flow Tests Using a Two-Dimensional Paper Network Format. Anal. Chem..

[35] Gunasekera M., Narine M., Ashton M., Esfandiari J. (2015). Development
of a Dual Path Platform (DPP) Immunoassay for Rapid Detection of Candida
Albicans in Human Whole Blood and Serum. J.
Immunol. Methods.

[36] Khlebtsov B. N., Tumskiy R. S., Burov A. M., Pylaev T. E., Khlebtsov N. G. (2019). Quantifying
the Numbers of Gold Nanoparticles in the Test Zone of Lateral Flow
Immunoassay Strips. ACS Appl. Nano Mater..

[37] Gasperino D., Baughman T., Hsieh H. V., Bell D., Weigl B. H. (2018). Improving
Lateral Flow Assay Performance Using Computational Modeling. Annu. Rev. Anal. Chem..

[38] Zhan L., Guo S. Z., Song F., Gong Y., Xu F., Boulware D. R., McAlpine M. C., Chan W. C. W., Bischof J. C. (2017). The Role
of Nanoparticle Design in Determining Analytical Performance of Lateral
Flow Immunoassays. Nano Lett..

[39] Xiao Z., Yang Y., Zhang X., Guo W. (2021). Controlling Capillary
Flow Rate on Lateral Flow Test Substrates by Tape. Micromachines.

[40] Psychogios N., Hau D. D., Peng J., Guo A. C., Mandal R., Bouatra S., Sinelnikov I., Krishnamurthy R., Eisner R., Gautam B., Young N., Xia J., Knox C., Dong E., Huang P., Hollander Z., Pedersen T. L., Smith S. R., Bamforth F., Greiner R., McManus B., Newman J. W., Goodfriend T., Wishart D. S. (2011). The Human Serum Metabolome. PLoS
One.

[41] Pérez L. M., Hooshmand B., Mangialasche F., Mecocci P., Smith A. D., Refsum H., Inzitari M., Fratiglioni L., Rizzuto D., Calderón-Larrañaga A. (2020). Glutathione
Serum Levels and Rate of Multimorbidity Development in Older Adults. J. Gerontol., Ser. A.

[42] Turell L., Radi R., Alvarez B. (2013). The Thiol
Pool in Human Plasma: The
Central Contribution of Albumin to Redox Processes. Free Radic. Biol. Med..

[43] Miller B. S., Bezinge L., Gliddon H. D., Huang D., Dold G., Gray E. R., Heaney J., Dobson P. J., Nastouli E., Morton J. J. L., McKendry R. A. (2020). Spin-Enhanced
Nanodiamond Biosensing
for Ultrasensitive Diagnostics. Nature.

[44] Azuma T., Hui Y. Y., Chen O. Y., Wang Y. L., Chang H. C. (2022). Thermometric
Lateral Flow Immunoassay with Colored Latex Beads as Reporters for
COVID-19 Testing. Sci. Rep..

[45] Szijj P. A., Gray M. A., Ribi M. K., Bahou C., Nogueira J. C. F., Bertozzi C. R., Chudasama V. (2023). Chemical Generation
of Checkpoint
Inhibitory T Cell Engagers for the Treatment of Cancer. Nat. Chem..

[46] Miller B. S., Thomas M. R., Banner M., Kim J., Chen Y., Wei Q., Tseng D. K., Göröcs Z. S., Ozcan A., Stevens M. M., McKendry R. A. (2022). Sub-Picomolar Lateral
Flow Antigen
Detection with Two-Wavelength Imaging of Composite Nanoparticles. Biosens. Bioelectron..

[47] Holstein C. A., Griffin M., Hong J., Sampson P. D. (2015). Statistical Method
for Determining and Comparing Limits of Detection of Bioassays. Anal. Chem..

