# Supplementary material for: Triggered “Capture-and-Release” Enables a High-Affinity Rebinding Strategy for Sensitivity Enhancement in Lateral Flow Assays
Source: ACS Sens. 2025 Nov 26;10(12):9432–46. doi: 10.1021/acssensors.5c02665 (PMC12751098; doi:10.1021/acssensors.5c02665)
Supplement: Supplementary file 1 [file se5c02665_si_001.pdf]

Supporting Information

Triggered 'capture-and-release' enables a high-affinity re-binding strategy for sensitivity enhancement in lateral flow assays

Chapman Ho[a,b], Clíona McMahon[c], John-Paul Ayrton[a,b], Vijay Chudasama[c]*, Michael R Thomas[a,b]*

[a] *London Centre for Nanotechnology, University College London, 17-19 Gordon Street, Bloomsbury, London, WC1H 0AH, United Kingdom*

[b] *Department of Biochemical Engineering, University College London, Gower Street, London, WC1E 6BT, United Kingdom*

[c] *Department of Chemistry, University College London, 20 Gordon Street, London, WC1H 0AJ, United Kingdom*

Supplementary Figures

Protein modification

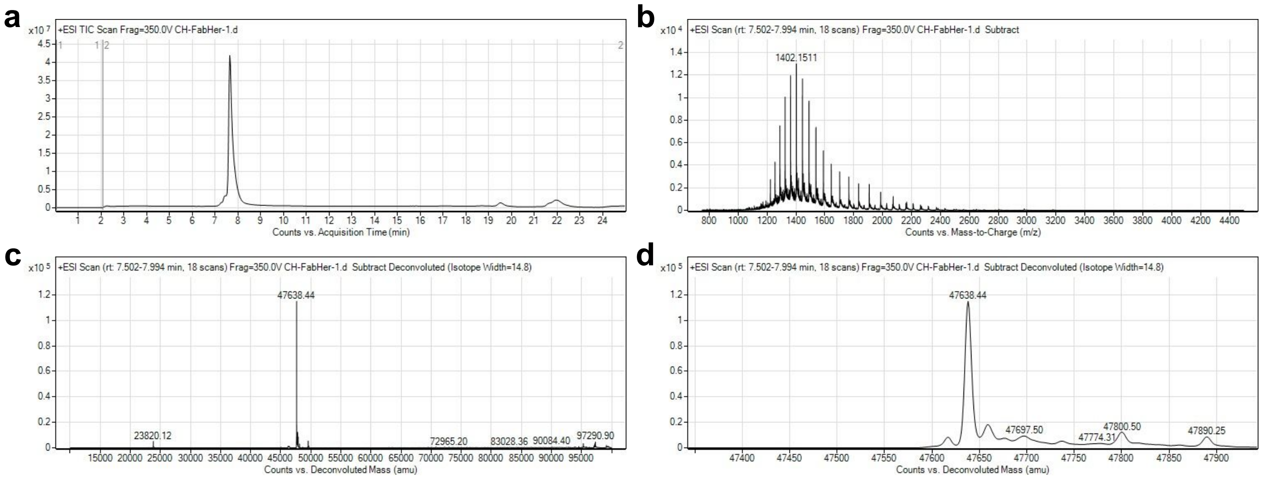

Figure S1: LC-MS spectra for native Fab$_{HER}$ comprising of the **a** total ion chromatogram; **b** non-deconvoluted ion series; **c** deconvoluted ion series mass spectrum; **d** mass spectrum region of interest; observed mass of 47638.4 corresponds to native Fab$_{HER}$.

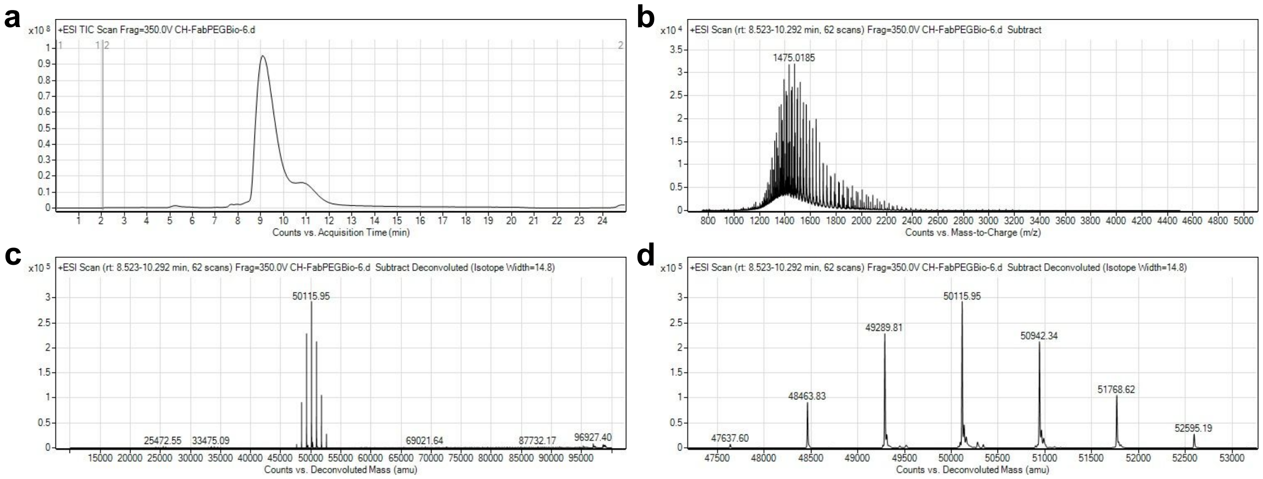

Figure S2: LC-MS spectra for Fab$_{HER}$-PEG$_{12}$-biotin conjugates **(1)** observing the addition of expected linker mass (825.6 g/mol) to the native Fab$_{HER}$ (47638.4 g/mol), following protein modification with NHS-PEG$_{12}$-biotin and purification. **a** total ion chromatogram; **b** non-deconvoluted ion series; **c** deconvoluted ion series mass spectrum; **d** mass spectrum

region of interest; observed mass of 47637.6 corresponds to native Fab$_{HER}$, 48463.8 corresponds to 1 linker addition to Fab$_{HER}$, 49289.8 corresponds to 2 linker additions to Fab$_{HER}$, 50115.5 corresponds to 3 linker additions to Fab$_{HER}$, 50942.3 corresponds to 4 linker additions to Fab$_{HER}$, 51768.6 corresponds to 5 linker additions to Fab$_{HER}$, 52595.2 corresponds to 6 linker additions to Fab$_{HER}$.

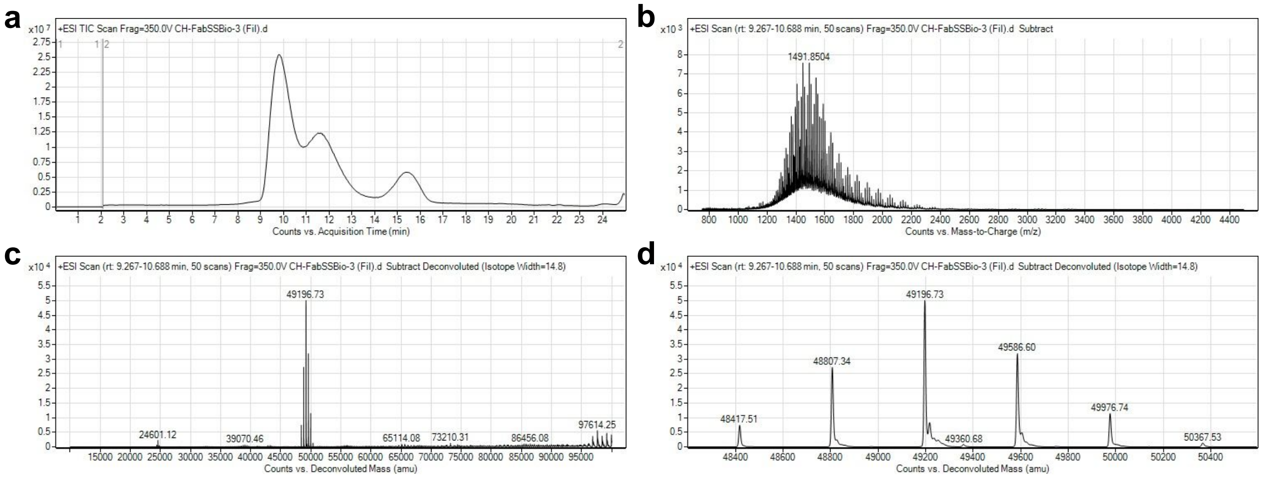

Figure S3: LC-MS spectra for Fab$_{HER}$-SS-biotin conjugates **(2)** observing the addition of expected linker mass (389.6 g/mol) to the native Fab$_{HER}$ (47638.4 g/mol), following protein modification with sulfo-NHS-SS-biotin and purification. **a** total ion chromatogram; **b** non-deconvoluted ion series; **c** deconvoluted ion series mass spectrum; **d** mass spectrum region of interest; observed mass of 48417.5 corresponds to 2 linker addition to Fab$_{HER}$, 48807.3 corresponds to 3 linker additions to Fab$_{HER}$, 49196.7 corresponds to 4 linker additions to Fab$_{HER}$, 49586.6 corresponds to 5 linker additions to Fab$_{HER}$, 49976.7 corresponds to 6 linker additions to Fab$_{HER}$, 50367.5 corresponds to 7 linker additions to Fab$_{HER}$.

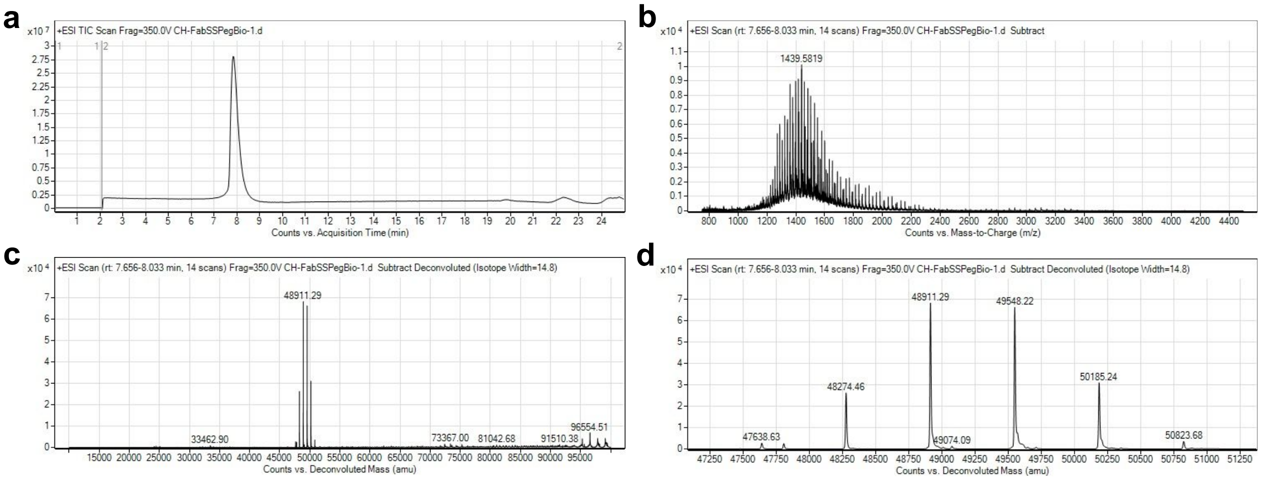

Figure S4: LC-MS spectra for Fab_{HER}-SS-PEG_4-biotin conjugates **(3)** observing the addition of expected linker mass (636.9 g/mol) to the native Fab_{HER} (47638.4 g/mol), following protein modification with NHS-SS-PEG_4-biotin and purification. **a** total ion chromatogram; **b** non-deconvoluted ion series; **c** deconvoluted ion series mass spectrum; **d** mass spectrum region of interest; observed mass of 47638.6 corresponds to native Fab_{HER}, 48274.5 corresponds to 1 linker addition to Fab_{HER}, 48911.3 corresponds to 2 linker additions to Fab_{HER}, 49548.2 corresponds to 3 linker additions to Fab_{HER}, 50185.2 corresponds to 4 linker additions to Fab_{HER}, 50823.7 corresponds to 5 linker additions to Fab_{HER}.

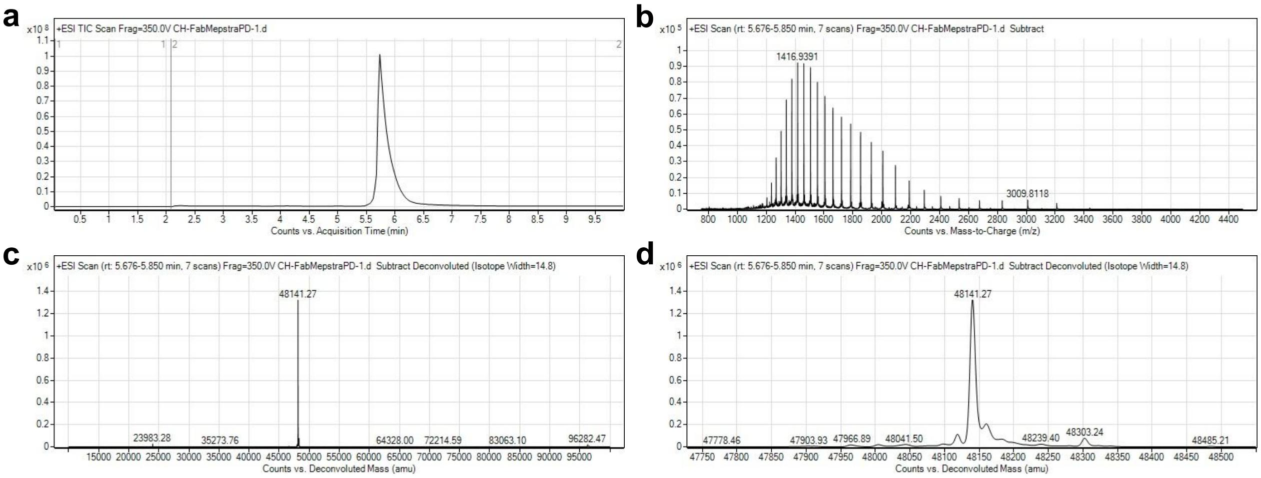

Figure S5: LC-MS spectra for Fab_{HER}-PD-BCN(endo) conjugates observing the addition of expected mass (502.6 g/mol) to the native Fab_{HER} (47638.4 g/mol), following disulfide

reduction, PD modification and purification. **a** total ion chromatogram; **b** non-deconvoluted ion series; **c** deconvoluted ion series mass spectrum; **d** mass spectrum region of interest; observed mass of 48141.3 corresponds to the addition of PD-BCN(endo) to Fab$_{HER}$.

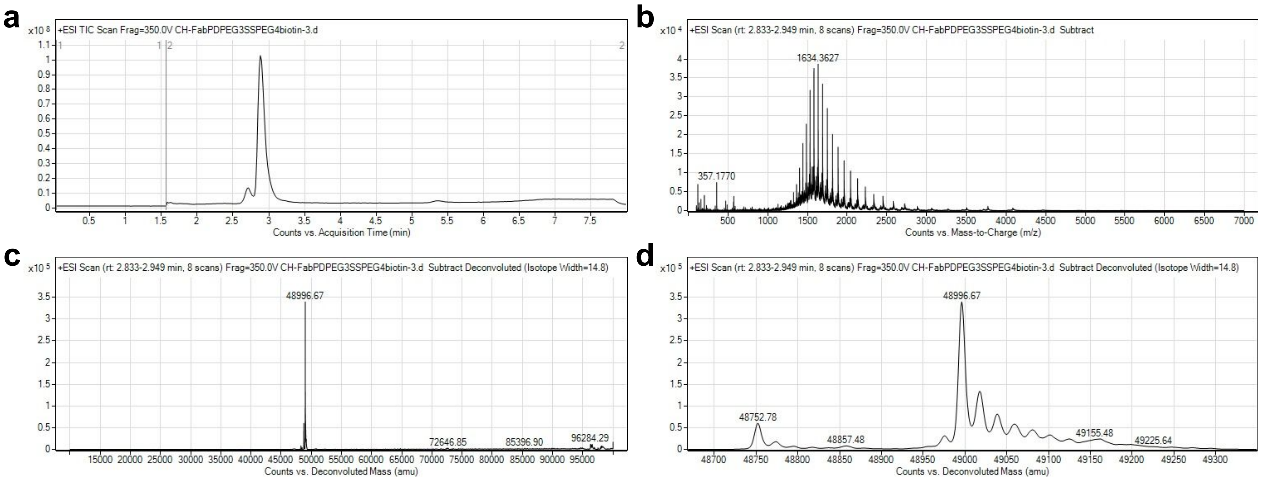

Figure S6: LC-MS spectra for Fab$_{HER}$-PD-PEG3-SS-PEG4-biotin conjugates **(4)** observing the addition of expected linker mass (855.1 g/mol) to the Fab(PD)-BCN(endo) conjugate (48141.3 g/mol), following strain-promoted azide-alkyne cycloaddition (SPAAC) ‘click’ reaction and purification. **a** total ion chromatogram; **b** non-deconvoluted ion species; **c** deconvoluted ion species mass spectrum; **d** mass spectrum region of interest; observed mass of 48996.7 corresponds to the addition of linker to Fab$_{HER}$.

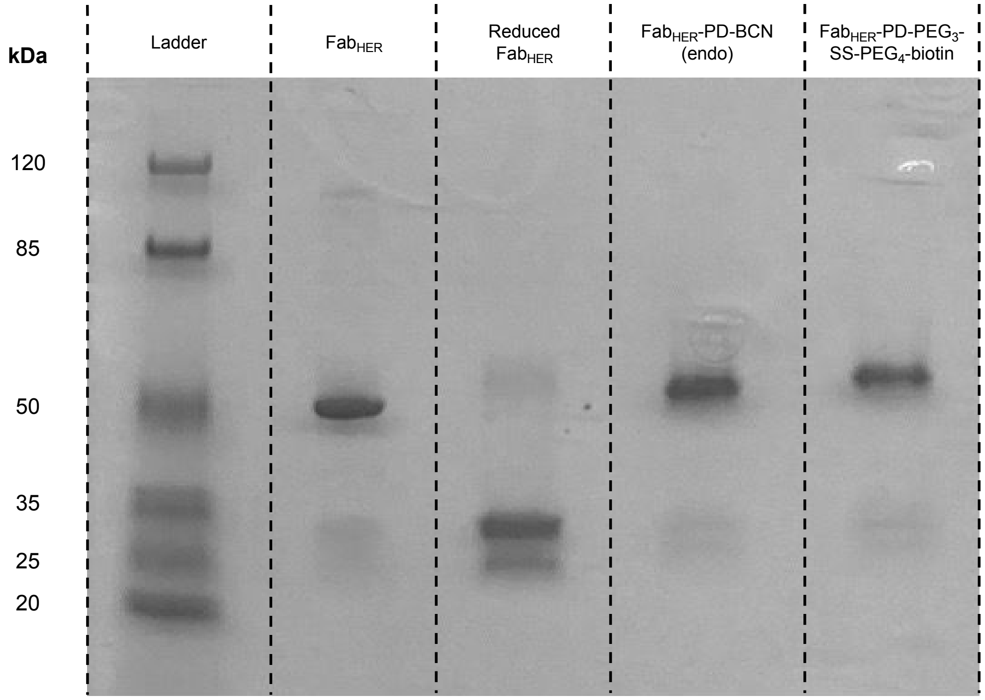

Figure S7: Non-reducing SDS-PAGE for native Fab_{HER}, Fab_{HER} heavy and light chain following disulfide reduction, the rebridged Fab_{HER}-PD-BCN(endo) conjugate, and the Fab_{HER}-PD-PEG_3-SS-PEG_4-biotin **(4)** conjugate.

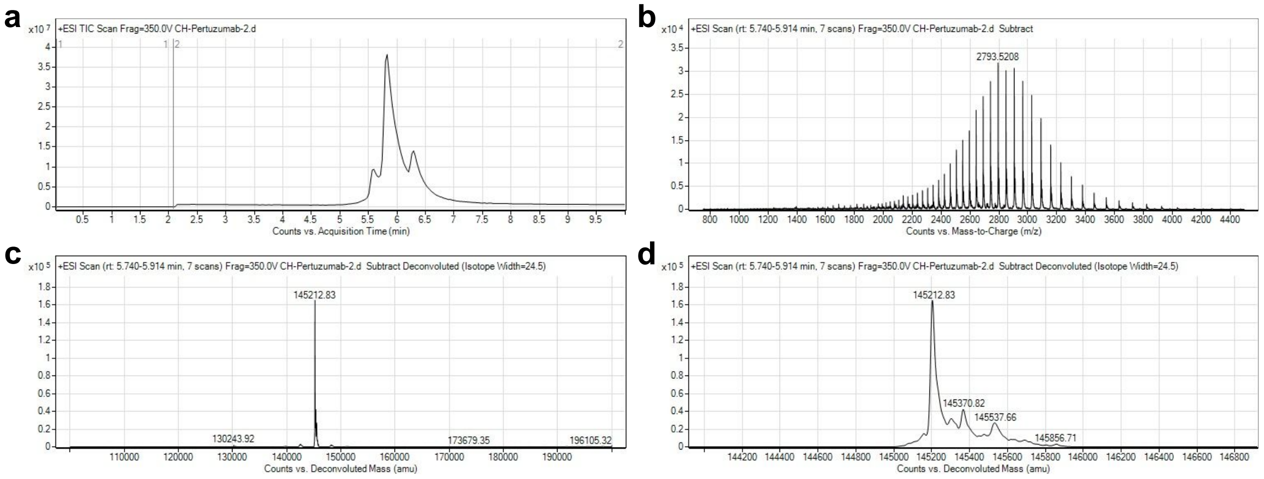

Figure S8: LC-MS spectra for native anti-HER2 mAb, Pertuzumab, comprising of the **a** total ion chromatogram; **b** non-deconvoluted ion series; **c** deconvoluted ion series mass spectrum; **d** mass spectrum region of interest; observed mass of 145212.8 corresponds to native Pertuzumab.

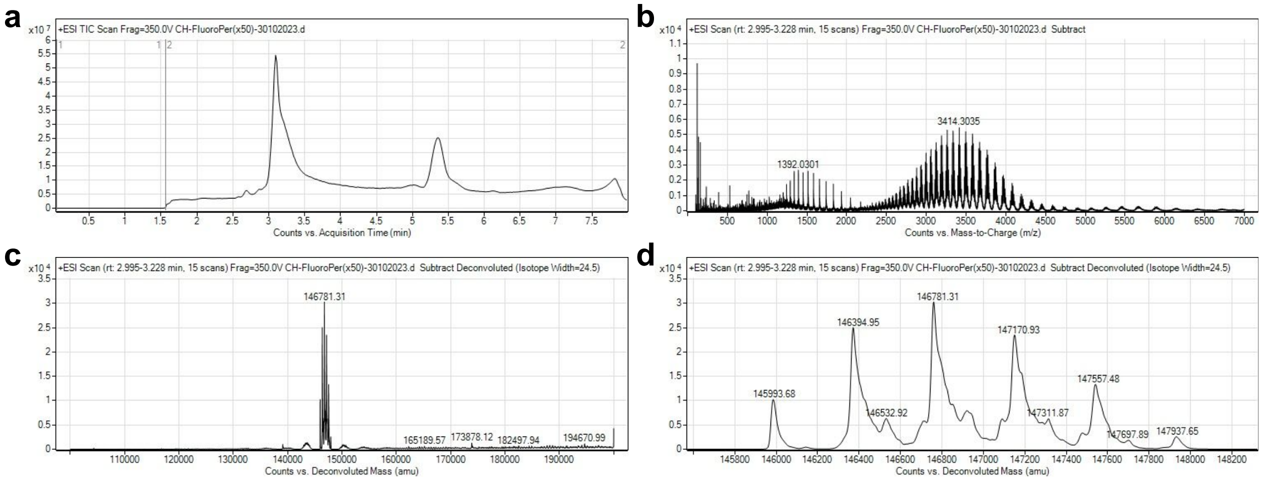

Figure S9: LC-MS spectra for fluorescein-labelled Pertuzumab conjugates observing the addition of expected label mass (388.4 g/mol) to native Pertuzumab (145212.8 g/mol), following protein modification with FITC and purification. **a** total ion chromatogram; **b** non-deconvoluted ion series; **c** deconvoluted ion series mass spectrum; **d** mass spectrum region of interest; observed mass of 145993.7 corresponds to 2 fluorescein additions to Pertuzumab, 146395.0 corresponds to 3 fluorescein additions to Pertuzumab, 146781.3 corresponds to 4 fluorescein additions to Pertuzumab, 147170.9 corresponds to 5

fluorescein additions to Pertuzumab, 147557.5 corresponds to 6 fluorescein additions to Pertuzumab, 147937.7 corresponds to 7 fluorescein additions to Pertuzumab.

Antibody-nanoparticle conjugates

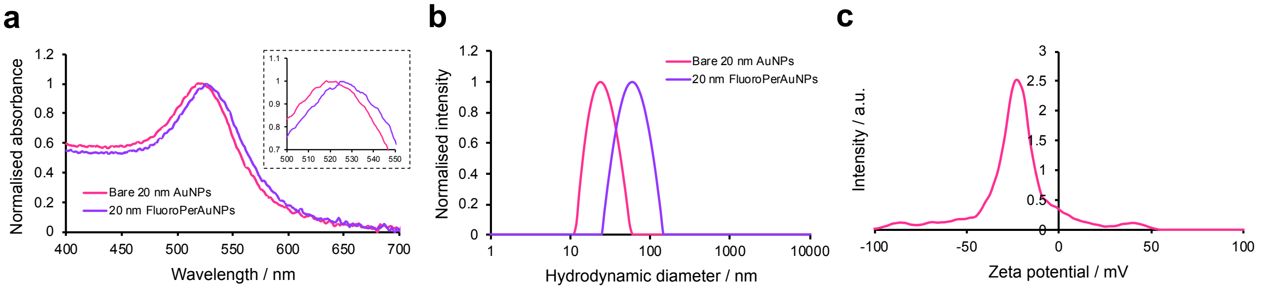

Figure S10: a Normalised UV-Vis absorbance spectra for bare 20 nm AuNPs and 20 nm FluoroPerAuNP conjugates, following BSA blocking; region of interest indicated by dashed box which compares peak maxima between bare (523 nm) and BSA blocked 20 nm FluoroPerAuNP conjugates (526 nm). **b** Particle sizing, by hydrodynamic diameter peak maxima, of bare 20 nm AuNPs (24.9 nm) and BSA blocked 20 nm FluoroPerAuNP conjugates (60.7 nm) by DLS; 20 nm FluoroPerAuNP conjugates were re-suspend in 0.1% v/v Tween20 in borate buffer (pH 8.0, 100 mM) for particle size measurements. **c** Zeta potential of BSA blocked 20 nm FluoroPerAuNP conjugates measured by ELS; peak maximum corresponds to -22.5 mV.

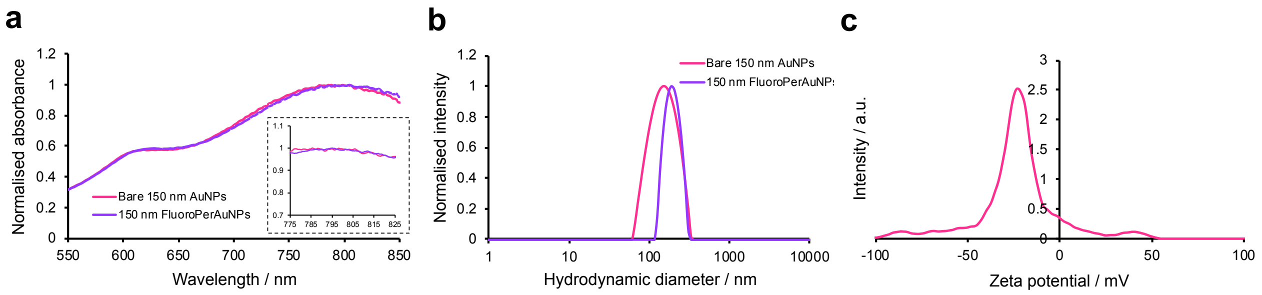

Figure S11: a Normalised UV-Vis absorbance spectra for bare 150 nm AuNPs and 150 nm FluoroPerAuNP conjugates, following BSA blocking; region of interest indicated by dashed box which compares peak maxima between bare (798 nm) and BSA blocked 150 nm FluoroPerAuNP conjugates (804 nm). **b** Particle sizing, by hydrodynamic diameter peak maxima, of bare 150 nm AuNPs (148 nm) and BSA blocked 150 nm FluoroPerAuNP

conjugates (189 nm) by DLS. **c** Zeta potential of BSA blocked 150 nm FluoroPerAuNP conjugates measured by ELS; peak maximum corresponds to -32.0 mV.

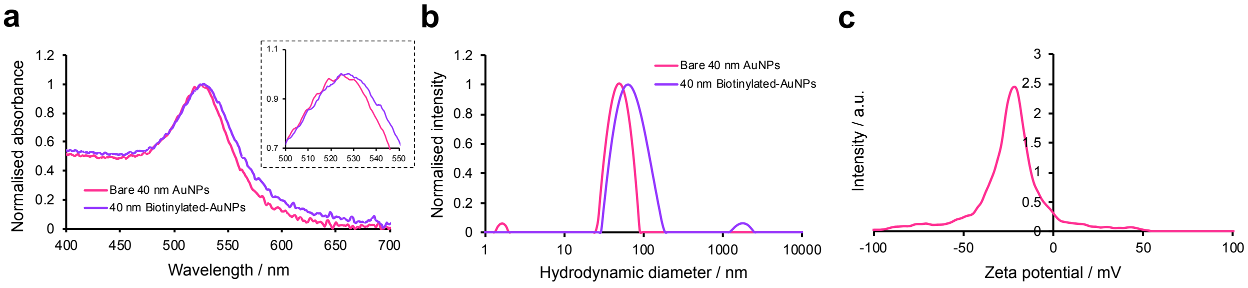

Figure S12: a Normalised UV-Vis absorbance spectra for bare 40 nm AuNPs and 40 nm biotinylated-AuNPs, following BSA blocking; region of interest indicated by dashed box which compares peak maxima between bare (525 nm) and BSA blocked 40 nm biotinylated-AuNPs (528 nm). **b** Particle sizing, by hydrodynamic diameter peak maxima, of bare 40 nm AuNPs (51.7 nm) and BSA blocked 40 nm biotinylated-AuNPs (65.9 nm) by DLS. **c** Zeta potential of BSA blocked 40 nm biotinylated-AuNPs measured by ELS; peak maximum corresponds to -21.9 mV.

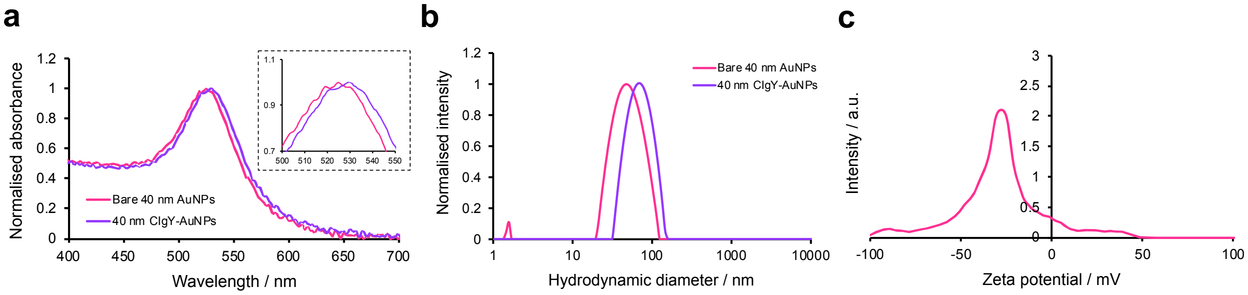

Figure S13: a Normalised UV-Vis absorbance spectra for bare 40 nm AuNPs and 40 nm CIgY-AuNP conjugates; region of interest indicated by dashed box which compares peak maxima between bare (525 nm) and 40 nm CIgY-AuNP conjugates (530 nm). **b** Particle sizing, by hydrodynamic diameter peak maxima, of bare 40 nm AuNPs (47.6 nm) and 40 nm CIgY-AuNP conjugates (71.4 nm) by DLS. **c** Zeta potential of BSA blocked 40 nm CIgY-AuNP conjugates measured by ELS; peak maximum corresponds to -27.9 mV.

Organic synthesis

Synthesis of azido-PEG₃-SS-PEG₄-biotin

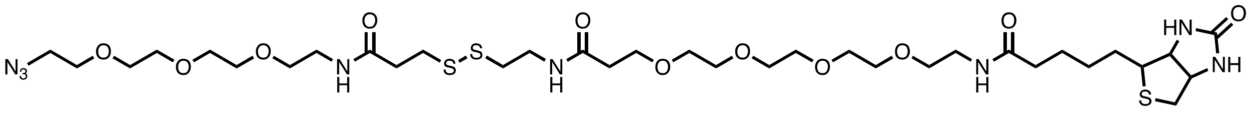

The following procedure was adapted from work by Szychowski *et al.*.[1] To NHS-SS-PEG$_4$-biotin (16.5 mg, 21.9 μmol, 1 eq.), DCM (1.65 mL), azido-PEG$_3$-amine (5.56 μL, 28.0 μmol, 1.28 eq.; Scientific Laboratory Supplies) and NEt_3 (3.9 μL, 28.0 μmol, 1.28 eq.) were added and the reaction mixture was stirred (21 ˚C, 2 h, 600 rpm). The mixture was dried under reduced pressure prior to re-suspension in DCM (2 mL). The crude product was purified by flash column chromatography (10-15 % gradient of MeOH/DCM), and the appropriate fractions were combined and concentrated under reduced pressure. Following this, the residue was suspended in DCM (2 mL) prior to the addition of citric acid (1 mL). The suspension was washed with water (3 × 1 mL) and the organic layer was then evaporated under reduced pressure to yield *N*-(19-azido-7-oxo-11,14,17-trioxa-3,4-dithia-8-azanonadecyl)-1-(5-(2-oxohexahydro-1*H*-thieno[3,4-*d*]imidazol-4-yl)pentanamido)-3,6,9,12-tetraoxapentadecan-15-amide (4.0 mg, 4.7 μmol, 21%) as a light brown oil. ^{1}H NMR (400 MHz, $CDCl_3$) δ 7.24 (d, *J* = 5.8 Hz, 1H, C=ON<u>H</u>), 7.07 (t, *J* = 5.2 Hz, 1H, C=ON<u>H</u>), 6.97 (t, *J* = 5.6 Hz, 1H C=ON<u>H</u>), 6.54 (s, 1H C=ON<u>H</u>), 5.80 (s, 1H, C=ON<u>H</u>), 4.50 (dd, *J* = 7.9, 4.9 Hz, 1H, biotin-S-CH-C<u>H</u>), 4.31 (dd, *J* = 8.0, 4.6 Hz, 1H, biotin-S-CH-C<u>H</u>), 3.72 (t, *J* = 5.9 Hz, 2H, biotin-S-C<u>H</u>), 3.69-3.35 (m, 36H, CH_2), 3.13 (q, *J* = 7.0 Hz, 1H, biotin-S-C<u>H</u>), 2.96 (t, *J* = 7.2 Hz, 2H, CH_2), 2.80 (q, *J* = 5.5 Hz, 2H, CH_2), 2.59 (t, *J* = 7.1 Hz, 2H, CH_2), 2.48 (t, *J* = 6.0 Hz, 2H, CH_2), 2.22 (t, *J* = 7.6 Hz, 2H, CH_2), 1.77-1.56 (m, 4H, CH_2), 1.41 (t, *J* = 7.5 Hz, 2H, CH_2); ^{13}C NMR (100 MHz, $CDCl_3$) δ 25.7, 28.2, 28.3, 34.3, 35.9, 36.2, 36.9, 38.0, 38.4, 39.3, 39.4, 40.6, 50.8, 55.6, 60.4, 62.0, 67.3, 69.8, 70.0, 70.1, 70.1, 70.2, 70.3, 70.4, 70.5, 70.6, 70.6, 70.7, 70.8, 164.4 (C=O), 171.4 (C=O), 172.0 (C=O), 173.9 (C=O); IR (thin film) 3294, 2882, 2118, 1647, 1559, 1471, 1353, 1279, 1119 cm^{-1}; LRMS (ES+) 428.4 (100, $[M+2H]^{2+}$), 855.6 (50, $[M+H]^+$); HRMS (ESI) calculated for $C_{36}H_{63}O_{15}N_4{}^{32}S_2$ $[M]^+$ 855.3726, observed 855.3763.

Mass spectrometry observed linker cleavage

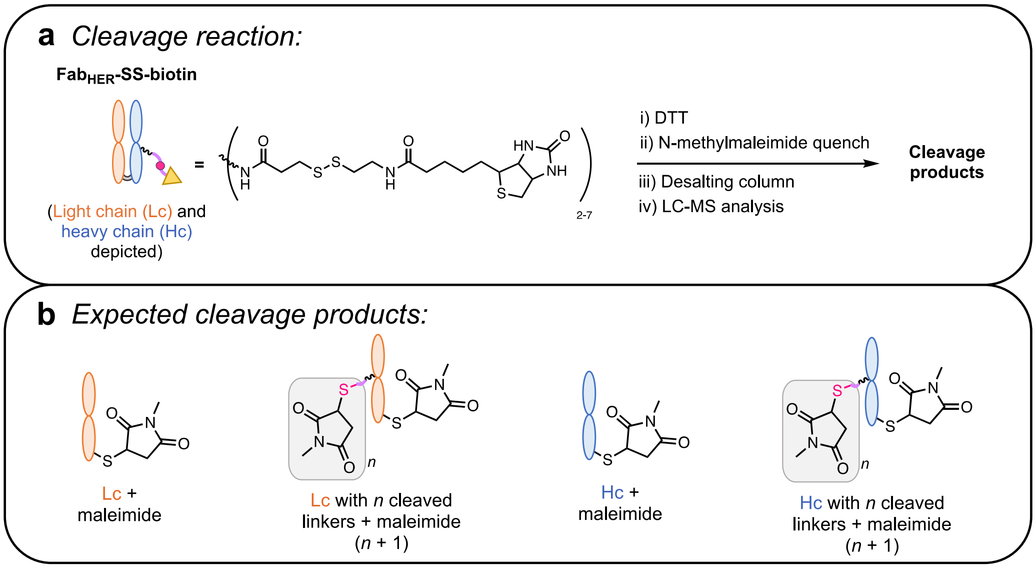

Figure S14: a Proposed linker cleavage workflow for Fab$_{HER}$-SS-biotin conjugate **(2)** using DTT prior to thiol quenching, purification and LC-MS characterisation. **b** The expected products following DTT incubation comprising of maleimide-capped light and heavy chains, and their cleaved linker conjugates.

	Expected mass / Da	Structure
Fab_{HER} fragment	47638.4	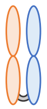
Light chain (Lc)	23439.9	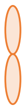
Heavy chain (Hc)	24200.7	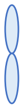
Whole linker (-SS-biotin)	389.6	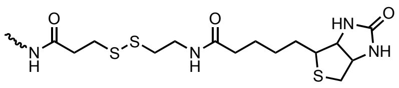
Cleaved linker	89.1	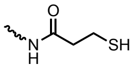
N-methylmaleimide	111.1	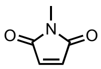

Table S1: Individual expected masses and structure for expected components in mass spectrometry observed linker cleavage studies of Fab_{HER}-SS-biotin conjugate **(2)**. Native light and heavy chain masses correspond to values in Fab_{HER} reduction studies conducted by Forte *et al.*.[2]

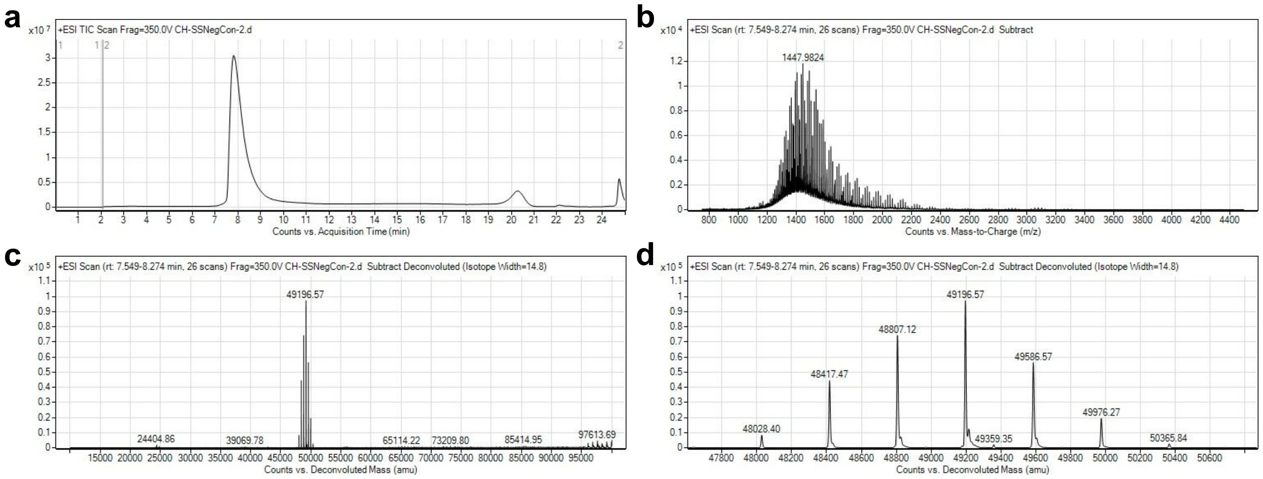

Figure S15: LC-MS spectra for Fab_{HER}-SS-biotin conjugate **(2)** incubated with DTT (0 mM, 10 min), following reaction quenching and desalting steps. **a** total ion chromatogram;

b non-deconvoluted ion series; **c** deconvoluted ion series mass spectrum; **d** mass spectrum region of interest; mass spectrum region of interest; observed mass of 48028.4 corresponds to Fab with 1 linker, 48417.5 corresponds to Fab with 2 linkers, 48807.1 corresponds to Fab with 3 linkers, 49196.6 corresponds to Fab with 4 linkers, 49586.6 corresponds to Fab with 5 linkers, 49976.3 corresponds to Fab with 6 linkers, 50365.8 corresponds to Fab with 7 linkers.

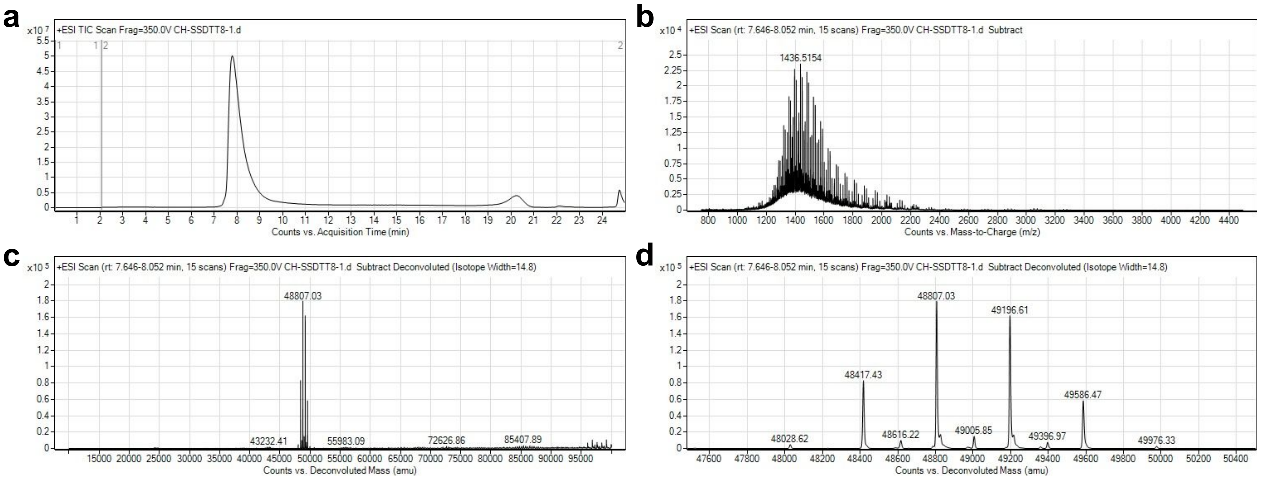

Figure S16: LC-MS spectra for Fab_{HER}-SS-biotin conjugate **(2)** incubated with DTT (0.25 mM, 10 min), following reaction quenching and desalting steps. **a** total ion chromatogram; **b** non-deconvoluted ion series; **c** deconvoluted ion series mass spectrum; **d** mass spectrum region of interest; mass spectrum region of interest; observed mass of 48028.6 corresponds to Fab_{HER} with 1 linker, 48417.4 corresponds to Fab_{HER} with 2 linkers, 48807.0 corresponds to Fab_{HER} with 3 linkers, 49196.6 corresponds to Fab_{HER} with 4 linkers, 49586.5 corresponds to Fab_{HER} with 5 linkers, 49976.3 corresponds to Fab_{HER} with 6 linkers.

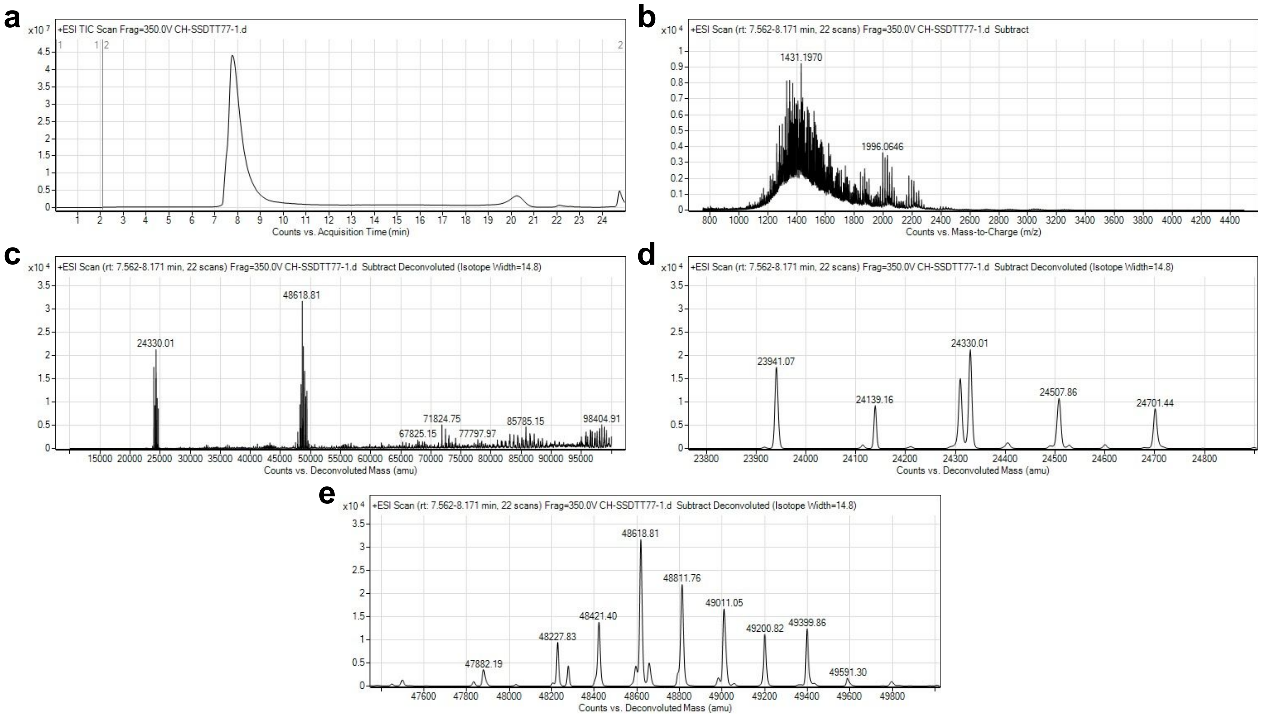

Figure S17: LC-MS spectra for Fab_{HER}-SS-biotin conjugate **(2)** incubated with DTT (2.5 mM, 10 min), following reaction quenching and desalting steps. **a** total ion chromatogram; **b** non-deconvoluted ion series; **c** deconvoluted ion series mass spectrum; **d** mass spectrum region of interest (~ 24 kDa); observed mass of 23941.1 corresponds to Lc with 1 linker and 1 N-methylmaleimide addition, 24139.2 corresponds to Lc with 1 linker and 1 cleaved linker and 2 N-methylmaleimide additions, 24330.0 corresponds to Lc with 2 linkers and 1 N-methylmaleimide addition, 24507.9 corresponds to Hc with 1 cleaved linker and 2 N-methylmaleimide additions, 24701.4 corresponds to Hc with 1 linker and 1 N-methylmaleimide addition; **e** mass spectrum region of interest (~ 48 kDa); observed mass of 48227.8 corresponds to Fab_{HER} with 1 linker and 1 cleaved linker and 1 N-methylmaleimide addition, 48421.4 corresponds to Fab_{HER} with 2 linker, 48618.8 corresponds to Fab_{HER} with 2 linkers and 1 cleaved linker and 1 N-methylmaleimide, 48811.8 corresponds to Fab_{HER} with 3 linkers, 49011.1 corresponds to Fab_{HER} with 3 linkers and 1 cleaved linker and 1 N-methylmaleimide addition, 49200.8 corresponds to Fab_{HER} with 4 linkers, 49399.9 corresponds to Fab_{HER} with 4 linkers and 1 cleaved linker and 1 N-methylmaleimide, 49591.3 corresponds to Fab_{HER} with 5 linkers.

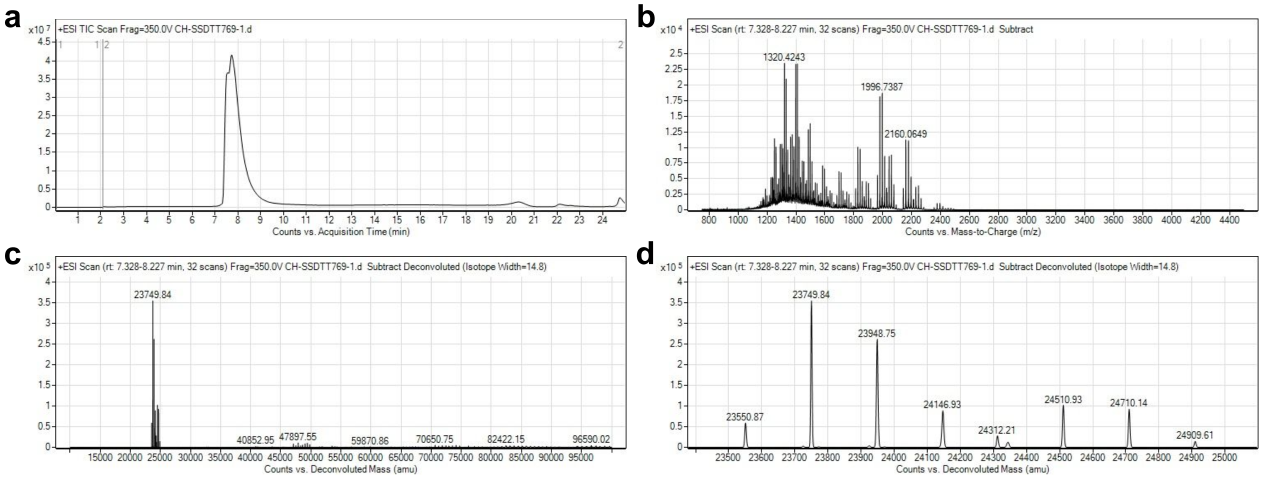

Figure S18: LC-MS spectra for Fab_{HER}-SS-biotin conjugate **(2)** incubated with DTT (25 mM, 10 min), following reaction quenching and desalting steps. **a** UV chromatogram; **b** non-deconvoluted ion series; **c** deconvoluted ion series mass spectrum; **d** mass spectrum region of interest; observed mass of 23550.87 corresponds to Lc with 1 N-methylmaleimide addition, 23749.8 corresponds to Lc with 1 cleaved linker and 2 N-methylmaleimide additions, 23948.8 corresponds to Lc with 2 cleaved linkers and 3 N-methylmaleimide additions, 24146.9 corresponds to Lc with 3 cleaved linkers and 4 N-methylmaleimide additions, 24312.2 corresponds to Hc with 1 N-methylmaleimide addition, 24510.9 corresponds to Hc with 1 cleaved linker and 2 N-methylmaleimide additions, 24710.1 corresponds to Hc with 2 cleaved linkers and 3 N-methylmaleimide additions, 24909.6 corresponds to Hc with 3 cleaved linkers and 4 N-methylmaleimide additions.

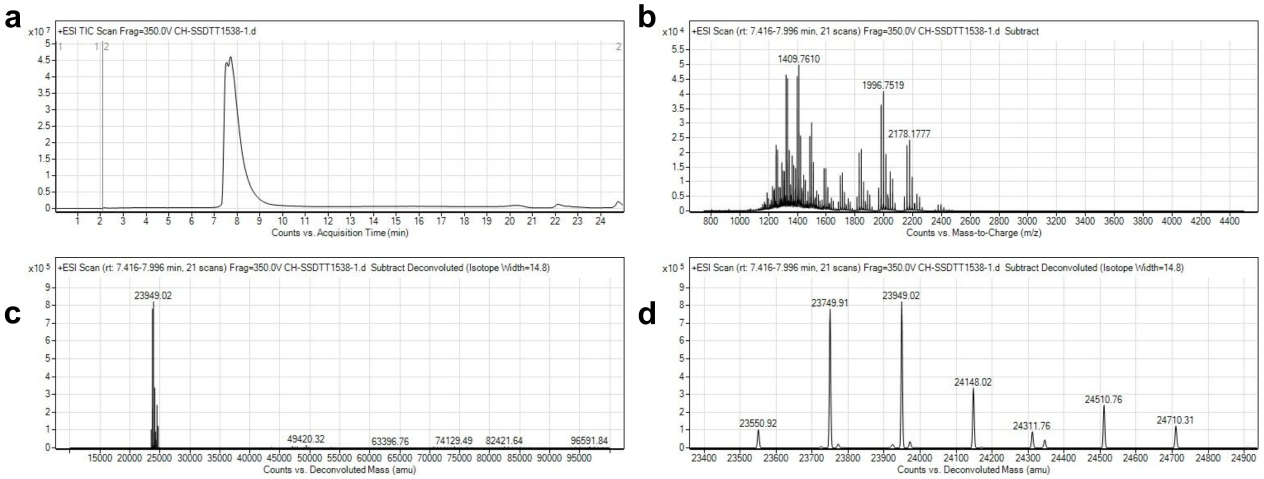

Figure S19: LC-MS spectra for Fab_{HER}-SS-biotin conjugate **(2)** incubated with DTT (50 mM, 10 min), following reaction quenching and desalting steps. **a** UV chromatogram; **b** non-deconvoluted ion series; **c** deconvoluted ion series mass spectrum; **d** mass spectrum region of interest; observed mass of 23550.92 corresponds to Lc with 1 N-methylmaleimide addition, 23749.9 corresponds to Lc with 1 cleaved linker and 2 N-methylmaleimide additions, 23949.0 corresponds to Lc with 2 cleaved linkers and 3 N-methylmaleimide additions, 24148.0 corresponds to Lc 3 cleaved linkers and 4 N-methylmaleimide additions, 24311.8 corresponds to Hc with 1 N-methylmaleimide addition, 24510.8 corresponds to Hc with 1 cleaved linker and 2 N-methylmaleimide additions, 24710.3 corresponds to Hc with 2 cleaved linkers and 3 N-methylmaleimide additions.

Lateral flow immunoassay

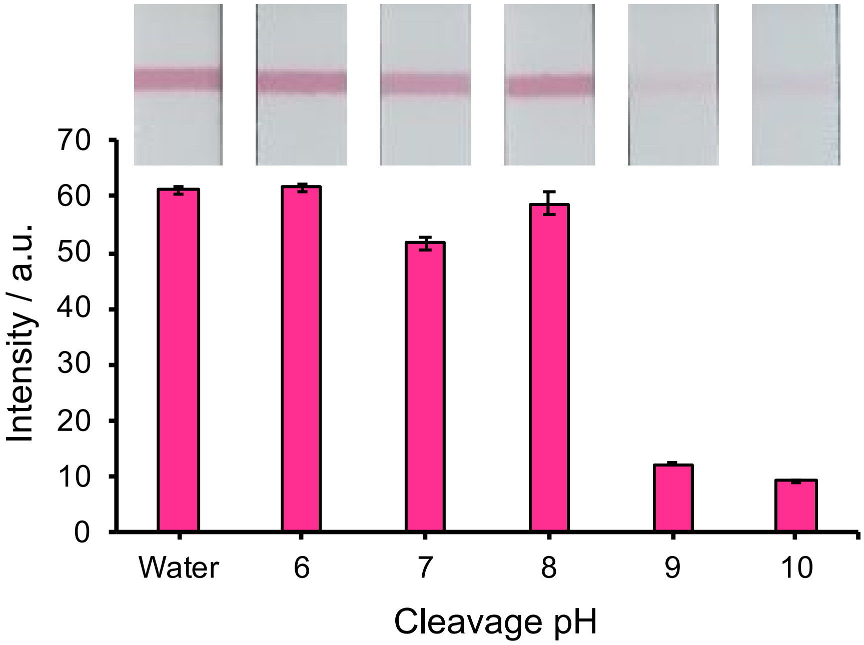

Figure S20: The mean intensity (N=2) of cleaved signal from LFA printed with PSA (1 mg/mL) test lines, following the capture of sandwich immunocomplexes – comprising HER2, Fab_{HER}-PD-PEG_3-SS-PEG_4-biotin **(4)**, and 40 nm FluoroPerAuNP conjugates – using DTT (50 mM) in different cleavage buffer pH conditions.

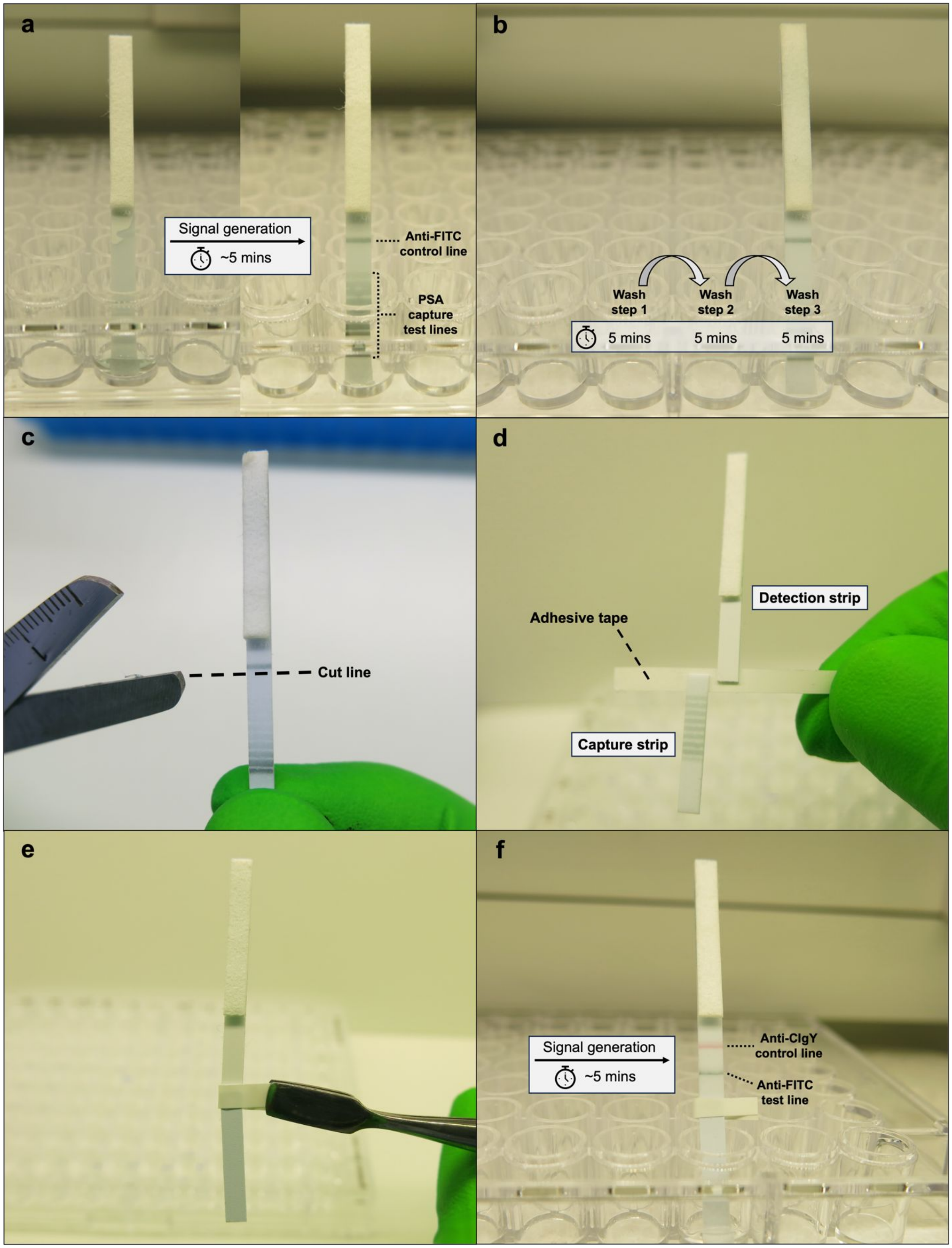
a
Signal generation
~5 mins
Anti-FITC
control line
PSA
capture
test lines
b
Wash
step 1
Wash
step 2
Wash
step 3
5 mins
5 mins
5 mins
c
Cut line
d
Detection strip
Adhesive tape
Capture strip
e
f
Signal generation
~5 mins
Anti-CIgY
control line
Anti-FITC
test line

Figure S21: A typical assembly of PSA-printed capture strips and anti-fluorescein pAb printed detection strips into an AmpliFold format. **a** The addition of the capture strip – into wells containing an assay sample mixture of HER2, Fab_{HER}-PD-PEG_3-SS-PEG_4-biotin **(4)**, and 150 nm FluoroPerAuNP conjugates – and the generation of signal (after ~5 mins) resulting from the capture of sandwich immunocomplexes; **b** the capture strip following three sequential wash steps (each 5 minutes) used to remove membrane fouling FluoroPerAuNP conjugates in the capture strip **c** manual cutting of the capture strip, shown below the control line in this example, resulting in the removal of the absorbent pad; **d** the arrangement of the capture strip and the detection strip onto adhesive tape; **e** assembled AmpliFold test with strip-to-strip contact following the folding over of the adhesive tape; **f** signal formation on the detection strip following the wicking of cleavage reagent and re-binding of particles; 40 nm CIgY-AuNP conjugates are simultaneously delivered in the cleavage reagent mixture and are shown binding the anti-CIgY control line.

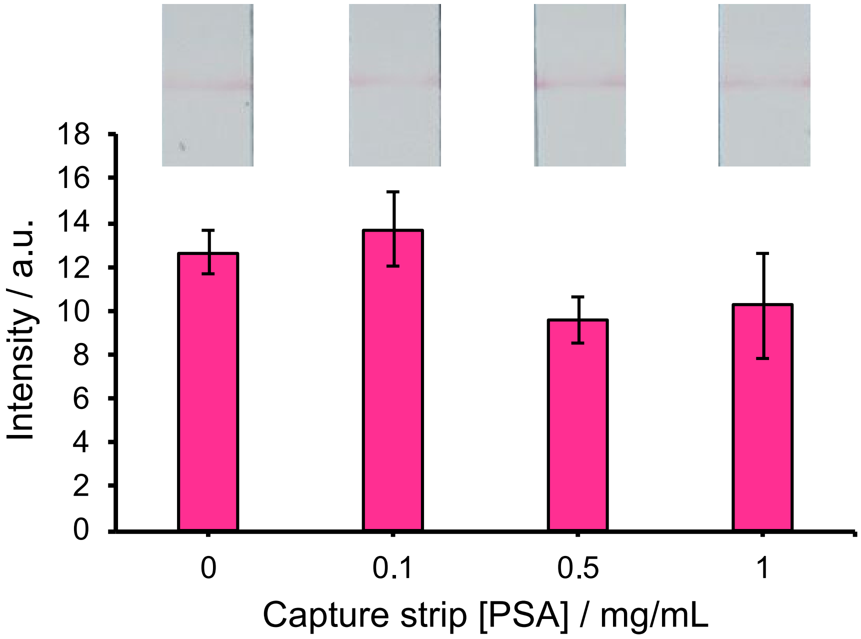

Figure S22: The mean signal intensity (N=3) of detection anti-fluorescein (1 mg/mL) test lines for HER2-negative (0 ng/mL) AmpliFold tests following elution from capture strips printed with 6 test lines of different PSA concentrations. False positive signal resulting from elution of ‘blank’ 0 mg/mL PSA capture strips indicates undesirable membrane fouling of capture strips by non-specifically bound 40 nm FluoroPerAuNP conjugates.

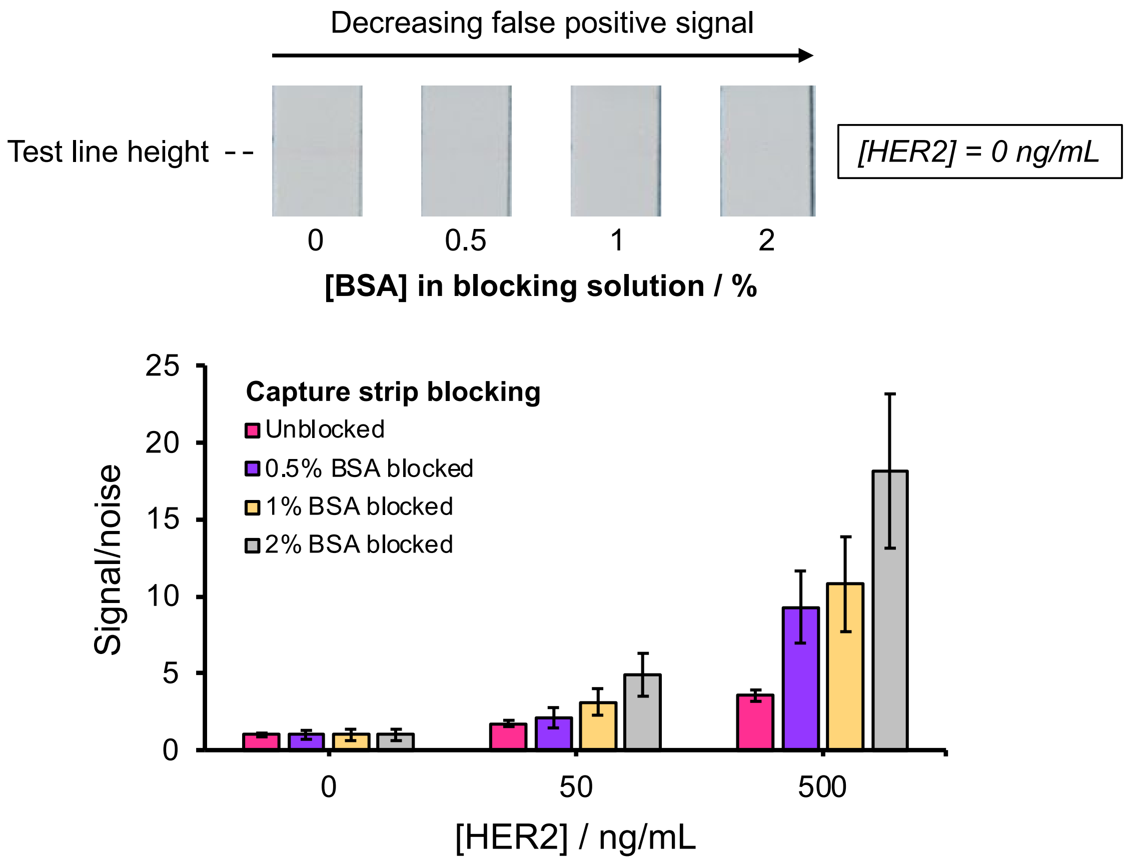

Figure S23: The mean signal-to-noise (N=3) of detection strip anti-FITC (1 mg/mL) test lines in AmpliFold tests following the cleavage and re-binding of complexes from capture strips where membranes were blocked with differing percentages of BSA. Photographic examples of HER2-negative (0 ng/mL) test lines are provided to demonstrate the extent of false positive signal observed, and the effect of increasing BSA % w/v in blocking solutions used for membrane blocking of capture test strips.

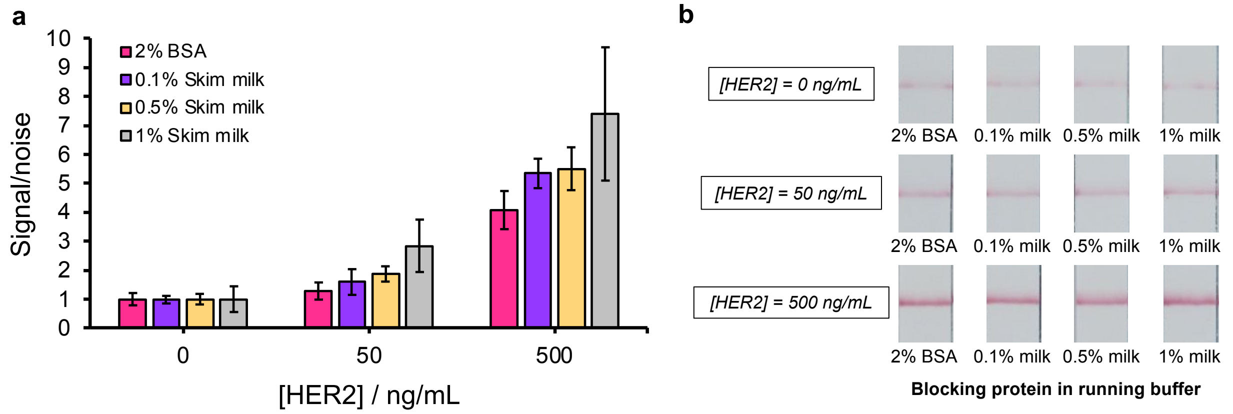

Figure S24: a The mean signal-to-noise (N=3) of detection strip anti-fluorescein (1 mg/mL) test lines in AmpliFold tests following the cleavage and re-binding of complexes from capture strips where the assay running buffer was composed of either BSA (2% w/v) or skimmed milk blocking proteins (0.1, 0.5, or 1% w/v). **b** Photographic examples of test strips are provided to demonstrate the extent of false positive and specific signal observed. Photos demonstrate that additions of skimmed milk did not drastically reduce false positive signal, when compared to BSA-containing running buffer alone. For experiments using a 1% w/v skimmed milk running buffer, a greater signal-to-noise was the result of improved signal output for HER2 positive (50 and 500 ng/mL) AmpliFold tests.

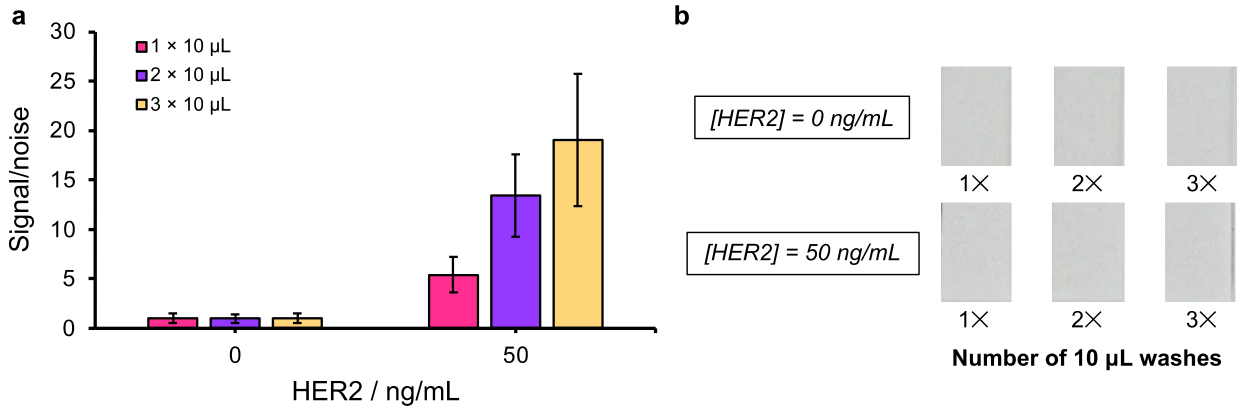

Figure S25: a The mean signal-to-noise (N=3) of detection strip anti-fluorescein (1 mg/mL) test lines in AmpliFold tests where the number of 10 µL (of 0.5% v/v IGEPAL solution) wash steps (each with 5 minutes of wicking) was optimised to remove

membrane fouling FluoroPerAuNP conjugates from capture strips, prior to the cleavage step. Signal-to-noise improves as the number of 10 μL wash steps is increased due to a reduction in false positive signal with each wash. **b** Photographic examples of test strips are provided to demonstrate the extent of false positive and specific signal observed, and the overall effect of employing a greater number of wash steps.

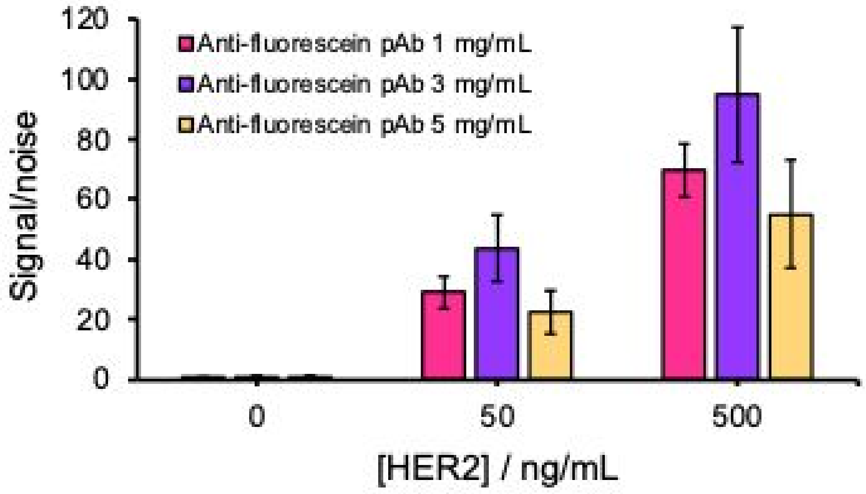

Figure S26: The mean signal-to-noise (N=3) of detection strip anti-fluorescein test lines in AmpliFold tests where the concentration of anti-fluorescein pAb printed test lines, printed on detection strips, was varied in AmpliFold 'capture-and-release' experiments.

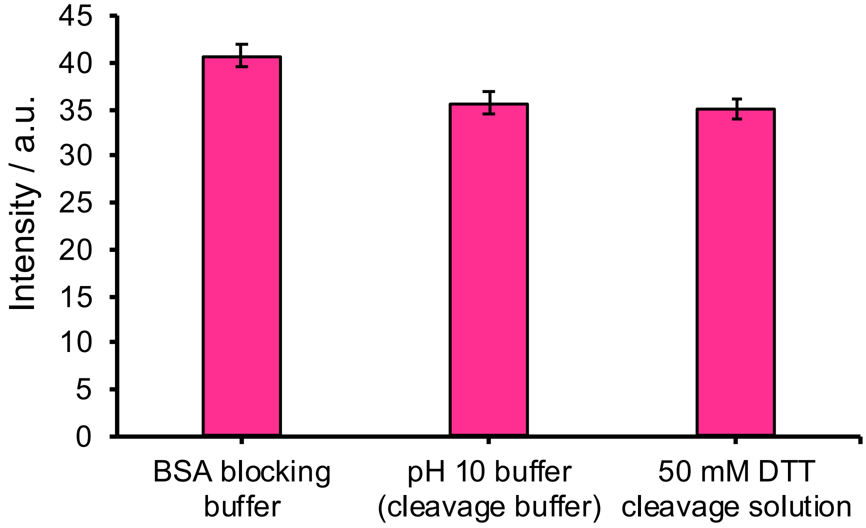

Figure S27: The mean intensities (N=3) of LFA test strips printed with anti-CIgY test lines following the flowing up of 40 nm CIgY-AuNP conjugates (OD 0.1). 40 nm CIgY-AuNPs strip were prepared in microwells with either BSA blocking buffer, cleavage buffer (pH 10, no DTT), or cleavage solution containing 50 mM DTT.

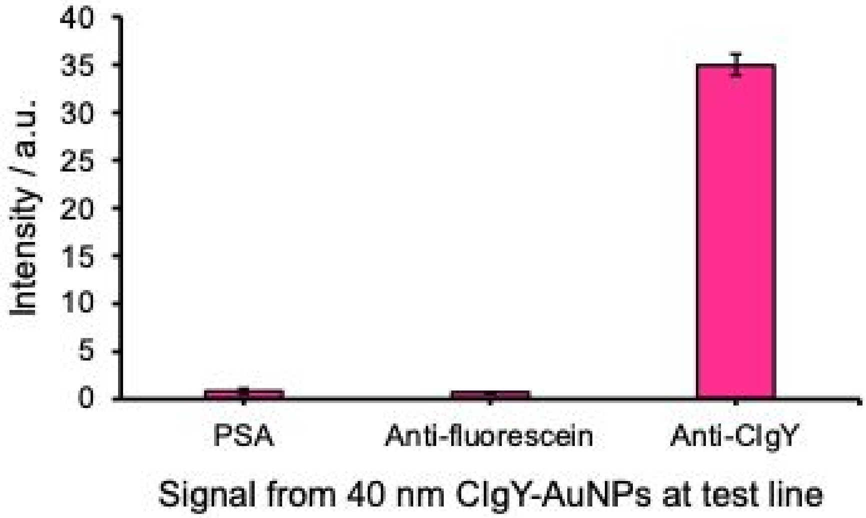

Figure S28: The mean intensities (N=3) of LFA test strip printed with either PSA (1 mg/mL), anti-fluorescein (3 mg/mL) or anti-CIgY (1 mgmL) test lines following the flowing up of 40 nm CIgY-AuNP conjugates (OD 0.1) in cleavage solution (50 mM DTT at pH 10).

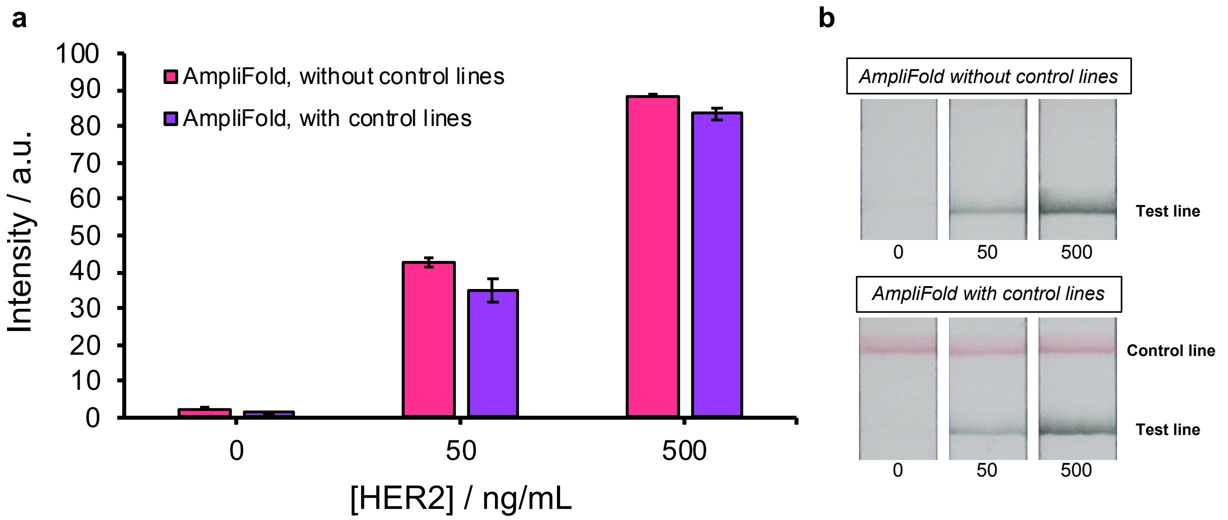

Figure S29: a The mean intensities (N=3) of detection strip anti-fluorescein (3 mg/mL) test lines following AmpliFold assays – detecting HER2 (0, 50, 500 ng/mL) – using capture and detection strips printed with, or without, anti-fluorescein and anti-CIgY control lines, respectively. **b** Photographic examples of AmpliFold detection test strips, with and without anti-CIgY control lines, when detecting HER2 (0, 50, or 500 ng/mL).

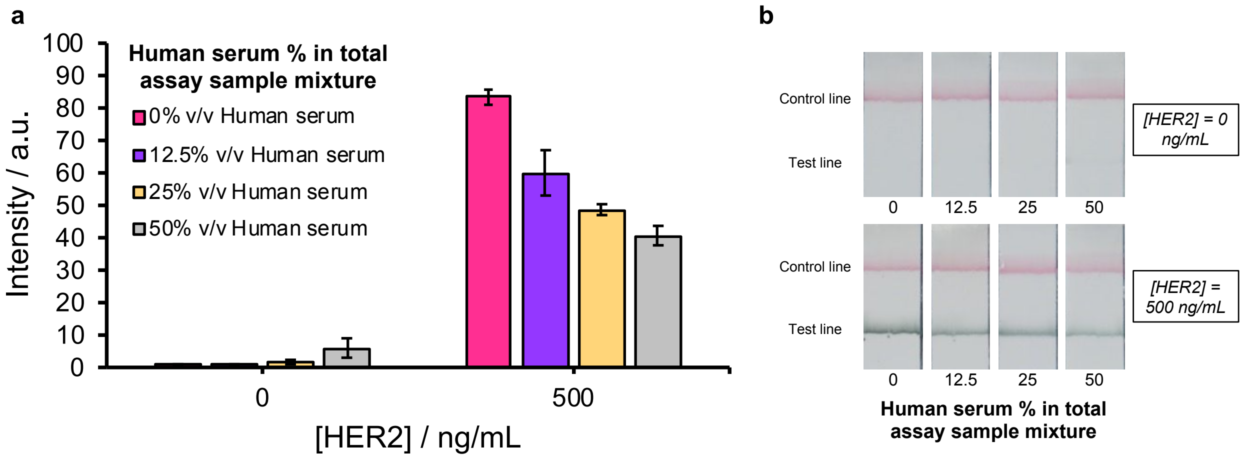

Figure S30: a The mean intensity (N=3) of detection strip anti-fluorescein (3 mg/mL) test lines in AmpliFold tests where the percentage of human serum within the total sample volume, run up test strips, was varied in AmpliFold capture-and-release' experiments. **b** Photographic examples of HER2 negative (0 ng/mL) and positive (500 ng/mL) detection strip test lines from AmpliFold experiments where the percentage of human serum within the total sample volume was varied.

LFA image analysis

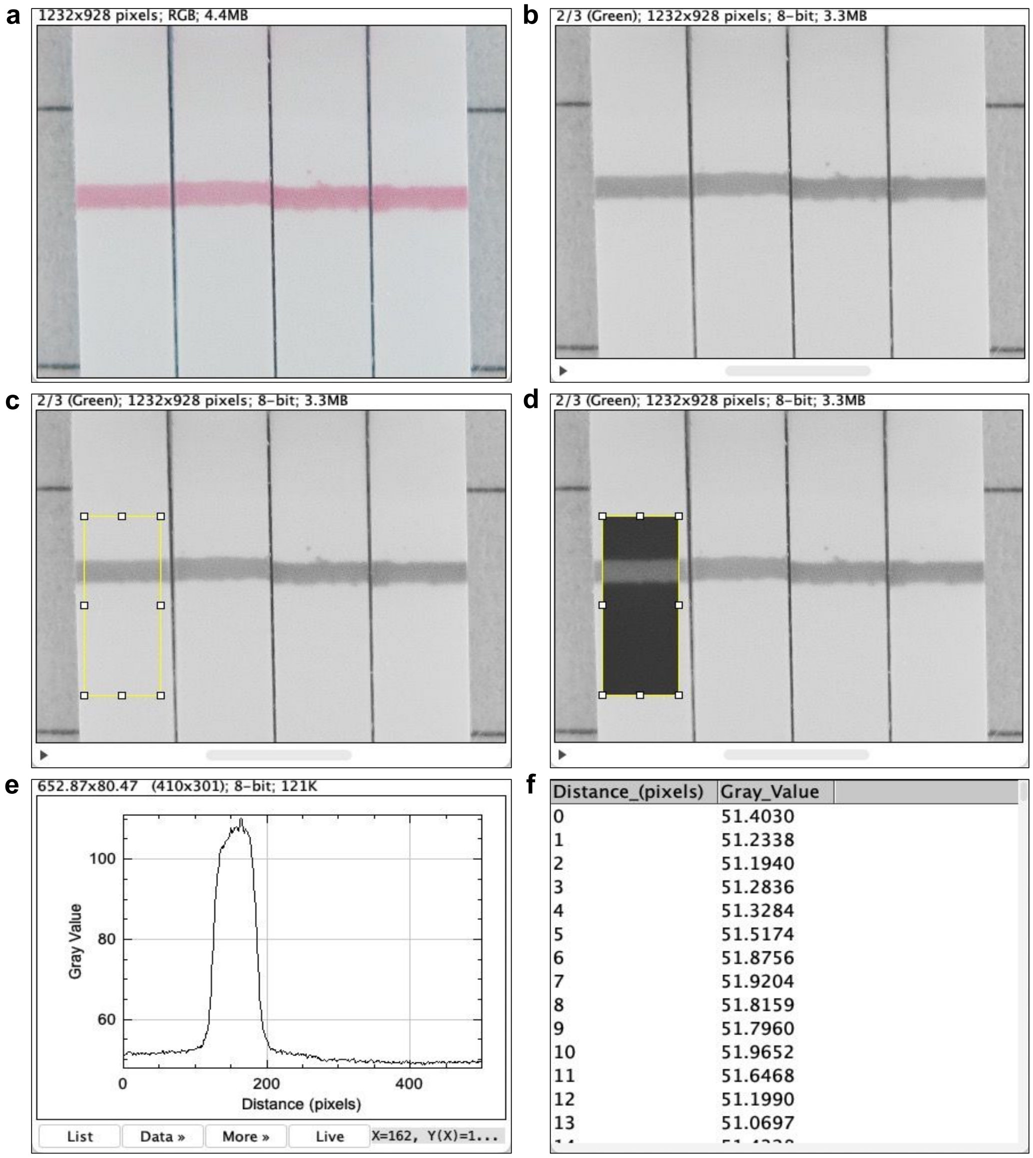

Figure S31: The procedure of LFA image analysis *via* ImageJ software using an example dataset. **a** JPEG image file containing LFA strips opened (File → Open) within the ImageJ software; **b** Conversion of image file into greyscale (Image → Type → RGB Stack) to allow signal to be viewed with maximum contrast. 'Red' nanoparticles, such as 20 and 40 nm AuNPs, were viewed using the green channel (Green), whilst green/blue nanoparticles, such as 150 nm AuNPs, were viewed in the red channel (Red); **c** A rectangular box (Rectangle tool), with an aspect ratio of 2:5 (width:length) was drawn to

capture a region of interest which contained both the test line. A region of interest was typically maintained as 200 (width) by 500 (length) pixels in order to capture a large representation of noise from the nitrocellulose membrane when during signal minus noise calculations; **d** A colour inversion operation (Ctrl + Shift + I) was performed on the region of interest; **e** The region of interest was plotted (Alt + K) such that the length of the strip was represented by the y-axis, whilst the x-axis comprised the mean average of pixel intensities across the width of the strip; **f** Data points of the plotted region of interest were generated (List) for subsequent signal minus noise calculations.

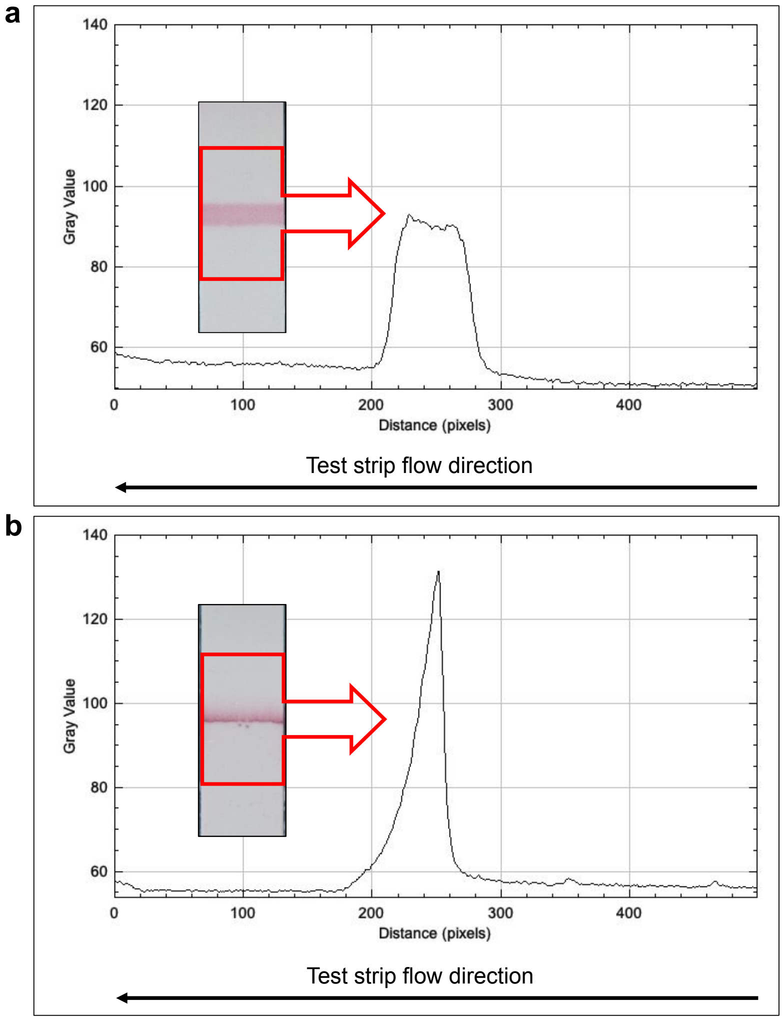

Figure S32: An exemplar depiction of the influence of test line signal distribution on plotted profiles during image analysis. **a** The plot of a test strip featuring a broad and

diffuse signal distribution at the test line. Signal permeates deeply into the printed region of the test line reflecting a relatively lower rate of association when capturing analyte-bound complexes; **b** The plot of a test strip where signal is concentrated at the front of the test line, yielding a sharp and narrow signal distribution with high signal-to-noise. Sharp signal reflects the fast binding of analyte-bound complexes approaching the printed test line region.

Supplementary methods

General

Throughout this work, LFA procedures were comprised of either half-stick or AmpliFold format experiments. Unless otherwise stated, unblocked strips were used when conducting pre-development and optimisation LFA experiments. Membrane blocked strips refer solely to the capture and traditional ‘half stick’ strips used in limit-of-detection (LOD) studies, but not detection strips. Capture strips printed with multiple PSA test lines are explicitly indicated with the number of test lines comprising the capture area (e.g. 3×, 6×, etc); unless described in this convention, single PSA-printed test line test strips were used.

Functionalised gold nanoparticles (AuNPs) were prepared and used in LFA studies exclusively in a blocking buffer composed of BSA (bovine serum albumin, 2% w/v) in borate buffer (pH 8, 100 mM) with Tween20 (0.1% v/v; Acros Organics). Unless otherwise stated, dithiothreitol (DTT; Roche) was prepared in cleavage buffer (pH 10 100 mM carbonate buffer with 0.1% v/v Tween20). In all cases, DTT was prepared in cleavage buffer immediately assay before use.

Printing of test lines for LFA strips

Printing of test lines onto FF80HP Plus membranes (Whatman) was performed using the AD1520™ Aspirate Dispense System (BioDot). Unless undergoing further printing with control lines or membrane blocking, printed membranes were attached with SureWick absorbent pads (Millipore) and dried (37 ˚C, 2 h) prior to being cut into 3 mm wide strips using a ZQ2002 Strip Cutter (Kinbio).

AmpliFold capture strips and traditional ‘half stick’ strips were printed with polystreptavidin (PSA, BBI Solutions) using a 0.6 µL/cm dispense rate, 350 µs open time and 15 nL drop size. Traditional ‘half stick’ LFA strips were printed with a single test line 13 mm from the bottom of the membrane. Capture strips were printed such that test lines were 1 mm apart. Relative to the bottom of the membrane: 1× capture strips were printed with a test line at 13 mm; 3× capture strips were printed with test lines at 12, 13, and 14 mm; 6×

capture strips were printed with test lines at 10, 11, 12, 13, 14, and 15 mm; 9× capture strips were printed with test lines at 7, 8, 9, 10, 11, 12, 13, 14, and 15 mm.
AmpliFold detection strips were printed using anti-fluorescein polyclonal antibody (pAb) (abcam) using a 1 µL/cm dispense rate, 550 µs open time and 40 nL drop size. Test lines were printed 13 mm from the bottom of the membrane. Prior to LFA use, detection strips were shortened by cutting 7 mm from the bottom of the membrane for the purposes of AmpliFold assembly.

Printing of control lines for LFA strips
Printing of test lines onto FF80HP Plus membranes (Whatman) was performed using the AD1520™ Aspirate Dispense System (BioDot). Unless undergoing membrane blocking, printed membranes were attached with SureWick absorbent pads (Millipore) and dried (37 ˚C, 2 h) prior to being cut into 3 mm wide strips using a ZQ2002 Strip Cutter (Kinbio).

AmpliFold capture strips and traditional 'half stick' LFA strips were printed with anti-fluorescein pAb (1 mg/mL) control lines – using a 0.6 µL/cm dispense rate, 350 µs open time and 15 nL drop size – 18 mm from the bottom of the membrane.
AmpliFold Detection strips were printed using anti-CIgY pAb (1 mg/mL; Jackson ImmunoResearch) control lines – using a 1 µL/cm dispense rate, 550 µs open time and 40 nL drop size – 18 mm from the bottom of the membrane. Prior to LFA use, detection strips were shortened by cutting 7 mm from the bottom of the membrane for the purposes of AmpliFold assembly.

Membrane blocking procedure for capture strips
PSA-printed capture strips (3 mm width; FF80HP Plus) were temporarily fitted, at the 'top' of the strip with absorbent pad (21 mm in length) using adhesive tape. Strips were then allowed to wick membrane blocking solution (BSA in pH 8 100 mM borate buffer), through minimally contacting the end of the strip to the solution, for 2 minutes seconds prior to removal of the absorbent pad and full submersion of the membrane. After one hour of blocking, membranes were then dried (37 ˚C, 2 h) and then temporarily fitted, at the 'top' of the strip with absorbent pad (21 mm in length) using adhesive tape. Strips were allowed

to wick membrane washing solution, through minimally contacting the end of the strip to the solution, for 3 minutes prior to strip drying (37 ˚C, 2 h).

Preparation of 'dual-affinity' 20 nm FluoroPerAuNP conjugates

In a typical procedure, to an aqueous solution of FluoroPer (190 µg/mL, 100 µL), borate buffer (pH 8, 100 mM, 100 µL) and 20 nm AuNP solution (OD 1, 1 mL) were added sequentially prior to incubation under agitation (21 ˚C, 1 h, 600 rpm). Following this, the physisorption mixture was blocked with blocking buffer (2% w/v BSA in borate buffer (pH 8, 100 mM) with 0.1% v/v Tween20, 1 mL) and further incubated under agitation (21 ˚C, 1 h, 600 rpm). Blocked mixtures were then centrifuged (15,000 rcf, 10 min), forming a free-flowing pellet of colloid from which supernatant was extracted. The colloidal pellet was then washed by re-suspension in blocking buffer (2 mL) and then centrifuged (15,000 rcf, 10 min) prior to supernatant extraction. This washing procedure was repeated twice more prior to the characterisation of 20 nm FluoroPerAuNP conjugates.

Preparation of 'dual-affinity' 40 nm FluoroPerAuNP conjugates

In a typical procedure, to an aqueous solution of FluoroPer (68 µg/mL, 100 µL), borate buffer (pH 8, 100 mM, 100 µL) and 40 nm AuNP solution (OD 1, 1 mL) were added sequentially prior to incubation under agitation (21 ˚C, 1 h, 600 rpm). Following this, the physisorption mixture was blocked with blocking buffer (2% w/v BSA in borate buffer (pH 8, 100 mM) with 0.1% v/v Tween20, 1 mL) and further incubated under agitation (21 ˚C, 1 h, 600 rpm). Blocked mixtures were then centrifuged (2500 rcf, 15 min), forming a free-flowing pellet of colloid from which supernatant was extracted. The colloidal pellet was then washed by re-suspension in blocking buffer (2 mL) and then centrifuged (2500 rcf, 15 min) prior to supernatant extraction. This washing procedure was repeated twice more prior to the characterisation of 40 nm FluoroPerAuNP conjugates.

Preparation of 'dual-affinity' 150 nm FluoroPerAuNP conjugates

In a typical procedure, to an aqueous solution of FluoroPer (165 µg/mL, 100 µL), borate buffer (pH 8, 100 mM, 100 µL) and 150 nm AuNP solution (OD 10, 1 mL) were added sequentially prior to incubation under agitation (21 ˚C, 1 h, 650 rpm). Following this, the

physisorption mixture was blocked with blocking buffer (2% w/v BSA in borate buffer (pH 8, 100 mM) with 0.1% v/v Tween20, 1 mL) and further incubated under agitation (21 ˚C, 1 h, 650 rpm). Blocked mixtures were then centrifuged (2500 rcf, 10 min), forming a free-flowing pellet of colloid from which supernatant was extracted. The colloidal pellet was then washed by re-suspension in blocking buffer (2 mL) and then centrifuged (2500 rcf, 10 min) prior to supernatant extraction. This washing procedure was repeated twice more prior to the characterisation of 150 nm FluoroPerAuNP conjugates.

Preparation of 'model affinity' 40 nm biotinylated-AuNPs

In a typical procedure, lipoic acid-PEG5K-biotin (100 mM, 100 µL; Nanocs) was added to 40 nm AuNP solution (OD 1, 1 mL) and incubated under agitation (21 ˚C, 1 h, 600 rpm). Following this, the physisorption mixture was blocked with blocking buffer (2% w/v BSA in borate buffer (pH 8, 100 mM) with 0.1% v/v Tween20, 1 mL) and further incubated under agitation (21 ˚C, 1 h, 600 rpm). Blocked mixtures were then centrifuged (2500 rcf, 15 min) forming a free-flowing pellet of colloid from which supernatant was extracted. The colloidal pellet was then washed by re-suspension in blocking buffer (2 mL) and then centrifuged (2500 rcf, 15 min) prior to supernatant extraction. This washing procedure was repeated twice more prior to the characterisation of 40 nm biotinylated-AuNPs.

Preparation of 40 nm CIgY-AuNP conjugates

In a typical procedure, to an aqueous solution of chicken IgY antibody (CIgY, 79.2 µg/mL, 100 µL; Jackson ImmunoResearch), borate buffer (pH 8, 100 mM, 100 µL) and 40 nm AuNP solution (OD 1, 1 mL) were added sequentially prior to incubation under agitation (21 ˚C, 1 h, 600 rpm). Following this, the physisorption mixture was blocked with blocking buffer (2% w/v BSA in borate buffer (pH 8, 100 mM) with 0.1% v/v Tween20, 1 mL) and further incubated under agitation (21 ˚C, 1 h, 600 rpm). Blocked mixtures were then centrifuged (2500 rcf, 15 min), forming a free-flowing pellet of colloid from which supernatant was extracted. The colloidal pellet was then washed by re-suspension in blocking buffer (2 mL) and then centrifuged (2500 rcf, 15 min) prior to supernatant extraction. This washing procedure was repeated twice more prior to the characterisation of 40 nm CIgY-AuNP conjugates.

Mass spectrometry observed linker cleavage

DTT (0, 0.25, 2.5, 25, and 50 mM, 4 µL, 3 mM EDTA [ethylenediaminetetraacetic acid] solution), were added to a solution of Fab-SS-biotin conjugate **(2)** (5 µM, 26 µL, 50 mM pH 9.0 borate buffer) and allowed to incubate (21˚C, 10 min). After this period, MES buffer (2-(N-morpholino)ethanesulfonic acid, pH 6.5, 1 M, 5 µL) and *N*-methylmaleimide (100 mM, 5 µL, Milli-Q water; Acros Organics) were added and incubated (21˚C, 1 min) to quench the reaction. The mixture was immediately purified using a ZebaSpin™ desalting column (7 KDa molecular weight cut-off) and characterised by LC-MS.

Comparing the sensitivity of 40 nm FluoroPerAuNP conjugates and biotinylated-AuNPs detection in LFAs

To a microwell plate, 40 nm FluoroPerAuNP conjugates (OD 1, 0.5, 0.25, 0.125, 0.063, 0.031, 0.016, 0.008; 10 µL) were added, prior to the addition of anti-fluorescein pAb printed (1 mg/mL) test strips which were allowed to wick (5 min). Test strips were added to wells containing wash solution (10 µL, 0.5% v/v IGEPAL in Milli-Q water) and allowed to wick (5 min).

To other wells, 40 nm biotinylated-AuNPs (OD 1, 0.5, 0.25, 0.125, 0.063, 0.031, 0.016, 0.008; 10 µL) were added, prior to the addition of PSA-printed (1 mg/mL) test strips which were allowed to wick (5 min). Test strips were added to wells containing IGEPAL wash solution (10 µL, 0.5% v/v IGEPAL in Milli-Q water) and allowed to wick (5 min).

Stability and functionality testing of 40 nm FluoroPerAuNP conjugates under cleavage conditions in LFAs

To a microwell plate, DTT (0, 56, 112 mM; 18 µL; in cleavage buffer) was added. To this, 40 nm FluoroPerAuNP conjugates (OD 10, 2 µL) were added and mixed thoroughly. To each well, anti-fluorescein pAb printed (1 mg/mL) test strips were added and allowed to wick (10 minutes). Strips were then added to wells containing quenching solution (100 mM *N*-methylmaleimide in 500 mM pH 6.5 MES buffer, 10 µL).

Optimisation of cleavage buffer pH for linker cleavage in LFA

Fab_{HER} conjugate and HER2 (abcam) were prepared in a blocking buffer composed of 2% w/v BSA in borate buffer (pH 8, 100 mM) with 0.1 % v/v Tween20. In this experiment, the composition of the cleavage buffer varied between either Milli-Q water, pH 6.0 100 mM MES buffer, pH 7.0 100 mM HEPES buffer (2-[4-(2-hydroxyethyl)piperazin-1-yl]ethanesulfonic acid), pH 8.0 100 mM borate buffer, pH 9.0 100 mM carbonate buffer, or pH 10.0 100 mM carbonate buffer. In all cases, cleavage buffers also contained 3 mM EDTA and 0.1% v/v Tween. Quenching solution was comprised of *N*-methylmaleimide (100 mM) in MES buffer (pH 6.5, 500 mM).

To a microwell plate, 40 nm FluoroPerAuNP conjugates (OD 2, 10 µL), Fab_{HER}-PD-PEG_3-SS-PEG_4-biotin conjugates **4** (3000 ng/mL, 5 µL) and HER2 (2500 ng/mL, 5 µL) were added and pre-mixed (5 min). To each well, PSA-printed (1 mg/mL) test strips were added and allowed to wick (5 min). Test strips were added to wells containing Tween20 wash solution (0.1% v/v in Milli-Q water, 10 µL) and allowed to wick (5 min). Following this, test strips were then added to wells containing DTT (50 mM, 20 µL, prepared in either Milli-Q water or pH 6-10 cleavage buffer) and allowed to wick (10 min). Test strips were then quenched in quenching solution (10 µL).

Linker cleavage efficiency LFA studies

In these studies, Fab_{HER}-biotin conjugates refer generically to the testing of either Fab_{HER}-PEG_{12}-biotin **(1)**, Fab_{HER}-SS-biotin **(2)**, Fab_{HER}-SS-PEG_4-biotin **(3)**, or Fab_{HER}-PD-PEG_3-SS-PEG_4-biotin **(4)**. Fab_{HER} conjugates and HER2 (abcam) were prepared using a blocking buffer comprised of BSA (2% w/v) in borate buffer (pH 8, 100 mM) with Tween20 (0.1% v/v). Quenching solution was comprised of *N*-methylmaleimide (100 mM) in MES buffer (pH 6.5, 500 mM).

To a microwell plate, 40 nm FluoroPer-AuNP conjugates (OD 2, 10 µL), Fab_{HER}-biotin conjugates (3000 ng/mL, 5 µL) and HER2 (2500 ng/mL, 5 µL) were added and pre-mixed (5 min). To each well, PSA-printed (1 mg/mL) test strips were added and allowed to wick (5 min). Strips were added into wells containing Tween20 wash solution (0.1% v/v, 10 µL) and allowed to wick (5 min). Strips were then added into wells containing DTT (0, 20, 40,

60, 80 and 100 mM, 15 µL) and cleaved (10 min). Test strips were then quenched into wells containing quenching solution (10 µL).

Identification of particle membrane fouling during antigen-negative ‘capture-and-release’ AmpliFold assays

Fab_{HER} conjugates and HER2 (abcam) were prepared using a blocking buffer comprised of BSA (2% w/v) in borate buffer (pH 8, 100 mM). Quenching solution was comprised of *N*-methylmaleimide (100 mM) in MES buffer (pH 6.5, 500 mM).

To a microwell plate, running buffer (5 µL), Fab_{HER}-PD-PEG_3-SS-PEG_4-biotin (3000 ng/mL, 5 µL) and 40 nm FluoroPerAuNP conjugates (OD 2, 10 µL) were added and pre-mixed (5 min). To each well, 6× PSA test line printed (0, 0.1, 0.5 or 1 mg/mL) capture strips were added and allowed to wick (5 min). Test strips were added to wells containing Tween20 wash solution (0.1% v/v, 10 µL) and allowed to wick (5 min). Strips were then assembled into the AmpliFold format using anti-fluorescein pAb (1 mg/mL) detection strips and added into wells containing DTT (60 mM, 20 µL) and allowed to wick (10 min). AmpliFold tests were then quenched in wells containing quenching solution (10 µL).

Optimisation of BSA% in capture strip membrane blocking for the reduction of membrane fouling in ‘capture-and-release’ AmpliFold assays

Fab_{HER} conjugates and HER2 (abcam) were prepared using a blocking buffer comprised of BSA (2% w/v) in borate buffer (pH 8, 100 mM). Quenching solution was comprised of *N*-methylmaleimide (100 mM) in MES buffer (pH 6.5, 500 mM).

To a microwell plate, HER2 (0, 50 and 500 ng/mL, 5 µL), Fab_{HER}-PD-PEG_3-SS-PEG_4-biotin (3000 ng/mL, 5 µL) and 40 nm FluoroPerAuNP conjugates (OD 2, 10 µL) were added and pre-mixed (5 min). To each well, 6× PSA test line printed (1 mg/mL) capture strips (unblocked or 0.5, 1, or 2% w/v BSA blocked) were added and allowed to wick (5 min). Following this, strips were added into wells containing IGEPAL wash solution (0.5% v/v, 10 µL) and allowed to wick (5 min). Strips were then assembled into an AmpliFold format using anti-fluorescein pAb (1 mg/mL) detection strips and added into wells

containing DTT (60 mM, 20 µL) and allowed to wick (10 min). AmpliFold tests were then quenched in wells containing quenching solution (10 µL).

Optimisation of assay blocking buffer towards the reduction of membrane fouling in 'capture-and-release' AmpliFold assays

To compare the use of skimmed milk as a blocking protein, 0.2, 1 and 2% w/v skimmed milk were prepared in borate buffer (pH 8, 100 mM). Separately, 2 % w/v BSA was also prepared in borate buffer (pH 8, 100 mM). BSA and skimmed milk containing blocking buffers were used for the preparation of Fab_{HER} conjugates and HER2 (abcam).

To a microwell plate, HER2 (0, 50, 500 ng/mL, 5 µL), Fab_{HER}-PD-PEG_3-SS-PEG_4-biotin (3000 ng/mL, 5 µL) and 40 nm FluoroPerAuNP conjugates (OD 2, 10 µL) were added and pre-mixed (5 min). To each well, 6× PSA test line printed (1 mg/mL) capture strips were added and allowed to wick (5 min). Test strips were added to wells containing IGEPAL wash solution (0.5% v/v, 10 µL) and allowed to wick (5 min). Strips were then assembled into an AmpliFold format using anti-fluorescein pAb (1 mg/mL) detection strips and added into wells containing DTT (60 mM, 20 µL).

Optimisation of number of wash steps employed during 'capture-and-release' AmpliFold assays for the reduction of membrane fouling

Fab_{HER} conjugate and HER2 (ACROBiosystems) were prepared using a blocking buffer composed of 2% w/v skimmed milk in borate buffer (pH 8, 100 mM).

To a microwell plate, HER2 antigen (0, 50 ng/mL, 5 µL), Fab_{HER}-PD-PEG_3-SS-PEG_4-biotin (3000 ng/mL, 5 µL) and 40 nm FluoroPerAuNP conjugates (OD 2, 10 µL) were added and pre-mixed (5 min). To each well, 6× PSA-printed test line (1 mg/mL) capture strips were added and allowed to wick (5 min). Test strips were added to wells containing IGEPAL wash solution (0.5% v/v, 10 µL) and allowed to wick (5 min). This step was performed such that tests were washed a total of 1, 2 or 3 times. Strips were then assembled into an AmpliFold format using anti-fluorescein pAb printed (1 mg/mL) detection strips and added into wells containing DTT (60 mM, 20 µL).

Measurements of capture, cleavage and detection test line signals across a 'capture-and-release' AmpliFold workflow

Fab_{HER} conjugate and HER2 (ACROBiosystems) were prepared in a blocking buffer composed of 2% w/v skimmed milk in borate buffer (pH 8, 100 mM). To measure the test line signal of the uncleaved capture strip test line, parallel experiments were performed, based on the following procedure, omitting the AmpliFold assembly and cleavage step.

To a microwell plate, HER2 (0, 50, 500 ng/mL, 5 µL), Fab_{HER}-PD-PEG_3-SS-PEG_4-biotin (3000 ng/mL, 5 µL) and 40 nm FluoroPerAuNP conjugates (OD 2, 10 µL) were added and pre-mixed (5 min). To each well, PSA-printed (1 mg/mL) capture strips were added and allowed to wick (5 min). Test strips were added to wells containing IGEPAL wash solution (0.5% v/v, 3×10 µL) and allowed to wick (3×5 min). Strips were then assembled into an AmpliFold format using anti-fluorescein pAb printed (1 mg/mL) detection strips and added into wells containing DTT (60 mM, 20 µL). Strips were allowed to dry (21 ˚C, 1 h) and dissembled from the AmpliFold format, allowing the cleaved test line and the detection test line to be imaged.

Capture affinity studies on signal distribution over multi-test line LFAs through PSA test line concentration modulation

Fab_{HER} conjugates and HER2 (ACROBiosystems) were prepared in a blocking buffer composed of 2% w/v semi-skimmed milk in borate buffer (pH 8, 100 mM).

To a microwell plate, HER2 (500 ng/mL, 5 µL), Fab_{HER}-PD-PEG_3-SS-PEG_4-biotin conjugates (3000 ng/mL, 5 µL) and FluoroPerAuNP conjugates (10 µL) were added and pre-mixed (5 min). To each well, 6× PSA-printed test line (0.1, 0.5, 1 mg/mL) test strips were added and allowed to wick (5 min). Strips were added into wells containing IGEPAL wash solution (0.5% v/v, 3×10 µL) and allowed to wick (3×5 min).

Dose-response comparison between traditional LFA and 'capture-and-release' AmpliFold formats in a model affinity system with ideally distributed analyte capture

Fab_{HER} conjugates and HER2 (ACROBiosystems) were prepared in a blocking buffer composed of 2% w/v semi-skimmed milk in borate buffer (pH 8, 100 mM). LFA experiments were performed using PSA-printed (0.1 mg/mL) capture strips and traditional half stick strips prepared with membrane blocking procedures.

To a microwell plate, HER2 (0, 7.8, 15.6, 31.3, 62.5, 125, 500 and 1000 ng/mL, 5 µL; ACROBiosystems), Fab_{HER}-PD-PEG_3-SS-PEG_4-biotin (3000 ng/mL, 5 µL) and 40 nm FluoroPerAuNP conjugates (OD 2, 10 µL) were added and pre-mixed (5 min).

As a traditional LFA 'uncleaved' control, PSA-printed (0.1 mg/mL) test strips (BSA membrane blocked) were added and allowed to wick (5 min). Traditional LFA strips were then added to wells containing IGEPAL solution (0.5% v/v, 3×10 µL) and allowed to wick (3×5 min).

For 'capture-and-release' AmpliFold tests, multiple test line (1, 3, 6, or 9×) PSA-printed (0.1 mg/mL) capture strips (BSA membrane blocked) were added and allowed to wick (5 min). Capture strips were then added to wells containing IGEPAL wash solution (0.5% v/v, 3×10 µL) and allowed to wick (3×5 min). Capture strips were then assembled into AmpliFold formats using anti-fluorescein pAb printed (1 mg/mL) detection strips prior to addition into wells containing DTT (60 mM, 20 µL) and allowed to wick.

Study of particle size on capture affinity by evaluating signal distribution over multi-test line LFAs

Fab_{HER} conjugates and HER2 (ACROBiosystems) were prepared in a blocking buffer composed of 2% w/v semi-skimmed milk in borate buffer (pH 8, 100 mM). FluoroPerAuNP conjugates in the following procedure refers generically to the utilisation, and comparison, of 20 nm FluoroPerAuNP (OD 1), 40 nm FluoroPerAuNP (OD 2), and 150 nm FluoroPerAuNPs conjugates (OD 10).

To a microwell plate, HER2 (500 ng/mL, 5 µL), Fab_{HER}-PD-PEG_3-SS-PEG_4-biotin conjugates (3000 ng/mL, 5 µL), and FluoroPerAuNPs (10 µL) were added and pre-mixed (5 min). To each well, 9× PSA-printed (1 mg/mL) test line test strips were added and allowed to wick (5 min). Strips were added into wells containing IGEPAL wash solution (0.5% v/v, 3×10 µL) and allowed to wick (3×5 min).

Optimisation of detection strip anti-fluorescein pAb test line concentration in 'capture-and-release' AmpliFold assays

Fab_{HER}-biotin conjugates and HER2 (ACROBiosystems) were prepared in a blocking buffer composed of 2% w/v semi-skimmed milk in borate buffer (pH 8, 100 mM).

To a microwell plate, HER2 (0, 5, 50, 500 ng/mL, 5 µL), Fab_{HER}-PD-PEG_3-SS-PEG_4-biotin (5000 ng/mL, 5 µL) and 150 nm FluoroPerAuNP conjugates (OD 10, 10 µL) were added and pre-mixed (5 min). To each well, 9× PSA-printed (1 mg/mL) test line capture strips were added and allowed to wick (5 min). Strips were added into wells containing IGEPAL wash solution (0.5% v/v, 3×10 µL) and allowed to wick (3×5 min). Capture strips were then assembled into an AmpliFold format using anti-fluorescein pAb printed (1, 3, or 5 mg/mL) detection strips and added into wells containing DTT (50 mM, 20 µL).

Dose-response comparison between traditional LFA and 'capture-and-release' AmpliFold formats in large particle systems

Fab_{HER} conjugates and HER2 (ACROBiosystems) were prepared in a blocking buffer composed of 2% w/v semi-skimmed milk in borate buffer (pH 8, 100 mM). LFA experiments were performed using PSA-printed (1 mg/mL) capture strips and traditional half stick strips prepared with membrane blocking procedures.

To a microwell plate, HER2 (0, 0.03, 0.12, 0.49, 1.95, 7.8, 31.3, 125 and 500 ng/mL, 5 µL), Fab_{HER}-PD-PEG_3-SS-PEG_4-biotin (5000 ng/mL, 5 µL) and 150 nm FluoroPerAuNP conjugates (OD 10, 10 µL) were added and pre-mixed (5 min).

As a traditional LFA 'uncleaved' control, PSA-printed (1 mg/mL) test strips were added and allowed to wick (5 min). Traditional LFA strips were then added to wells containing IGEPAL solution (0.5% v/v, 3×10 µL) and allowed to wick (3×5 min).

For 'capture-and-release' AmpliFold assays, 9× PSA-printed (1 mg/mL) test line capture strips were added and allowed to wick (5 min). Capture strips were then added to wells containing IGEPAL wash solution (0.5% v/v, 3×10 µL) and allowed to wick (3×5 min). Capture strips were then assembled into an AmpliFold format using anti-fluorescein pAb printed (3 mg/mL) detection strips and added into wells containing DTT (50 mM, 20 µL).

Titration of total human serum % in traditional and AmpliFold LFA assay sample mixtures

LFA experiments were performed using PSA-printed (1 mg/mL) capture strips and traditional half stick strips prepared with membrane blocking procedures.

To achieve 0% v/v human serum in the assay sample mixture, Fab_{HER} conjugates and HER2 (ACROBiosystems) were prepared solely in a blocking buffer (composed of 2% w/v semi-skimmed milk in pH 8 100 mM borate buffer), prior to mixing in a microwell plate with 150 nm FluoroPerAuNP conjugates. To achieve 12.5% v/v human serum in the assay sample mixture, Fab_{HER} conjugates were prepared in a blocking buffer whilst HER2 was prepared in a 1:1 mixture of blocking buffer and human serum (Merck), prior to mixing in a microwell plate with 150 nm FluoroPerAuNP conjugates. To achieve 25% v/v human serum in the assay sample mixture, Fab_{HER} conjugates were prepared in a blocking buffer whilst HER2 was prepared entirely in human serum, prior to mixing in a microwell plate with 150 nm FluoroPerAuNP conjugates. To achieve 50% v/v human serum in the assay sample mixture, both Fab_{HER} conjugates and HER2 was prepared in human serum, prior to mixing in a microwell plate with 150 nm FluoroPerAuNP conjugates.

To a microwell plate, HER2 (0, 50, and 500 ng/mL, 5 µL), Fab_{HER}-PD-PEG_3-SS-PEG_4-biotin (3000 ng/mL, 5 µL) and 150 nm FluoroPerAuNP conjugates (OD 10, 10 µL) were added and pre-mixed (5 min).

As a traditional LFA ‘uncleaved’ control, PSA-printed (1 mg/mL) test strips were added and allowed to wick (5 min). Traditional LFA strips were then added to wells containing IGEPAL solution (0.5% v/v, 10 µL) and allowed to wick (5 min).

For ‘capture-and-release’ AmpliFold assays, 9× PSA-printed (1 mg/mL) test line capture strips were added and allowed to wick (5 min). Capture strips were then added to wells containing IGEPAL wash solution (0.5% v/v, 3×10 µL) and allowed to wick (3×5 min). Capture strips were then assembled into an AmpliFold format using anti-fluorescein pAb printed (3 mg/mL) detection strips and added into wells containing DTT (50 mM, 20 µL).

Stability and functionality of 40 nm CIgY-AuNPs in direct bind LFAs

In this experiment, blocking buffer is composed of 2% w/v BSA in borate buffer (pH 8, 100 mM) with 0.1% v/v Tween20, cleavage buffer refers to a composition of 0.1% v/v Tween20 in carbonate buffer (pH 10, 100 mM), and cleavage solution refers to a composition of 50 mM DTT in cleavage buffer.

To a microwell plate, 18 µL of either blocking buffer, cleavage buffer, or cleavage solution was added. Into these wells, 40 nm CIgY-AuNP conjugates (OD 1, 2 µL) were added and mixed thoroughly, prior to the addition of anti-CIgY pAb printed (1 mg/mL) test strips.

Stability and functionality of 40 nm CIgY-AuNP conjugates in direct bind LFAs

To a microwell plate, 18 µL of cleavage solution (50 mM DTT in pH 10 100 mM carbonate buffer with 0.1% v/v Tween20) was added. Into these wells, 40 nm CIgY-AuNP conjugates (OD 1, 2 µL) were added and mixed thoroughly, prior to the addition of test strips printed with either PSA (1 mg/mL), anti-fluorescein pAb (3 mg/mL), or anti-CIgY pAb (1 mg/mL) test lines.

Dose-response comparison between traditional LFA and ‘capture-and-release’ AmpliFold formats when detecting HER2 spiked into human serum

LFA experiments were performed using PSA-printed (1 mg/mL) capture strips and traditional half stick strips prepared with membrane blocking procedures. All AmpliFold and traditional LFA test strips used in this experiment were prepared with control lines.

In this experiment, Fab_{HER} conjugates were prepared in a blocking buffer (composed of 2% w/v semi-skimmed milk in pH 8 100 mM borate buffer) whilst HER2 (ACROBiosystems) was prepared human serum (Merck).

To a microwell plate, HER2 (0, 0.49, 1.95, 7.8, 15.6, 31.3, 125, and 500 ng/mL, 2.5 µL), Fab_{HER}-PD-PEG_3-SS-PEG_4-biotin (3000 ng/mL, 7.5 µL) and 150 nm FluoroPerAuNP conjugates (OD 10, 10 µL) were added and pre-mixed (5 min).

As a traditional LFA 'uncleaved' control, PSA-printed (1 mg/mL) test strips were added and allowed to wick (5 min). Traditional LFA strips were then added to wells containing IGEPAL solution (0.5% v/v, 10 µL) and allowed to wick (5 min).

For 'capture-and-release' AmpliFold assays, 9× PSA-printed (1 mg/mL) test line capture strips were added and allowed to wick (5 min). Capture strips were then added to wells containing IGEPAL wash solution (0.5% v/v, 3×10 µL) and allowed to wick (3×5 min). Capture strips were then assembled into an AmpliFold format using anti-fluorescein pAb printed (3 mg/mL) detection strips and added into wells containing DTT (50 mM, 20 µL).

<u>References</u>

(1) Szychowski, J.; Mahdavi, A.; Hodas, J. J. L.; Bagert, J. D.; Ngo, J. T.; Landgraf, P.; Dieterich, D. C.; Schuman, E. M.; Tirrell, D. A. Cleavable Biotin Probes for Labeling of Biomolecules via Azide-Alkyne Cycloaddition. *J. Am. Chem. Soc.* **2010**, *132* (51), 18351–18360. https://doi.org/10.1021/ja1083909.

(2) Forte, N.; Benni, I.; Karu, K.; Chudasama, V.; Baker, J. R. Cysteine-To-Lysine Transfer Antibody Fragment Conjugation. *Chem. Sci.* **2019**, *10* (47), 10919–10924. https://doi.org/10.1039/c9sc03825f.
